# Epigenetic modification of cytosines fine tunes the stability of i-motif DNA

**DOI:** 10.1093/nar/gkz1082

**Published:** 2019-11-28

**Authors:** Elisé P Wright, Mahmoud A S Abdelhamid, Michelle O Ehiabor, Melanie C Grigg, Kelly Irving, Nicole M Smith, Zoë A E Waller

**Affiliations:** 1 School of Pharmacy, University of East Anglia, Norwich Research Park, Norwich NR4 7TJ, UK; 2 Centre for Molecular and Structural Biochemistry, University of East Anglia, Norwich Research Park, Norwich NR4 7TJ, UK; 3 School of Molecular Sciences, University of Western Australia, 35 Stirling Hwy, Crawley, WA 6009, Australia

## Abstract

i-Motifs are widely used in nanotechnology, play a part in gene regulation and have been detected in human nuclei. As these structures are composed of cytosine, they are potential sites for epigenetic modification. In addition to 5-methyl- and 5-hydroxymethylcytosine modifications, recent evidence has suggested biological roles for 5-formylcytosine and 5-carboxylcytosine. Herein the human telomeric i-motif sequence was used to examine how these four epigenetic modifications alter the thermal and pH stability of i-motifs. Changes in melting temperature and transitional pH depended on both the type of modification and its position within the i-motif forming sequence. The cytosines most sensitive to modification were next to the first and third loops within the structure. Using previously described i-motif forming sequences, we screened the MCF-7 and MCF-10A methylomes to map 5-methylcytosine and found the majority of sequences were differentially methylated in MCF7 (cancerous) and MCF10A (non-cancerous) cell lines. Furthermore, i-motif forming sequences stable at neutral pH were significantly more likely to be epigenetically modified than traditional acidic i-motif forming sequences. This work has implications not only in the epigenetic regulation of DNA, but also allows discreet tunability of i-motif stability for nanotechnological applications.

## INTRODUCTION

**Figure 1.**
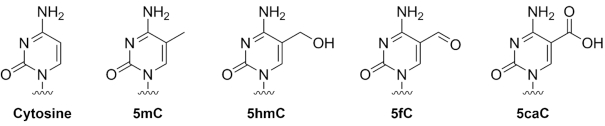
Chemical structures of cytosine and the examined epigenetic modifications. 5mC: 5-methylcytosine, 5hmC: 5-hydroxymethylcytosine; 5fC: 5-formylcytosine and 5caC: 5-carboxylcytosine.

The information available from DNA is multi-layered. Its raw sequence can code for proteins and regulatory elements (1), and emerging evidence has shown that secondary DNA structures can influence how and when genes are expressed (2,3). An additional level of information is added when epigenetic modification is considered. Epigenetics refers to information that is transmitted to daughter generations but is not based on DNA sequence (4). An example of this is DNA methylation, where cytosine bases are modified at the carbon-5 (C5) position with a methyl group. The methylation of cytosine can alter levels of gene expression resulting in heritable patterns of transcriptional silencing and genomic imprinting (5,6). Methylation arises at the C5 position on cytosine bases through DNA methyltransferase activity (6). Typically, 5′- to 3′-dinucleotide CpG sequences are targeted for methylation (7), and long unmethylated repeats of these sequences can form CpG islands (7,8). There is a correlation between methylation of CpG islands near transcription start sites and gene silencing (5), thus these are important in epigenetic control of gene expression. Throughout development, patterns of methylation change and it is not yet clear how bases are actively demethylated (9). However, ten eleven translocation (TET) dioxygenases are able to oxidize 5-methylcytosine (5mC) to 5-hydroxymethylcytosine (5hmC) which has also been associated with epigenetic labelling (Figure 1).

From here the stepwise oxidation of 5hmC can generate 5-formylcytosine (5fC) and then 5-carboxylcytosine (5caC) (Figure 1) (10). Once cytosine is modified from 5mC or 5hmC to 5caC, it can be recognized and excised from duplex DNA by thymine-DNA glycosylase (10). While 5mC and 5hmC have been readily detected in the genome (11), it was only recently that the TET family of dioxygenases were shown to produce 5fC and 5caC from 5mC via an enzymatic reaction *in vitro* (12). This has led to the detection of these modified bases in murine genomic DNA from embryonic stem cells and organs (9,10,12). Although initially it was thought that these bases were part of the natural metabolism of 5mC, they are now indicated to be epigenetic modifications in their own right. The majority of hmC is actually stable in the genomic DNA (13). 5fC can be a stable DNA modification in mammals (14), there are proteins that bind to 5fC in genomic sequences (15) and 5fC is a determinant of nucleosome organization and plays a role in establishing distinct regulatory regions that control transcription (16). The enzyme TET3 has been found to be a specific reader of 5caC at CcaCG sequences (17). Despite increasing interest and research in this area, there is still much to learn about these epigenetic modifications and their regulatory effects in biology.

As cytosine is a major epigenetic target, DNA secondary structures that assemble from cytosine-rich sequences may be affected by epigenetic modification (18). i-Motifs are DNA secondary structures made up of two intercalated hairpins held together by hemi-protonated cytosine:cytosine base pairs (19). These structures have been implicated in the regulation of DNA transcription and have recently been detected in human nuclei (2,3,20). The effect of epigenetic modification on the stability of i-motif DNA structures has been investigated using specific examples. Previous work has examined the effect on i-motif stability with the modification of an individual cytosine in a single position to a methyl- or hydroxymethylcytosine (18,21). This work indicated that methylation at a selection of sites resulted in a more stable i-motif while hydroxymethylation destabilized the structure (21). This correlates with the study reporting that 5mC reduces whereas 5hmC enhances the flexibility of DNA (22). Another study showed similar trends in stabilization with methylcytosine and destabilization with hydroxymethylcytosine (18). This work also examined different sites of individual modification and modification at multiple sites, but not every cytosine position in the sequence was investigated. Research by Yang *et al.* revealed that 5mC stabilizes DNA i‐motif conformations by increasing the base‐pairing energies relative to cytosine, whereas the opposite was observed for halogenated analogues (23). This is also corroborated by Lannes *et al.* (24) and indicates that the electronics of the bases are important in i-motif structure, with electron donating groups stabilizing the positive charge and electron-withdrawing groups having an opposite effect. Moreover, successive methylation of cytosines can result in an additive effect for increased stability of the overall structure (25).

Given the unexplored additional potential epigenetic modifications, 5fC and 5caC and the untested cytosine positions in the human telomeric sequence, herein we describe a systematic study to examine how these modifications alter i-motif stability and where they have the most impact on the stability of i-motif structure. UV spectroscopy, thermal difference spectra (TDS) and circular dichroism (CD) were used to explore the thermal and pH stability of the human telomeric i-motif sequence (hTeloC) when modified with a single methyl-, hydroxymethyl-, formyl- or carboxylcytosine. The outcomes of this work could be used in nanotechnological applications to tune the stability of i-motif and to further our understanding of the potential interplay between i-motif and epigenetic cytosine modification.

## MATERIALS AND METHODS

### Oligonucleotides

Oligodeoxynucleotides (ODNs) (Tables 1, Supplementary Tables S1 and S2) were supplied by Eurogentec (Belgium), synthesized on a 200 nmol scale and purified by reverse phase HPLC. ODNs from Table 1 were based on the hTeloC sequence 5′-TCC-CTA-ACC-CTA-ACC-CTA-ACC-CAA-3′ where the flanking sequence was modified slightly to facilitate the synthesis of the different analogues. Each sequence was modified at a single cytosine site with one of four epigenetic modifications (m, hm, f or ca) or mutated to thymine. Each ODN was labelled as ‘XXyCZZ’, where XX = ODN number (01-48); y = m, h, f or ca indicating the type of modification, C = cytosine and ZZ = which of the cytosines in the hTeloC sequence was modified (01–12); see Table 1 for full sequences and key. This nomenclature allows readers to identify the ODN by the number (e.g. 01) as well as the modification type (e.g. mC) and position of the modification (01, the first cytosine in the sequence). ODNs from Supplementary Table S1 were also based on the same hTeloC sequence and in this case each cytosine site was mutated to a thymine. Each ODN was labelled as ‘hTeloCTx’, where x = which of the cytosines in the sequence was modified. ODNs from Supplementary Table S2 were based on the genomic i-motifs MSMO1 5′-CCC-CCG-CCC-CCG-CCC-CCG-CCC-CC-3′ and PLCB2 5′-CCC-CCG-CCT-CTT-CTG-GAG-GCC-CCC-GCC-CCC-ACC-CCC-3′ (26). Each ODN was labelled as MSMO1-5xC or PLCB2-5xC, respectively, where x = m, hm, f or ca indicating modification. Here, the 15th cytosine in the MSMO1 sequence was modified and for PLCB2 both the 5th and the 14th cytosines were modified. These modifications are based on the methylation positions observed in the methylation profiles in MCF-7 (27) and MCF-10A (28). All ODNs were dissolved in ultra-pure water to give 100 μM final concentrations, which were confirmed using a Nanodrop. For all experiments, ODNs were diluted in buffer containing 10 mM sodium cacodylate and 100 mM sodium chloride at pH 5.5 unless otherwise indicated. ODNs were thermally annealed by heating in a heat block at 95°C for 5 min and cooled slowly to room temperature overnight.

**Table 1.** Modification of hTeloC sequence and key describing modification type and location

		Cytosine modification			
Sequence (5′-3′)	Cytosine	5mC	5hmC	5fC	5caC
T					
C	1	01mC01	13hmC01	25fC01	37caC01
C	2	02mC02	14hmC02	26fC02	38caC02
C	3	03mC03	15hmC03	27fC03	39caC03
T					
A	Loop 1				
A					
C	4	04mC04	16hmC04	28fC04	40caC04
C	5	05mC05	17hmC05	29fC05	41caC05
C	6	06mC06	18hmC06	30fC06	42caC06
T					
A	Loop 2				
A					
C	7	07mC07	19hmC07	31fC07	43caC07
C	8	08mC08	20hmC08	32fC08	44caC08
C	9	09mC09	21hmC09	33fC09	45caC09
T					
A	Loop 3				
A					
C	10	10mC10	22hmC10	34fC10	46caC10
C	11	11mC11	23hmC11	35fC11	47caC11
C	12	12mC12	24hmC12	36fC12	48caC12
A					
A					

### Circular dichroism

CD spectra were recorded on a Jasco J-810 spectropolarimeter using a 1 mm path length quartz cuvette. ODNs were diluted to 10 μM (total volume: 200 μl) in buffer at pH increments of 0.25 pH units from 5.0 to 8.0, depending on the sequence (26). The scans were recorded at room temperature between 200 and 320 nm. Data pitch was set to 0.5 nm and measurements were taken at a scanning speed of 200 nm/min, response time of 1 s, bandwidth of 2 nm and 100 mdeg sensitivity; each spectrum was the average of three scans. Samples containing only buffer were also scanned according to these parameters to allow for blank subtraction. Transitional pH (pH_T_) for each i-motif was calculated from the inflection point of fitted ellipticity at 288 nm.

### UV absorption spectroscopy

UV spectroscopy experiments were performed on a Cary 60 UV–Vis spectrometer (Agilent Technologies) equipped with a qChanger Temperature Controller (Quantum Northwest) and recorded using low volume masked quartz cuvettes (1 cm path length). ODNs were diluted to 2.5 μM in buffer at the desired pH. Samples (200 μL) were transferred to a cuvette, covered with a layer of silicone oil and stoppered to reduce evaporation of the sample. The absorbance of the ODN was measured at 295 nm as the temperature of the sample was held for 10 min at 4°C, heated to 95°C at a rate of 0.5°C per min, then held at 95°C for 10 min before the process was reversed; each melting/annealing process was repeated three times. Data were recorded every 1°C during both melting and annealing and melting temperatures (*T*_m_) were determined using the first derivative method. The change in enthalpy and entropy was calculated as described previously (29). Briefly, the measured absorbance versus temperature data was converted into fraction folded (θ) versus temperature. A plot of the natural logarithm of the affinity constant versus the reciprocal of the temperature in Kelvin is then produced and the thermodynamic parameters calculated from this; here the analysis was restricted to θ values between 0.15 and 0.85 where *K*_a_ values are most precise (30). This analysis was performed independently on each of the three melting curves collected for each ODN and the values presented are the average and standard deviation of these. TDS were calculated by subtracting the spectrum of the folded structure between 220 and 320 nm at 4°C from that of the unfolded structure at 95°C. The data was normalized and maximum change in absorption was set to +1 as previously described (31).

### Data analysis

Final analysis and presentation of the data was performed using Origin 2015. *P*-values were calculated by carrying out independent two-sample *t*-tests on the melting temperature and thermodynamic data collected from the triplicate melts for each oligonucleotide.

## RESULTS AND DISCUSSION

Biophysical characterization was used in order to assess how epigenetic modification influences the pH and thermal stability of the i-motif. The human telomeric i-motif sequence was used as a model for this study as it is well characterized, methylation in the telomere has known biological consequences (32,33) and also to complement prior work (18,24,25,34). A series of ODNs were purchased where each of the 12 cytosines in the hTeloC sequence were modified with one of four modifications (Table 1) resulting in 48 different variants.

The nature of the DNA secondary structure assembled from these epigenetically modified sequences was examined using CD. At pH 5.0, the unmodified control clearly showed a CD spectra that indicated an i-motif secondary structure in solution; a large positive peak at ∼288 nm and a negative peak at 255 nm (35). As the pH was increased to 7.0, the CD spectra shifted to reflect the loss of an organized DNA secondary structure and became more consistent with a random coil (35) with a smaller positive peak at ∼276 nm. At pH 5.0, each of the 48 epigenetically modified sequences showed CD spectra that were similar to the unmodified control sequence (Supplementary Figure S1), indicative of i-motif structure. These results indicate that modification of cytosines to 5mC, 5hmC, 5fC or 5caC does not prevent i-motif formation, in any position. This is consistent with previous research which showed overall i-motif structure was largely unchanged with methylation of cytosine (18), but our work shows this is also true for hydroxymethylation, formylation and carboxylation of cytosine. Previous studies have shown that 5fC alters the structure of a double helix and gives rise to different CD spectra (36). Interestingly, modification with 5fC was not found to alter the structure of i-motif nor significantly change the CD spectra, in the conditions and for the sequence used within this study.

**Figure 2.**
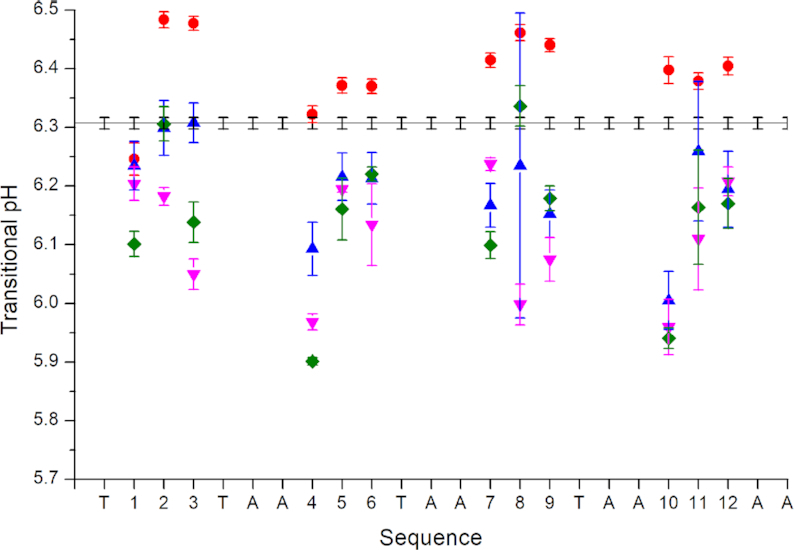
Transitional pH of each of the epigenetically modified (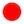
5-methylcytosine; 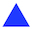
5-hydroxymethylcytosine; 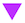
5-formylcytosine; 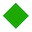
5-carboxylmethylcytosine) hTeloC oligonucleotides compared to the unmodified control sequence (line). Cytosines are numbered from 5′- to 3′-.

The transitional pH was calculated for each of the epigenetically modified sequences by fitting the ellipticity at 288 nm versus pH (Supplementary Figure S1). This biophysical characteristic was found to vary with both the type and site of modification (Figure 2, Supplementary Figures S1, Table S3). Compared to the unmodified control, 5mC modification increased the transitional pH at all but two cytosine positions (Figure 2). At cytosine positions 1 and 4, the transitional pH was reduced and unaltered, respectively. All of the other modifications decreased or left the transitional pH unchanged at each of the cytosine positions. The biggest decreases were consistently observed when cytosine positions 4 and 10 were modified with 5hmC, 5fC or 5caC (Figure 2) i.e. the cytosines contributing to the top cytosine-cytosine base pair (Supplementary Figure S2). A similar decrease in the transitional pH of a 5hmC-modified i-motif had previously been shown for positions 4, 5, 6, 11 and 12 in the sequence from the human telomere (18) and the mid-tract cytosine of a sequence from c-MYC (21). In these studies, adding 5hmC modification instead produced i-motif that were of comparable or greater pH stability with the unmodified control sequence (18,21).

**Figure 3.**
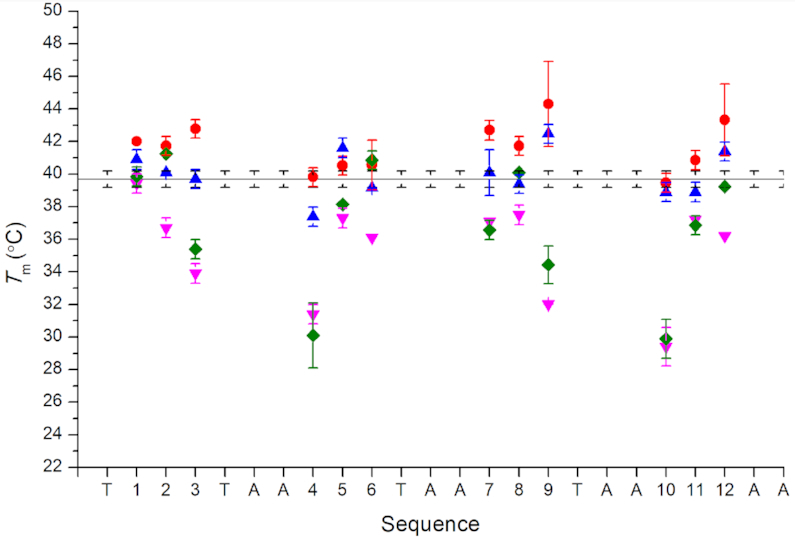
Melting temperature of each of the epigenetically modified (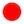
5-methylcytosine; 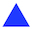
5-hydroxymethylcytosine; 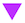
5-formylcytosine; 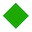
5-carboxylmethylcytosine) hTeloC oligonucleotides compared to the unmodified control sequence (line). Cytosines are numbered from 5′- to 3′-.

The differences in the thermal stabilities of the epigenetically modified i-motifs compared to an unmodified control sequence were dependent on both the type of modification and the cytosine position. However, the TDS for all of the sequences, regardless of which modification was present, were consistent with that of both published i-motif and the unmodified control sequence (positive peak at 240 nm and negative peak at 295 nm, see Supplementary Figure S3) (31). 5mC-modified sequences showed thermal stability that was not significantly different from the control (cytosines 4, 5, 6, 10 and 11) or an improvement ranging from 2.0 to 4.6°C (cytosines 1, 2, 3, 7, 8, 9 and 12) (Figure 3, Supplementary Figure S4, Table S4). The trend shown in the series of 5hmC-modified sequences is less uniform. When cytosines 5, 9 and 12 were modified with 5hmC, melting temperatures were higher than that of the unmodified control. At all other positions, modification with 5hmC appears to have a minimal effect on thermal stability compared to the unmodified control. With the exception of the cytosine at position 4, where a 2.3°C decrease is observed. A similar decrease in melting temperature was also observed when 5hmC was substituted at position 4 in a separate study although opposite effects were observed at positions 5 and 12 (18). This indicates that whilst there are some general trends (Supplementary Figure S5), buffer conditions and slight differences in flanking sequence also contribute at this level, where there are only relatively minor changes in stability, and are within the variability of measuring under different conditions.

Substitution of cytosine with 5fC consistently resulted in unchanged (cytosine 1) or decreased thermal stability of the i-motif (cytosines 2–12). This decrease ranged from 2.2 (cytosine 8) to 10.3°C (cytosine 10) lower than the unmodified control melting temperature (39.7°C). Similarly, when cytosine was substituted for the 5caC modification at positions 3–5, 7 and 9–11, the melting temperature was found to be lower than the unmodified control and this difference ranged from 1.5 to 9.8°C. Of the remaining positions, 5caC modification at cytosines 1, 6 and 12 did not alter the thermal stability of the i-motif (within error). However, at cytosine position 2, a 5caC modification increased the thermal stability of the i-motif by 1.5°C. The thermal stability data largely shows a similar trend to that observed in the transitional pH data; modification at cytosine positions 4 and 10, consistently show the largest decreases no matter which of the 5hmC, 5fC or 5caC modifications is present.

It has been suggested that the pH and thermal destabilization seen with the addition of 5hmC, 5fC and 5caC at various sites could arise due to steric effects of the more bulky modifications (18). Increased thermal and pH stability of sequences with dual 5mC modifications has been attributed to the hydrophobicity of the 5-methyl group (18). Another study that examined how 5mC modification influences the formation of intramolecular i-motif structures used NOESY to resolve the structures of [d(TCC)]_4_ and [d(5mCCT)]_4_ (37). The methylated sequence formed two i-motif tetramers; one was similar to the unmodified i-motif but the conformation of the second was different, possibly to minimize steric hindrance (37). Methylation was previously found to have additive or disruptive effects, depending on the position of the epigenetic modification. For example, hyper-methylation of a whole cytosine tract was found to disrupt i-motif that forms from hTeloC (18). Here, the most significant effect of single modification is observed at positions 4 and 10 (followed by 3 and 9), the positions either side of loops 1 and 3, suggesting an interaction with the loop may have considerable ramifications for the stability of the overall i-motif structure. Certainly, varying loop length affects stability (38), but it is not yet clear whether longer loops may accommodate epigenetic modifications and mitigate these observed decreases in stability. As i-motif structures are hyper-variable and dynamic, it may be challenging to clearly define rules as these will vary greatly depending whether the structure can accommodate changes within extra cytosines in a tract or a loop. With regard to the significant differences in stability for 5caC and 5fC, which in our study were found to weaken i-motif structure, this is consistent with their base pairing properties. Previous studies showed weakened N3 hydrogen bonding by 5fC and 5caC, which reduces their ability to base pair (39). As 5fC and 5caC contain modifications which are electron withdrawing, in i-motif structures these are unable to stabilize the positive charge in the hemi-protonated base pairs, similar to what is observed with halogenated cytosines (23,24).

**Figure 4.**
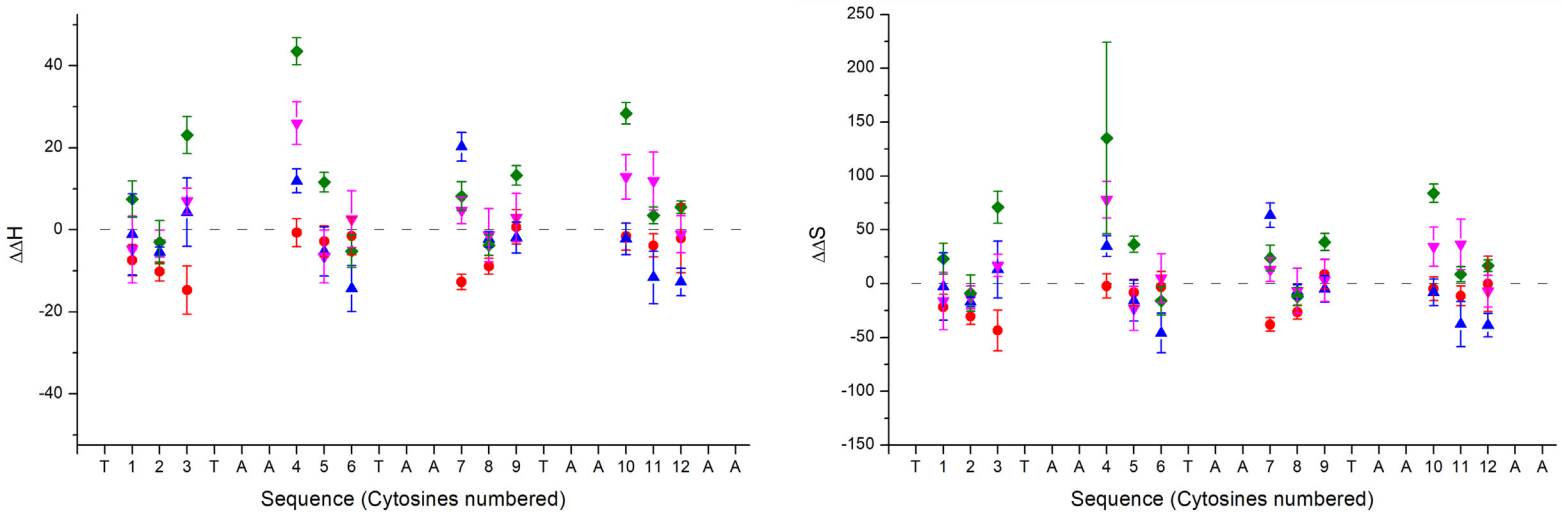
Difference in enthalpy and entropy change calculated for each of the epigenetically modified (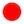
5-methylcytosine; 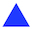
5-hydroxymethylcytosine; 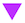
5-formylcytosine; 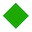
5-carboxylmethylcytosine) hTeloC oligonucleotides compared to the unmodified control sequence (line). Cytosines are numbered from 5′- to 3′-

The entropy and enthalpy values were also determined from the UV melts of each of the epigenetically modified sequences. These thermodynamic values describe the temperature dependence of the equilibrium constant for the melting and annealing of the i-motif (29). The more negative the change in these values, compared to the unmodified oligonucleotide, the more favorable the process (Figure 4 and Supplementary Figure S6; Table S5). These values also reflected the trend seen in the pH and thermal stability data; i.e. the most thermally unstable modified sequences, had the least negative changes in enthalpy and entropy.

The fact that the bases either side of the middle loop are relatively unaffected by modification, whereas those either side of loops 1 and 3 are most affected by modification is in-line with other studies which show the middle loop is able to tolerate changes which would otherwise be destabilizing. For example, the middle loop has previously been shown to accommodate a longer sequence with minimal disruption to the stability whereas shorter loops are typically more favorable (38). Moreover, the type of sequences within the loops and lengths of loops in the first and third loop regions have previously been shown to contribute to i-motif stability (40). The sensitivity to modification of bases that flank a loop has been highlighted before, both in terms of pH sensitivity and temperature. When a similar sequence from the human telomere was modified with a guanylurea at the third cytosine in the first tract, the transitional pH (pH_T_ 5.8) and melting temperature (*T*_m_ = 30.2°C) were reduced compared to the control (pH_T_ 6.1; *T*_m_ = 46.4°C) (41). Removing a cytosine and replacing it with thymine has also been used to study i-motif stability as part of a mutational analysis (42). In this example, the exchange of a cytosine for a thymine nearest a loop resulted in a decrease in melting temperature from 49.5°C to 45.5°C (43). A similar effect was observed when the cytosine-rich sequence upstream of the bcl-2 promoter site was subjected to thymine substitution (42). In this instance, the cytosines at either end of two tracts were altered to thymines; this resulted in a decrease in melting temperature of between ∼5 and 10°C (42). To complement this previous work and give an indication of the effects of substitution with thymine in the sequence in this study, we also performed a thymine screen, substituting individual cytosines with thymines (Supplementary Tables S1, S6 and S7; Figures S7–S13). We found that, although substitution of cytosine to thymine closest to the loops did show a decrease in melting temperature, the largest change was actually observed with substitution of cytosines in the middle of the tract; i.e. Cytosines 2, 5, 8 and 11 were affected by this substitution the most (Supplementary Figures S10 and S13). This indicates that the pattern observed with the epigenetic modifications is not consistent with the cytosines simply no longer base pairing. In fact, the decrease in melting temperature was larger for the modified cytosines (5fC and 5caC) than for substitution with thymine. For example, 5fC showed a change in melting temperature of -8.3 and -10.3°C at positions 4 and 10 respectively whereas substitution with thymine gave changes of only -7.1 and -5.7°C. These results also provide further information for those who are designing sequences to fold into i-motif, that single mutations significantly reduce the stability, but i-motif can still form. Together, this work contributes towards better predictions for i-motif folding and stability, allowing fine tuning of i-motif switches (44–46) and applications in DNA nanotechnology (47–49).

The effect of epigenetic modification has a bearing on many cytosine-rich regions of DNA. A common site for modification is at CpG sites, where methylation is usually associated with gene silencing (50). Epigenetic modification of the human telomeric sequence has been associated with cancer genotypes (33). Given the position of cytosine modification is critical to stability of i-motif structure, we were interested in surveying epigenetic modification in known i-motif forming sequences across the human genome. We selected 44 i-motif forming sequences from the literature, which had a wide range of stabilities and positions in the genome. Using the MCF7 (27) and MCF10A (28) methylomes, we identified the 5mC sites on these sequences to determine where i-motif cytosines are modified in a breast cancer cell line (MCF7) vs a non-tumorigenic mammary epithelial cell line (MCF10A), see Supplementary Table S8. Out of the i-motif forming sequences, 12 (27%) of the i-motifs contained at least one methylated cytosine. Amongst these 12 sequences, the majority, 10 (83%) were differentially methylated in MCF7 vs MCF10A, indicative of the different methylation variants in cancerous compared to non-cancerous cells. Our biophysical study found that the most significant effect of single modification is shown at positions 4 and 10 (followed by 3 and 9), i.e. the positions either side of loops 1 and 3. Out of the methylated i-motifs we studied, eight (67%) were methylated in at least one of these regions: NFATC1, SHANK3b, HOCD10, MSMO1, PLCB2, WNT7A, C9ORF72, SOX1. This may indicate that the presence and position of methylation in C-rich sequences has a purpose which could be related to stability of i-motif DNA secondary structure and biological function.

Finally, we were interested in whether there was a difference in the likelihood of epigenetic modification, depending on the relative stability of the i-motif structure. i-Motif structures were previously thought to only be stable in acidic conditions, but more recently there have been examples of particular sequences which are stable at neutral pH (26,51). We split the i-motif forming sequences into traditional acidic i-motif forming sequences, stable under acidic conditions, and neutral i-motif forming sequences, defined as those which had a transitional pH > pH 7.0 ± 0.2. Although the numbers are relatively small, of the methylated i-motif forming sequences, significantly more were found to be in neutral i-motifs (*n* = 10, 77%) compared to only 3 (23%) in traditional acidic i-motifs (comparison of proportions, *P* = 0.0995). To complement the *in silico* study, we selected two other sequences from the human genome and examined the effects of the native epigenetic modification (Supplementary Figures S14–S21 and Tables S9–S11). Sequences from the promoter regions of MSMO1 [5′-CCC-CCG-CCC-CCG-CCC-CCG-CCC-CC-3′] and PCLB2 [5′-CCC-CCG-CCT-CTT-CTG-GAG-GCC-CCC-GCC-CCC-ACC-CCC-3′] were chosen as we had previously characterized their properties (26) and both had native methylation next to loops, in line with our hypothesis that these regions are sensitive to modification. MSMO1 has one position modified, whereas PCLB2 has two; the 15th cytosine in the MSMO1 sequence was modified and for PLCB2 both the 5th and the 14th cytosines were modified. In these cases it was also found that the 5fC analogues of MSMO1 and PCLB2 were less stable (*T*_m_ = 57.4 and 53.9°C respectively) than the unmodified sequences (*T*_m_ = 60.8 and 56.6°C respectively, Supplementary Table S9). This effect was not replicated with the 5caC analogues however. The sequences of MSMO1 and PCLB2 are longer compared to hTeloC, with C-tracts of five cytosines whereas hTeloC has C-tracts of three cytosines. This does enable more opportunity for adjustment and slight changes in structure to accommodate modifications. The C-rich cores of these i-motifs are composed of more C–C base pairs compared to hTeloC; and as such are more stable in the unmodified sequence. It is worth noting that even with i-motifs that are naturally more stable, and can have several degenerate folding possibilities to accommodate a modification, the 5fC analogues still disrupt i-motif structure. These results are very interesting, given the recent discovery of the potential biological functions of 5fC (14,15).

Here we have described how both the position and type of epigenetic modification of cytosine affects i-motif stability. We have shown that cytosines either side of loops 1 and 3 are most sensitive to epigenetic modification. Using known i-motif forming sequences from the literature, we can see these critical positions are where epigenetic modification occurs in the human genome. Moreover, i-motifs which are stable at neutral pH are more likely to be methylated than their acidic counterparts. Understanding how these epigenetic modifications impact upon i-motif structural stability will help us to interpret and influence their biological function. Our findings also provide means to fine-tune the pH and temperature stability of i-motif, increasing their utility in nanotechnological applications.

## Supplementary Material

gkz1082_Supplemental_File
