# Supplementary material for: Epigenetic modification of cytosines fine tunes the stability of i-motif DNA
Source: Nucleic Acids Res. 2019 Nov 28;48(1):55–62. doi: 10.1093/nar/gkz1082 (PMC6943138; doi:10.1093/nar/gkz1082)
Supplement: gkz1082_Supplemental_File [file gkz1082_supplemental_file.pdf]

Epigenetic Modification of Cytosines Fine Tunes the Stability of i-Motif DNA

Elisé P. Wright,[1,§] Mahmoud A. S. Abdelhamid,[1,§] Michelle O. Ehiabor,[1] Melanie C. Grigg,[2] Kelly Irving, [2] Nicole Smith,[2] and Zoë A. E. Waller[1,3]*

[1] School of Pharmacy, University of East Anglia, Norwich Research Park, Norwich, NR4 7TJ, UK.

[2] School of Molecular Sciences, University of Western Australia, 35 Stirling Hwy, Crawley WA 6009, Australia.

[3] Centre for Molecular and Structural Biochemistry, University of East Anglia, Norwich Research Park, Norwich, NR4 7TJ, UK.

[§] These authors contributed equally to this work.

SUPPORTING INFORMATION

1) ADDITIONAL OLIGONUCLEOTIDES S2

2) CIRCULAR DICHROISM OF CYTOSINE MODIFICATIONS S3

3) THERMAL DIFFERENCE SPECTRA OF CYTOSINE MODIFICATIONS S21

4) UV SPECTROSCOPY OF CYTOSINE MODIFICATIONS S22

5) CIRCULAR DICHROISM FOR THE THYMINE SCREEN S34

6) UV SPECTROSCOPY FOR THE THYMINE SCREEN S39

7) I-MOTIFS AND METHYLATION PROFILES S44

8) UV SPECTROSCOPY OF MSMO1 AND PLCB2 S49

9) CIRCULAR DICHROISM MSMO1 AND PLCB2 S53

ADDITIONAL OLIGONUCLEOTIDES

Table S1. ODNs used to study cytosine to thymine mutation of hTeloC sequence and key describing mutation location.

Sequence Name	Sequence (5'-3')
hTeloCT1	T*T**C-CTA-ACC-CTA-ACC-CTA-ACC-CAA
hTeloCT2	TC*T**-CTA-ACC-CTA-ACC-CTA-ACC-CAA
hTeloCT3	TCC-*T**TA-ACC-CTA-ACC-CTA-ACC-CAA
hTeloCT4	TCC-CTA-A*T**C-CTA-ACC-CTA-ACC-CAA
hTeloCT5	TCC-CTA-AC*T**-CTA-ACC-CTA-ACC-CAA
hTeloCT6	TCC-CTA-ACC-*T**TA-ACC-CTA-ACC-CAA
hTeloCT7	TCC-CTA-ACC-CTA-A*T**C-CTA-ACC-CAA
hTeloCT8	TCC-CTA-ACC-CTA-AC*T**-CTA-ACC-CAA
hTeloCT9	TCC-CTA-ACC-CTA-ACC-*T**TA-ACC-CAA
hTeloCT10	TCC-CTA-ACC-CTA-ACC-CTA-A*T**C-CAA
hTeloCT11	TCC-CTA-ACC-CTA-ACC-CTA-AC*T**-CAA
hTeloCT12	TCC-CTA-ACC-CTA-ACC-CTA-ACC-*T**AA

*T** indicates position of mutated cytosine

Table S2. Unmodified and epigenetically modified MSMO1 and PLCB2 sequences and key describing modification location.

Sequence Name	Sequence (5'-3')
MSMO1-5xC	CCC-CCG-CCC-CCG-CCC-C*X**G-CCC-CC
PLCB2-5xC	CCC-C*X**G-CCT-CTT-CTG-GAG-GCC-CC*X**-GCC-CCC-ACC-CCC

*X** indicates position of modified cytosine(s)

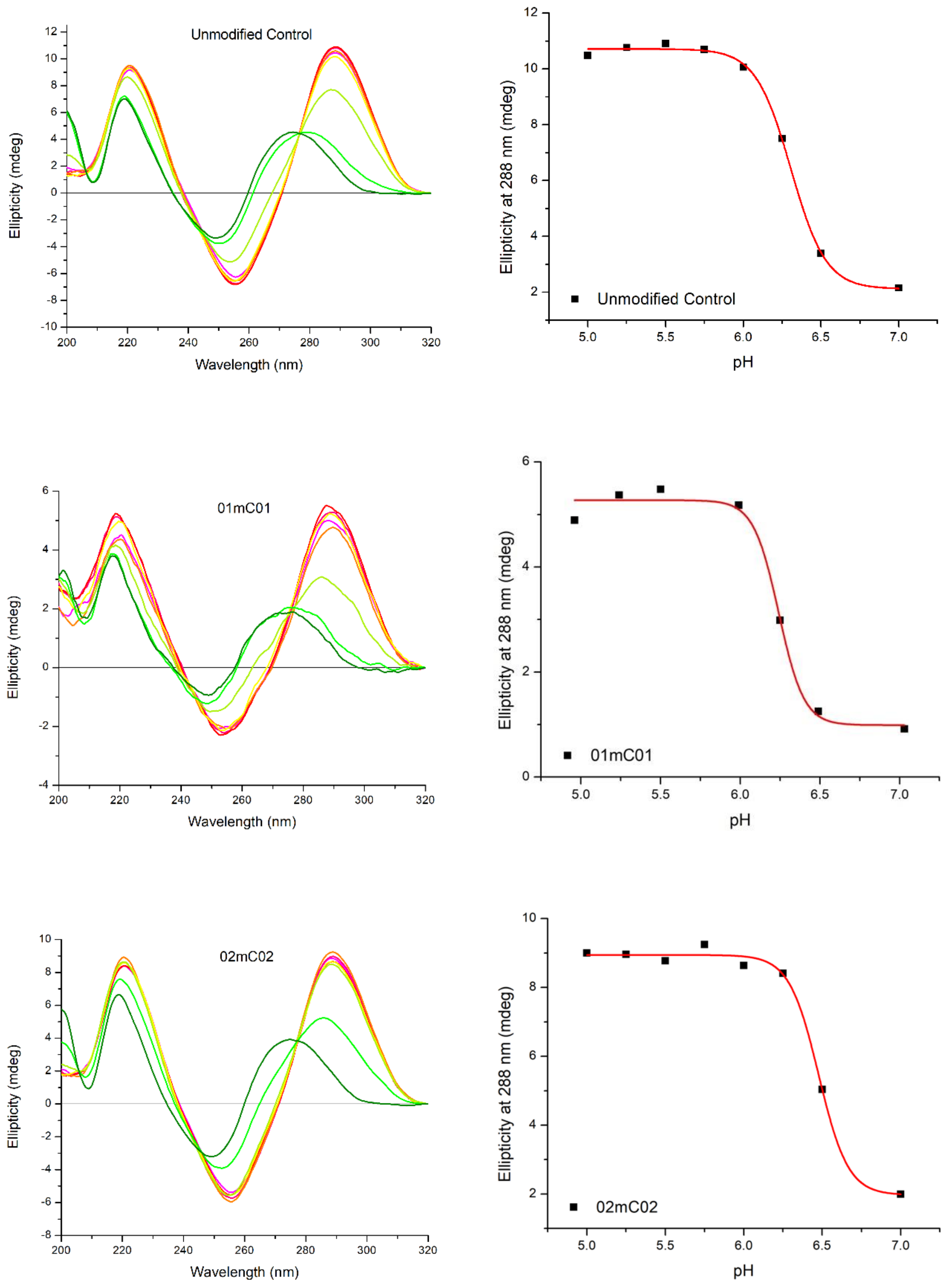

Figure S1A. CD spectra for epigenetically modified hTeloC. All oligonucleotides were diluted to a final concentration of 10 μM in 10 mM sodium cacodylate with 100 mM sodium chloride at the indicated pH ■pH 5.0; ■pH 5.25; ■pH 5.5; ■pH 5.75; ■pH 6.0; ■pH 6.25; ■pH 6.5; and ■pH 7.0.

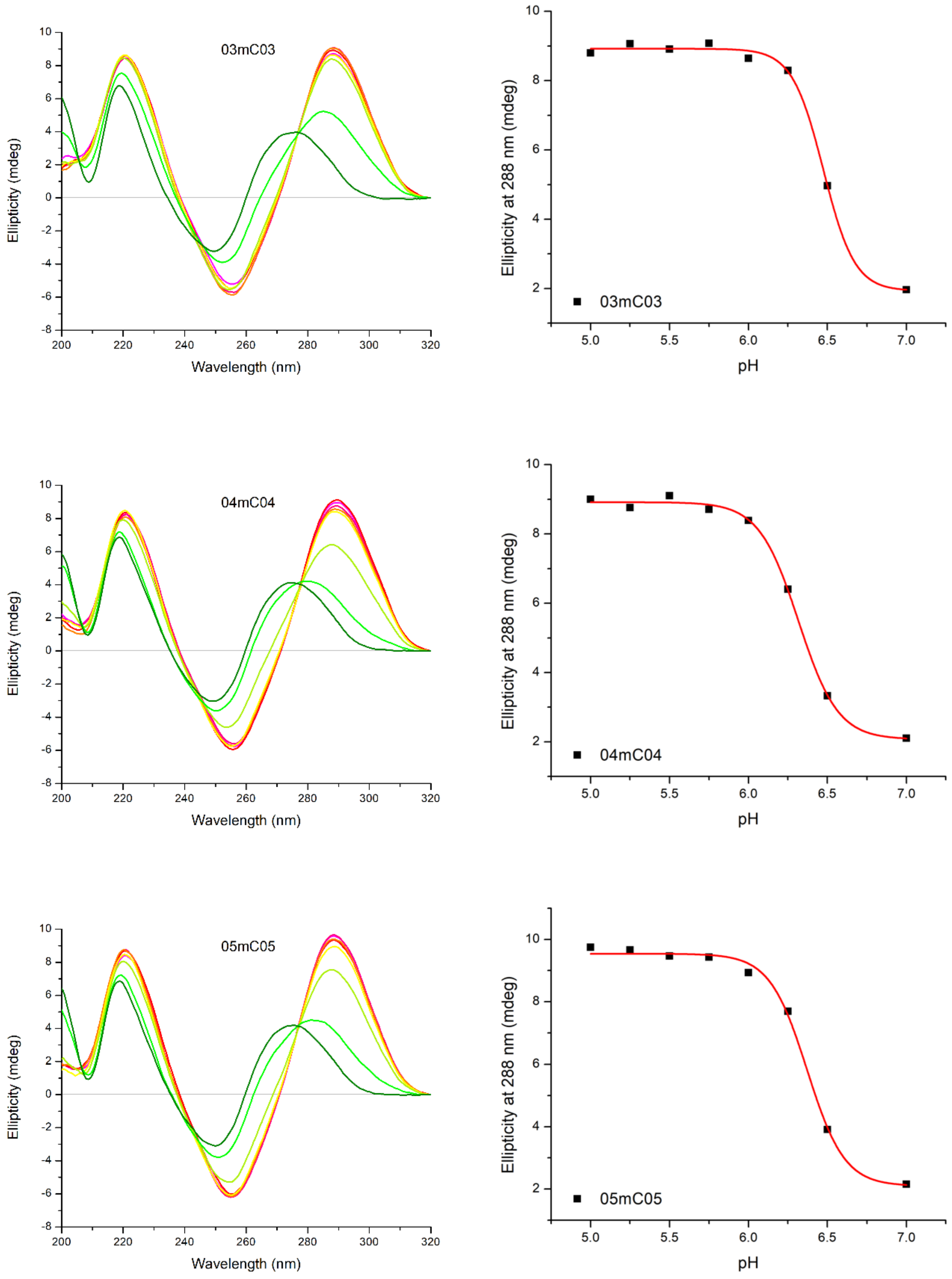

Figure S1B. CD spectra for epigenetically modified hTeloC. All oligonucleotides were diluted to a final concentration of 10 µM in 10 mM sodium cacodylate with 100 mM sodium chloride at the indicated pH ■pH 5.0; ■pH 5.25; ■pH 5.5; ■pH 5.75; ■pH 6.0; ■pH 6.25; ■pH 6.5; and ■pH 7.0.

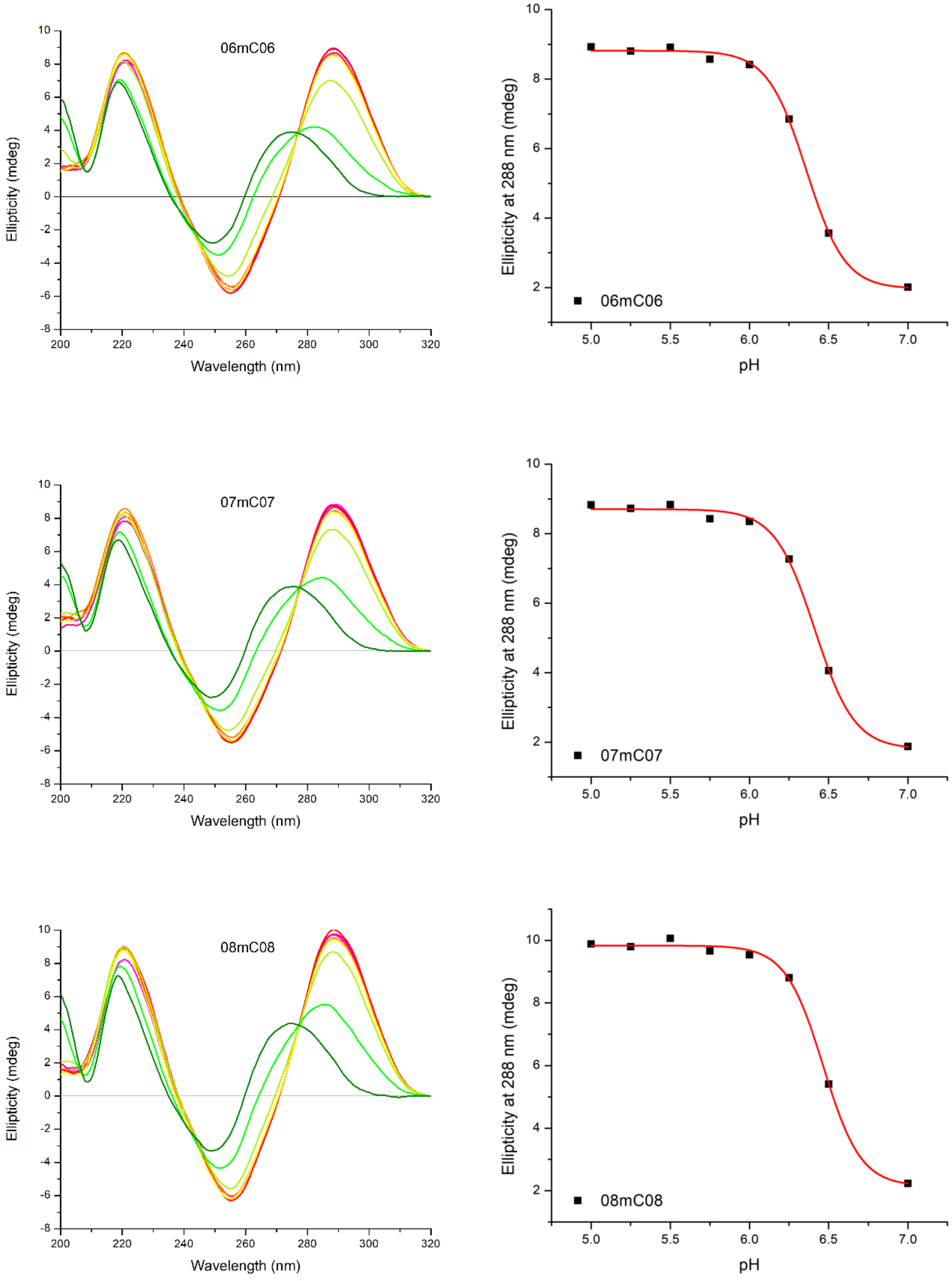

Figure S1C. CD spectra for epigenetically modified hTeloC. All oligonucleotides were diluted to a final concentration of 10 µM in 10 mM sodium cacodylate with 100 mM sodium chloride at the indicated pH ■pH 5.0; ■pH 5.25; ■pH 5.5; ■pH 5.75; ■pH 6.0; ■pH 6.25; ■pH 6.5; and ■pH 7.0.

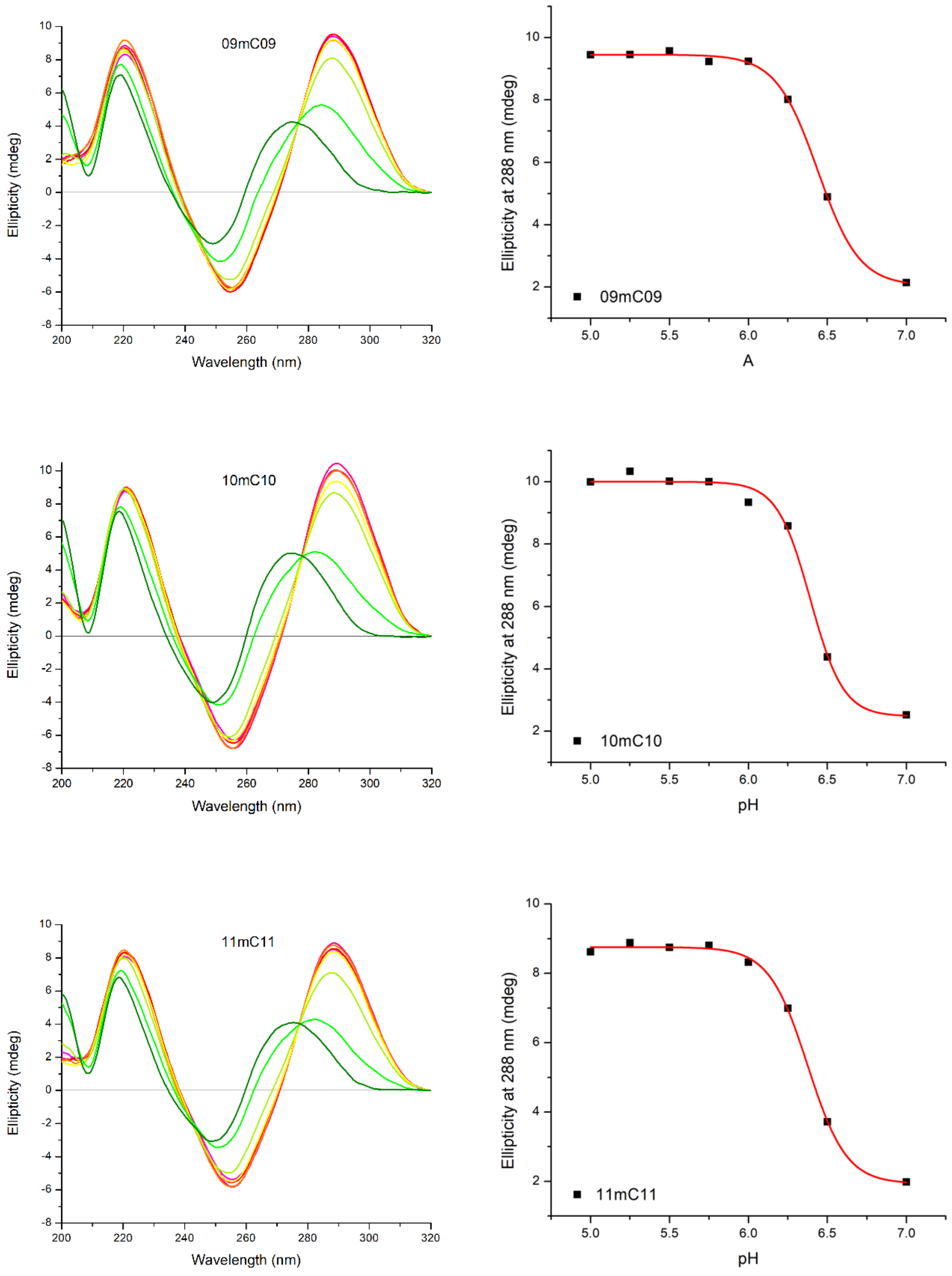

Figure S1D. CD spectra for epigenetically modified hTeloC. All oligonucleotides were diluted to a final concentration of 10 μM in 10 mM sodium cacodylate with 100 mM sodium chloride at the indicated pH ■pH 5.0; ■pH 5.25; ■pH 5.5; ■pH 5.75; ■pH 6.0; ■pH 6.25; ■pH 6.5; and ■pH 7.0.

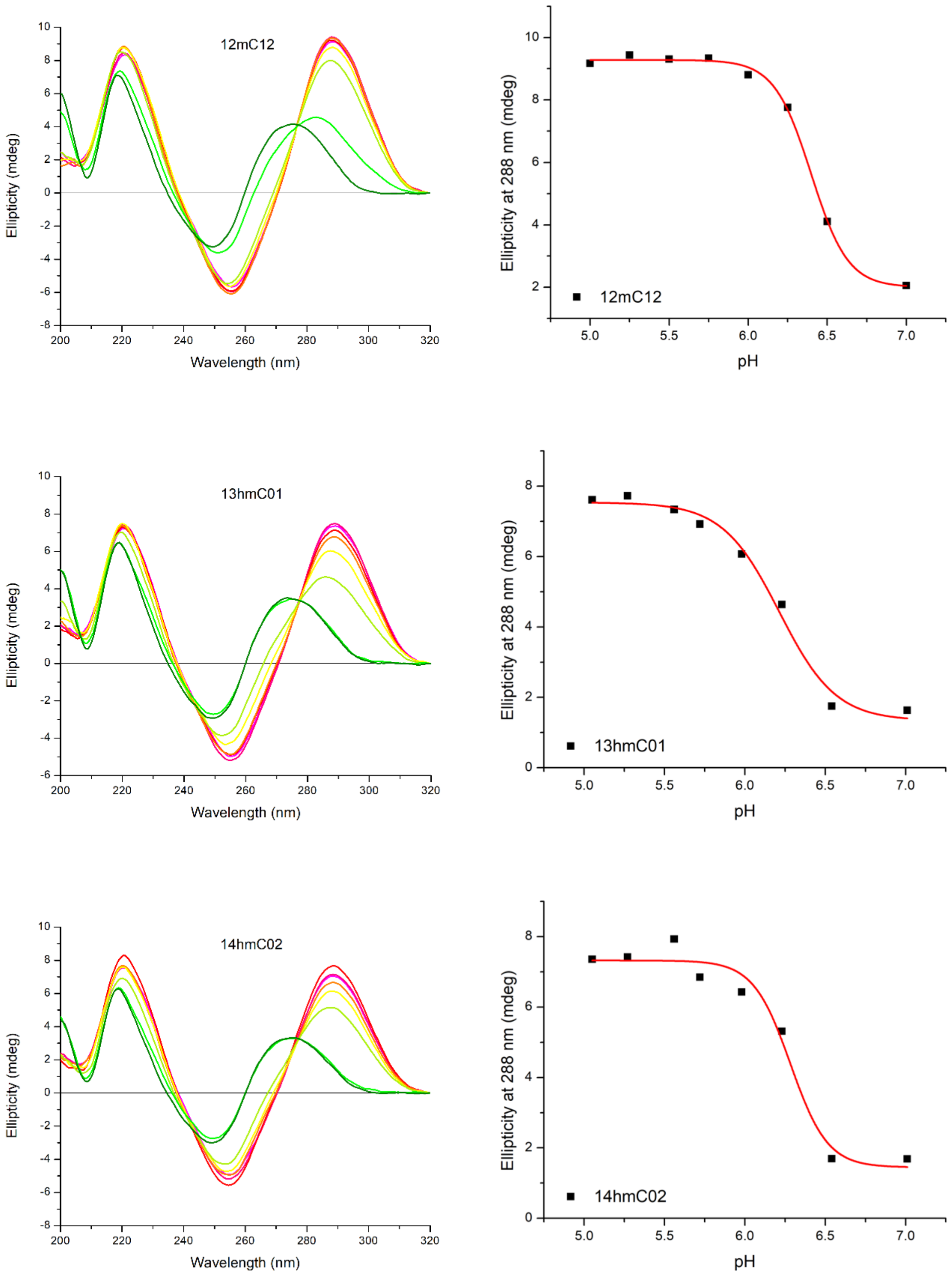

Figure S1E. CD spectra for epigenetically modified hTeloC. All oligonucleotides were diluted to a final concentration of 10 μM in 10 mM sodium cacodylate with 100 mM sodium chloride at the indicated pH ■pH 5.0; ■pH 5.25; ■pH 5.5; ■pH 5.75; ■pH 6.0; ■pH 6.25; ■pH 6.5; and ■pH 7.0.

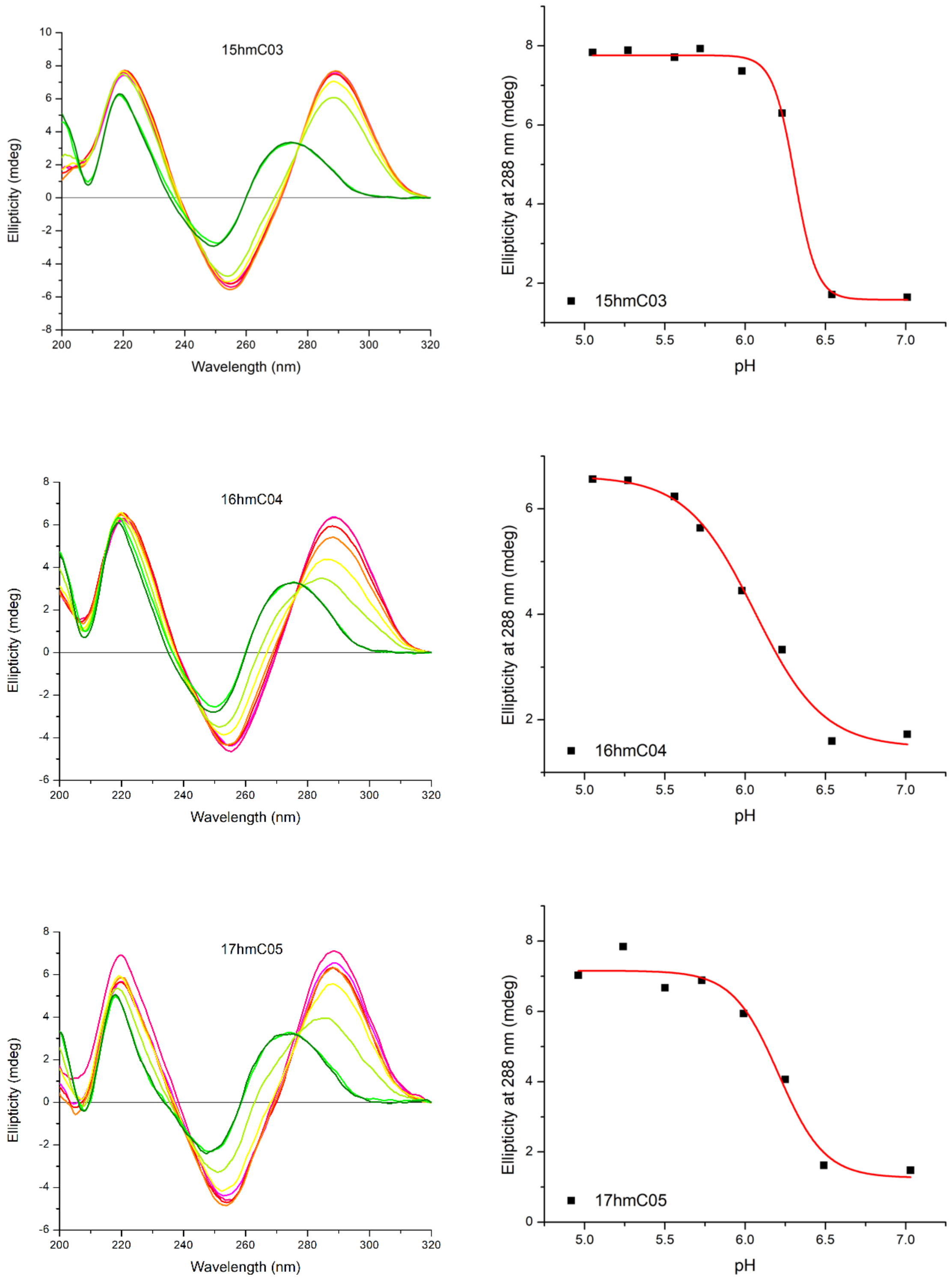

Figure S1F. CD spectra for epigenetically modified hTeloC. All oligonucleotides were diluted to a final concentration of 10 μM in 10 mM sodium cacodylate with 100 mM sodium chloride at the indicated pH ■pH 5.0; ■pH 5.25; ■pH 5.5; ■pH 5.75; ■pH 6.0; ■pH 6.25; ■pH 6.5; and ■pH 7.0.

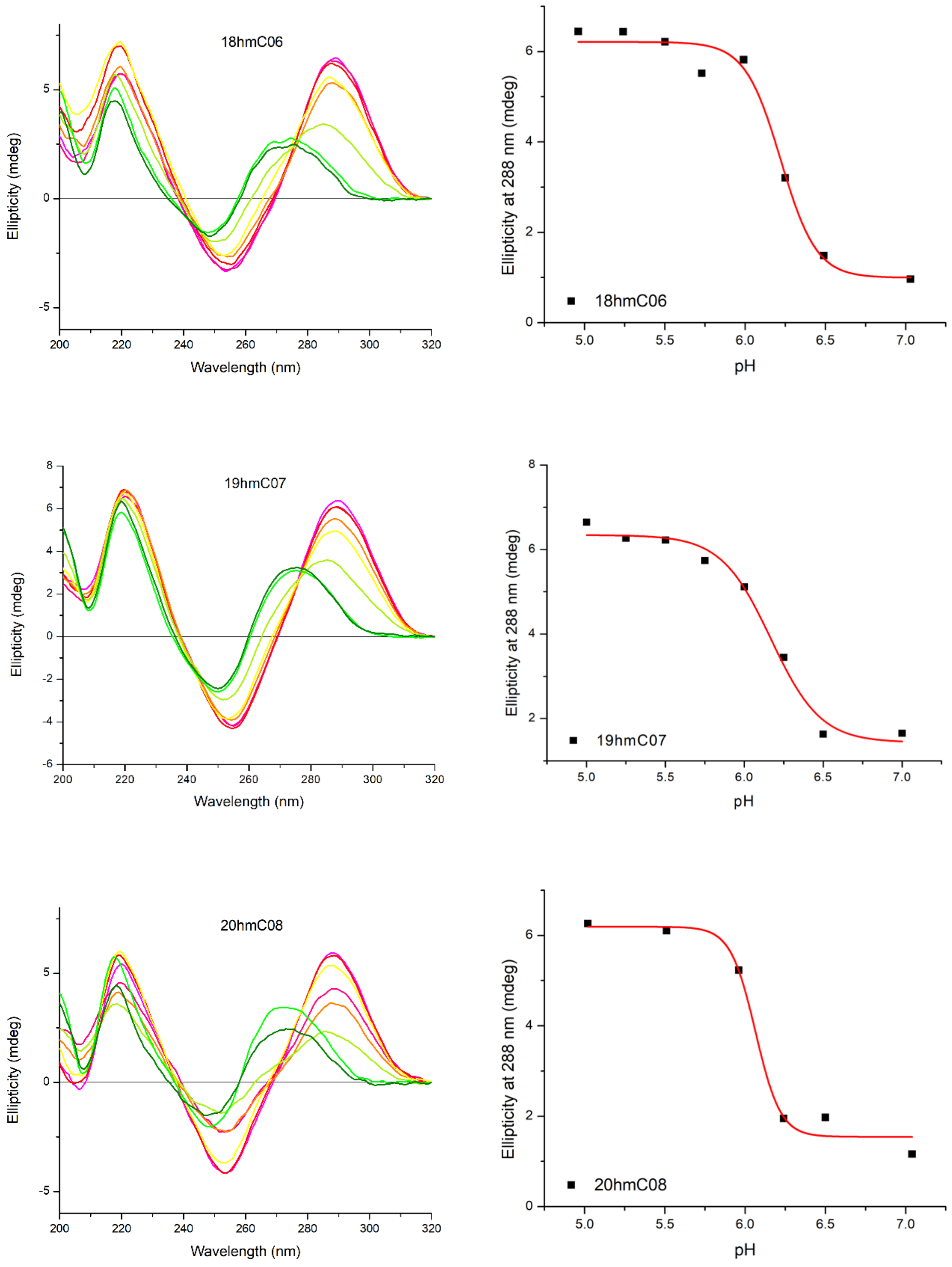

Figure S1G. CD spectra for epigenetically modified hTeloC. All oligonucleotides were diluted to a final concentration of 10 μM in 10 mM sodium cacodylate with 100 mM sodium chloride at the indicated pH ■pH 5.0; ■pH 5.25; ■pH 5.5; ■pH 5.75; ■pH 6.0; ■pH 6.25; ■pH 6.5; and ■pH 7.0.

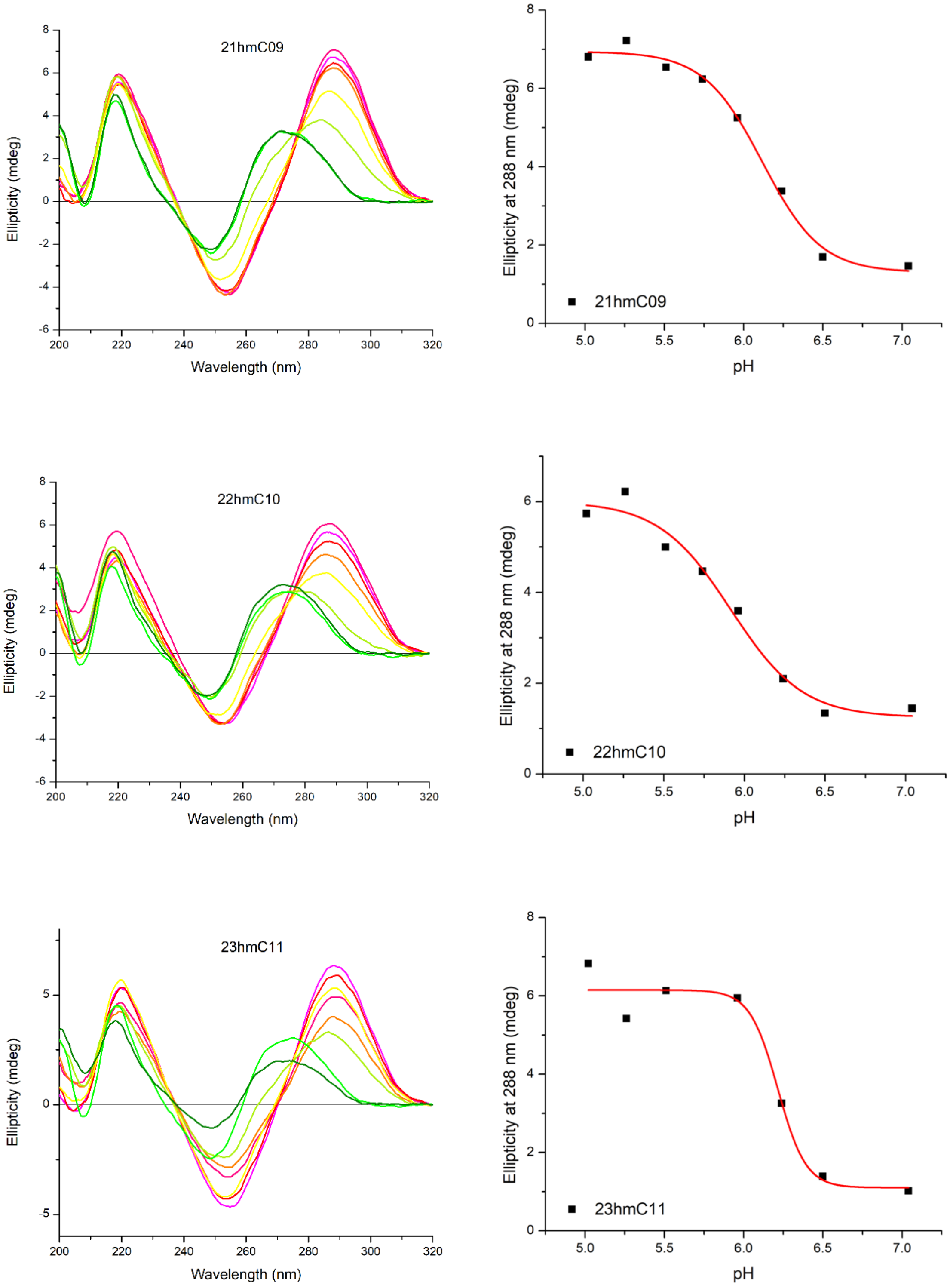

Figure S1H. CD spectra for epigenetically modified hTeloC. All oligonucleotides were diluted to a final concentration of 10 μM in 10 mM sodium cacodylate with 100 mM sodium chloride at the indicated pH pH 5.0; pH 5.25; pH 5.5; pH 5.75; pH 6.0; pH 6.25; pH 6.5; and pH 7.0.

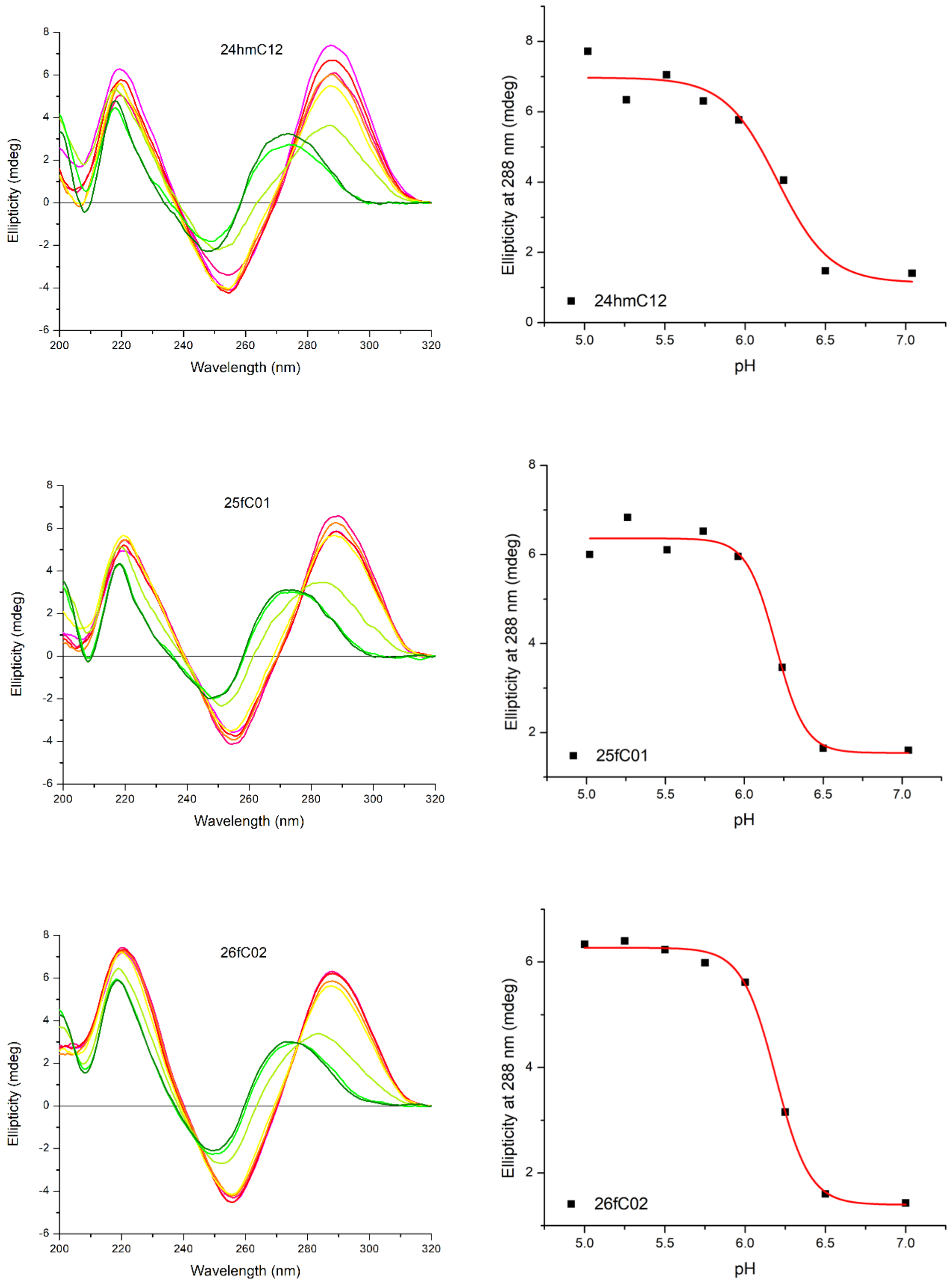

Figure S1I. CD spectra for epigenetically modified hTeloC. All oligonucleotides were diluted to a final concentration of 10 μM in 10 mM sodium cacodylate with 100 mM sodium chloride at the indicated pH ■pH 5.0; ■pH 5.25; ■pH 5.5; ■pH 5.75; ■pH 6.0; ■pH 6.25; ■pH 6.5; and ■pH 7.0.

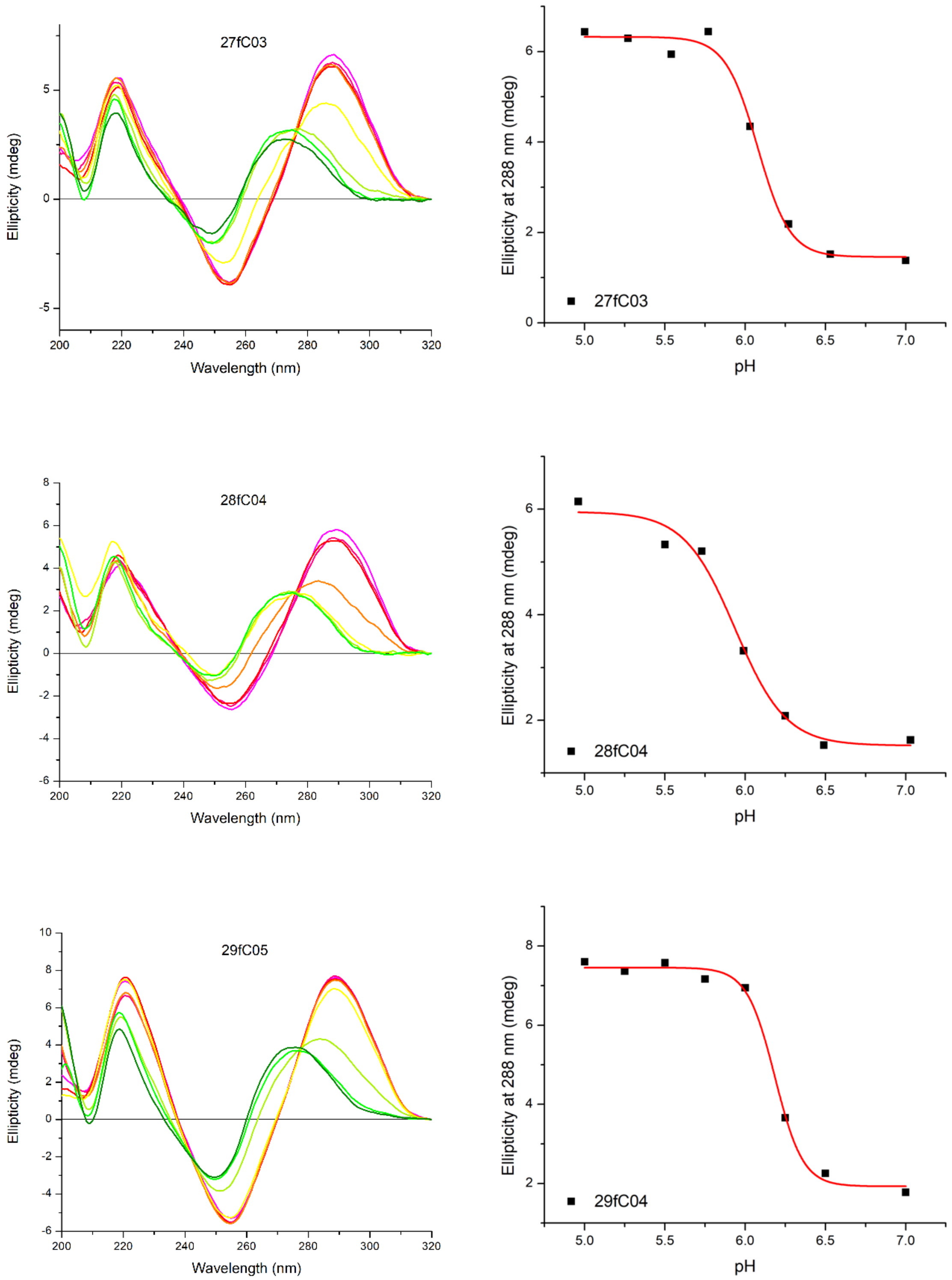

Figure S1J. CD spectra for epigenetically modified hTeloC. All oligonucleotides were diluted to a final concentration of 10 μM in 10 mM sodium cacodylate with 100 mM sodium chloride at the indicated pH ■pH 5.0; ■pH 5.25; ■pH 5.5; ■pH 5.75; ■pH 6.0; ■pH 6.25; ■pH 6.5; and ■pH 7.0.

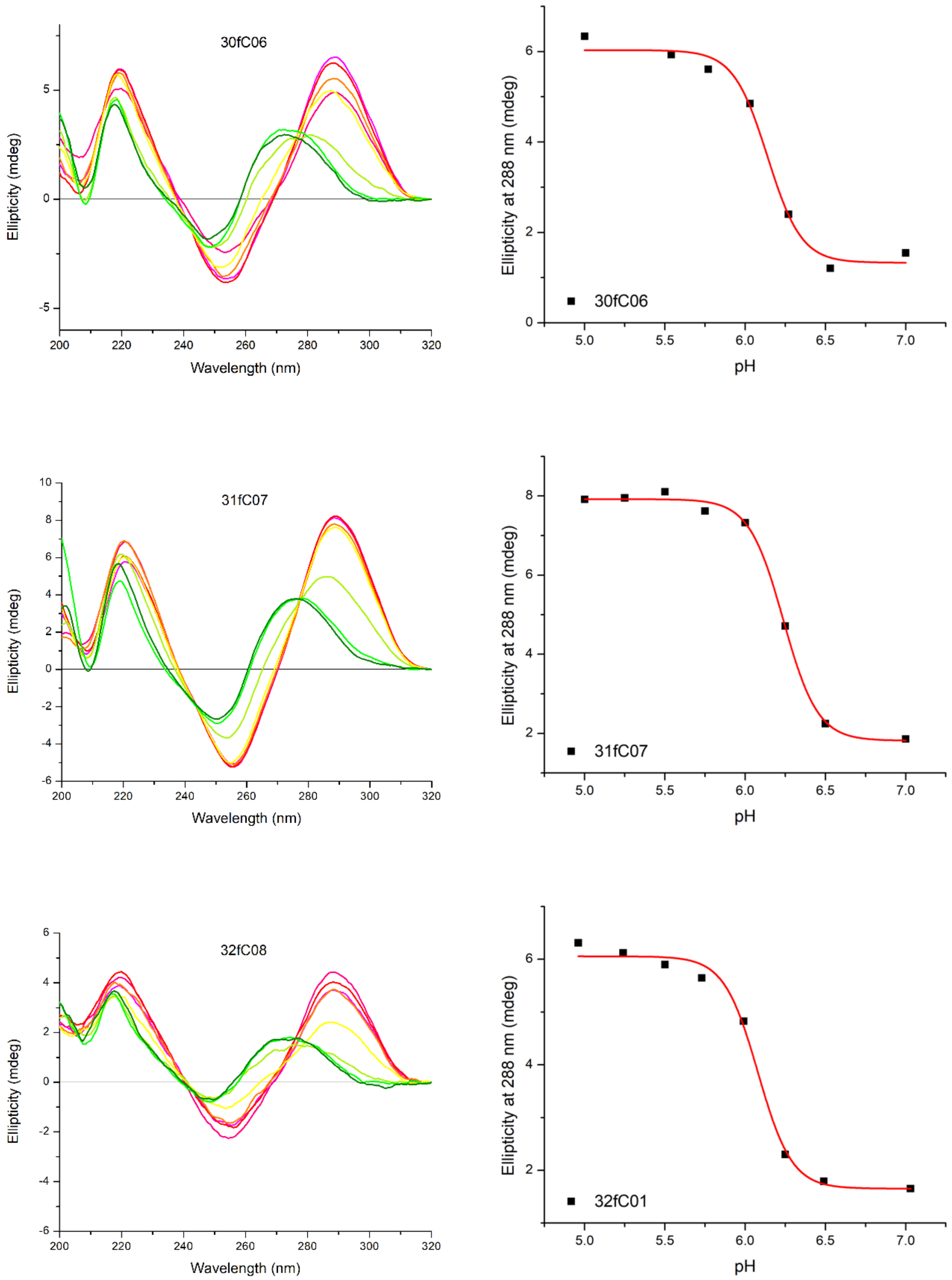

Figure S1K. CD spectra for epigenetically modified hTeloC. All oligonucleotides were diluted to a final concentration of 10 μM in 10 mM sodium cacodylate with 100 mM sodium chloride at the indicated pH ■pH 5.0; ■pH 5.25; ■pH 5.5; ■pH 5.75; ■pH 6.0; ■pH 6.25; ■pH 6.5; and ■pH 7.0.

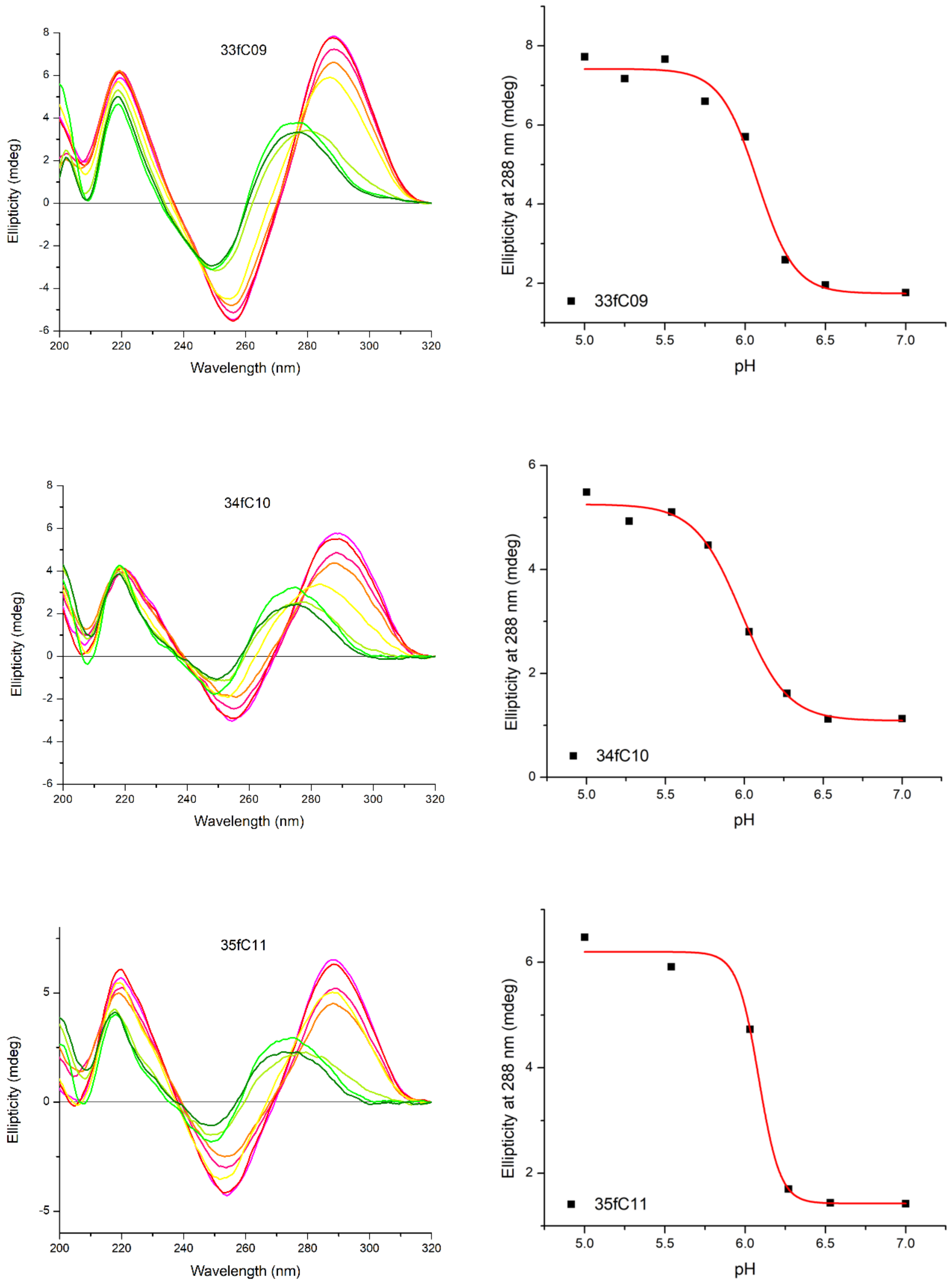

Figure S1L. CD spectra for epigenetically modified hTeloC. All oligonucleotides were diluted to a final concentration of 10 μM in 10 mM sodium cacodylate with 100 mM sodium chloride at the indicated pH ■pH 5.0; ■pH 5.25; ■pH 5.5; ■pH 5.75; ■pH 6.0; ■pH 6.25; ■pH 6.5; and ■pH 7.0

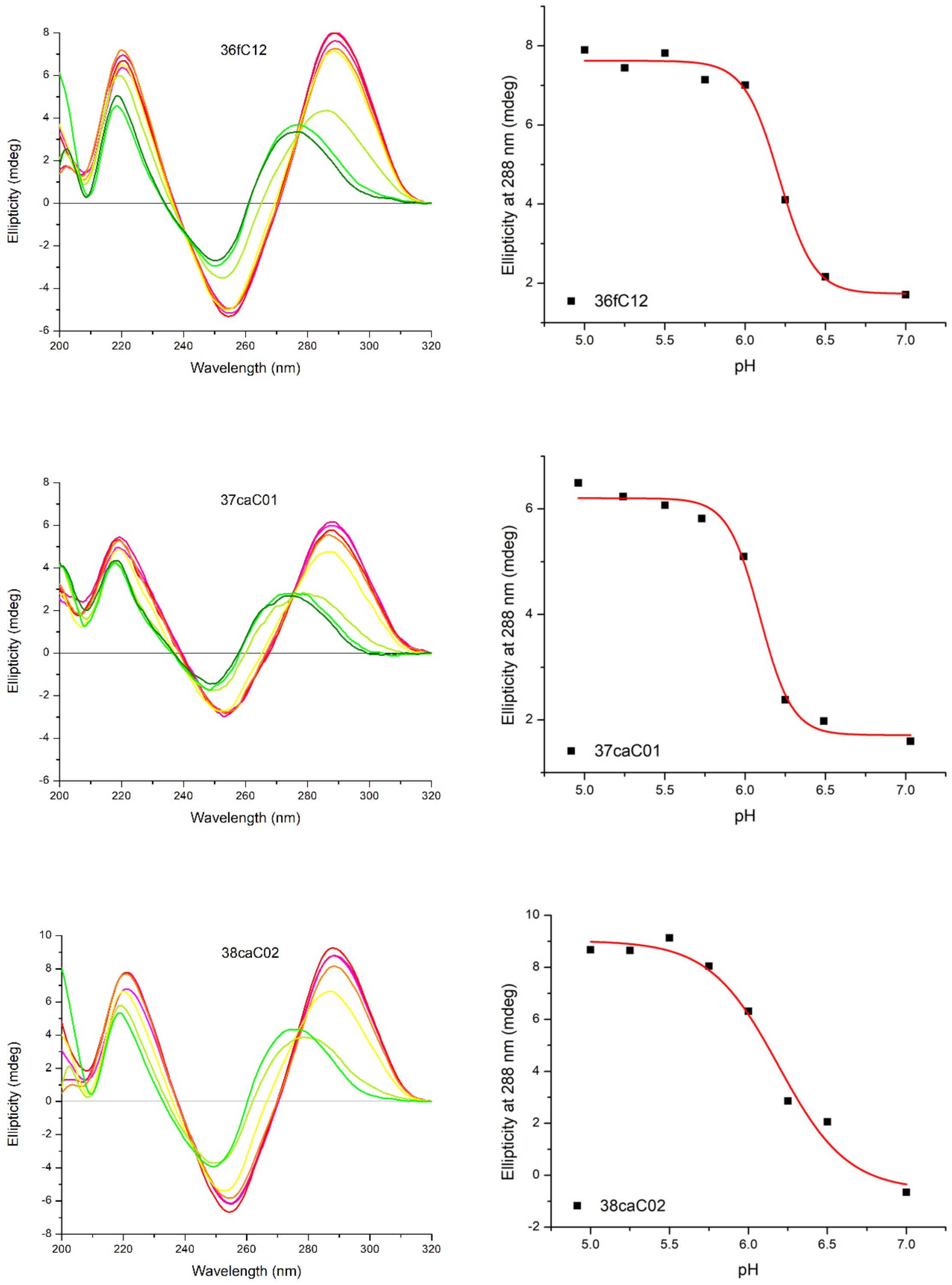

Figure S1M. CD spectra for epigenetically modified hTeloC. All oligonucleotides were diluted to a final concentration of 10 μM in 10 mM sodium cacodylate with 100 mM sodium chloride at the indicated pH ■pH 5.0; ■pH 5.25; ■pH 5.5; ■pH 5.75; ■pH 6.0; ■pH 6.25; ■pH 6.5; and ■pH 7.0.

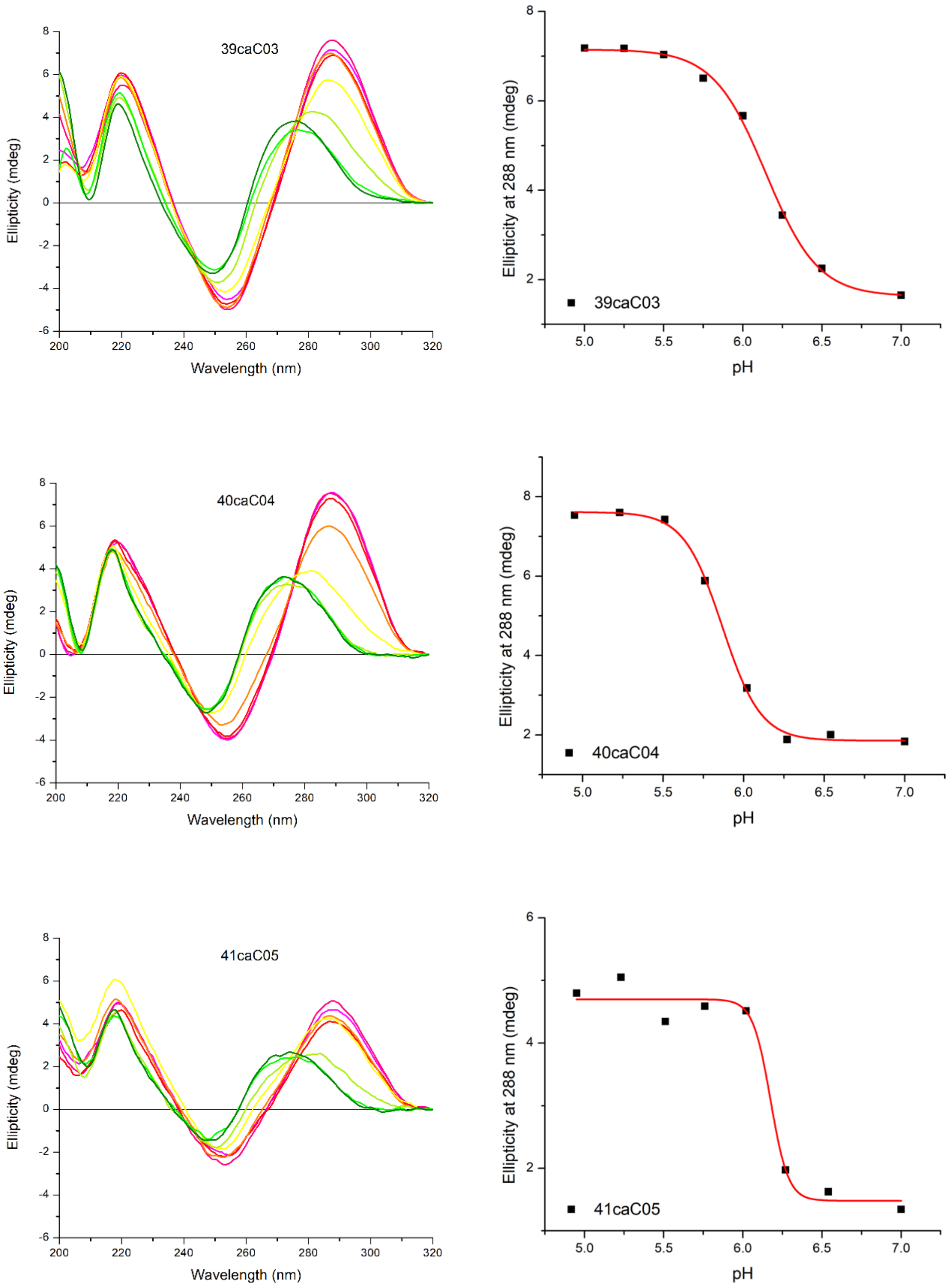

Figure S1N. CD spectra for epigenetically modified hTeloC. All oligonucleotides were diluted to a final concentration of 10 µM in 10 mM sodium cacodylate with 100 mM sodium chloride at the indicated pH ■pH 5.0; ■pH 5.25; ■pH 5.5; ■pH 5.75; ■pH 6.0; ■pH 6.25; ■pH 6.5; and ■pH 7.0.

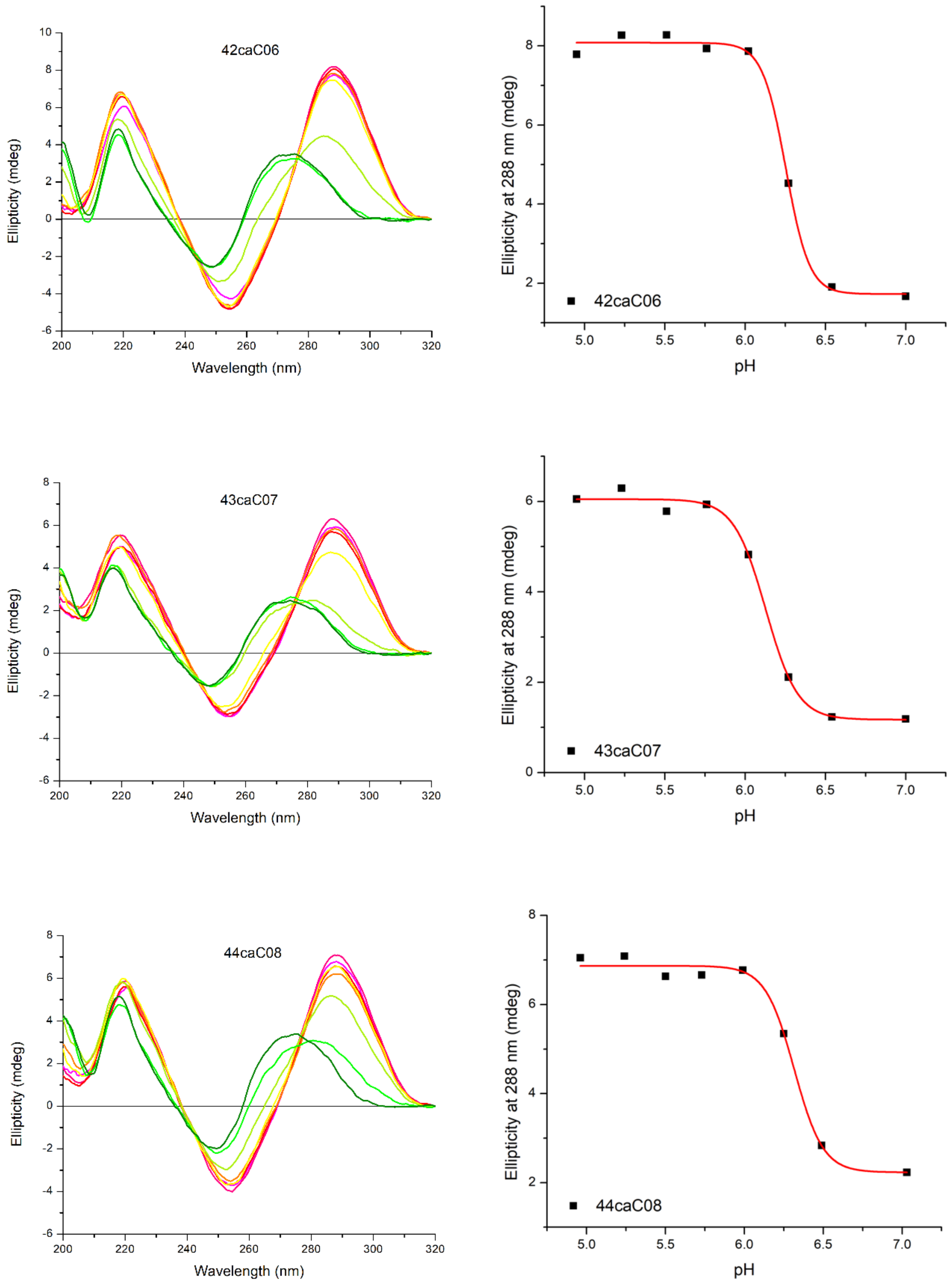

Figure S10. CD spectra for epigenetically modified hTeloC. All oligonucleotides were diluted to a final concentration of 10 µM in 10 mM sodium cacodylate with 100 mM sodium chloride at the indicated pH ■pH 5.0; ■pH 5.25; ■pH 5.5; ■pH 5.75; ■pH 6.0; ■pH 6.25; ■pH 6.5; and ■pH 7.0.

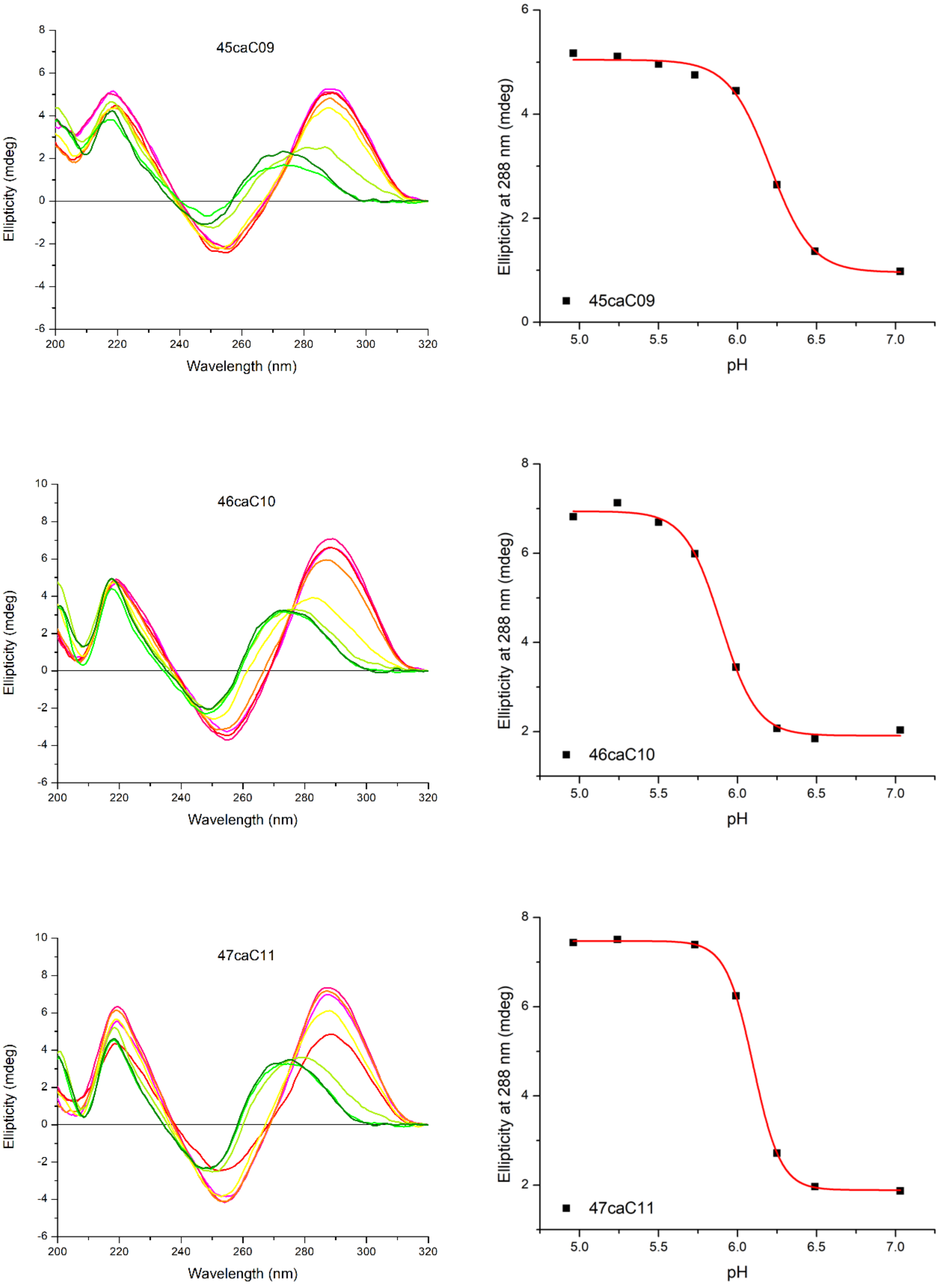

Figure S1P. CD spectra for epigenetically modified hTeloC. All oligonucleotides were diluted to a final concentration of 10 μM in 10 mM sodium cacodylate with 100 mM sodium chloride at the indicated pH ■pH 5.0; ■pH 5.25; ■pH 5.5; ■pH 5.75; ■pH 6.0; ■pH 6.25; ■pH 6.5; and ■pH 7.0.

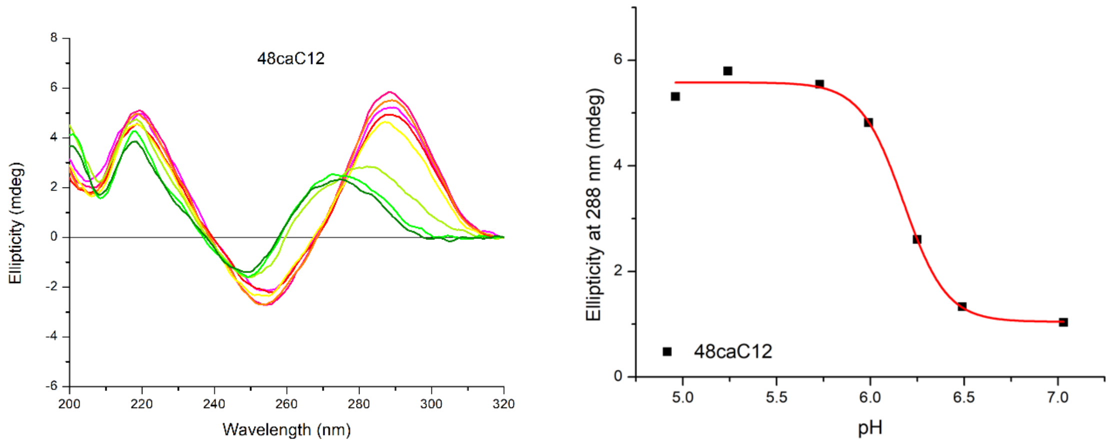

Figure S1Q. CD spectra for epigenetically modified hTeloC. All oligonucleotides were diluted to a final concentration of 10 μM in 10 mM sodium cacodylate with 100 mM sodium chloride at the indicated pH ■pH 5.0; ■pH 5.25; ■pH 5.5; ■pH 5.75; ■pH 6.0; ■pH 6.25; ■pH 6.5; and ■pH 7.0.

Table S3. Transitional pH values for the unmodified control sequence and each of the epigenetically modified hTeloC oligonucleotides.

	pH_T			
Unmodified Control	6.31 ± 0.01			
Cytosine	5mC	5hmC	5fC	5caC
1	6.25 ± 0.03	6.23 ± 0.04	6.20 ± 0.03	6.1 ± 0.02
2	6.48 ± 0.01	6.30 ± 0.05	6.18 ± 0.02	6.31 ± 0.03
3	6.48 ± 0.01	6.31 ± 0.03	6.05 ± 0.03	6.14 ± 0.03
4	6.32 ± 0.01	6.09 ± 0.05	5.97 ± 0.01	5.90 ± 0.01
5	6.37 ± 0.01	6.22 ± 0.04	6.19 ± 0.01	6.16 ± 0.05
6	6.37 ± 0.01	6.21 ± 0.04	6.13 ± 0.07	6.22 ± 0.01
7	6.41 ± 0.01	6.17 ± 0.04	6.24 ± 0.01	6.10 ± 0.02
8	6.46 ± 0.01	6.24 ± 0.27	6.00 ± 0.03	6.34 ± 0.03
9	6.44 ± 0.01	6.15 ± 0.04	6.07 ± 0.04	6.18 ± 0.02
10	6.40 ± 0.02	6.00 ± 0.05	5.96 ± 0.05	5.94 ± 0.02
11	6.38 ± 0.01	6.26 ± 0.12	6.11 ± 0.09	6.16 ± 0.10
12	6.40 ± 0.02	6.19 ± 0.06	6.21 ± 0.02	6.17 ± 0.04

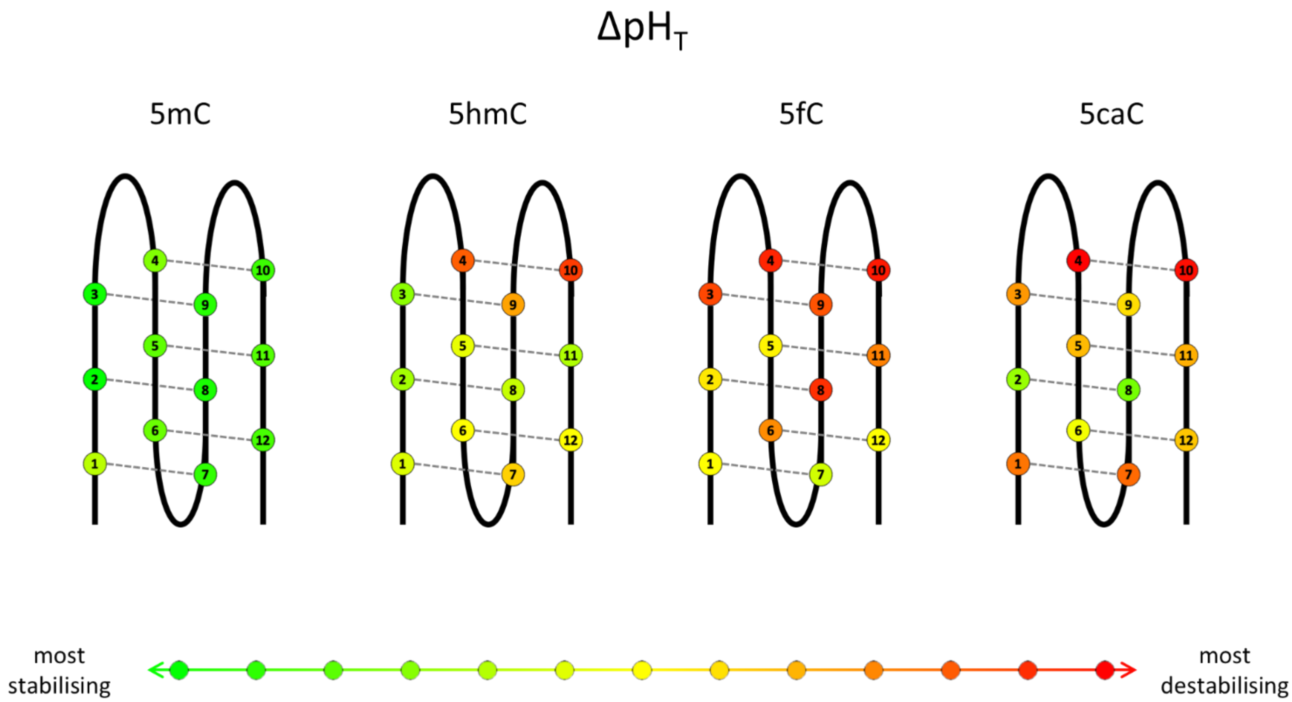

Figure S2. Summary figure showing the effect of epigenetic modification at each position on the transitional pH of the i-motifs formed by the modified hTeloC oligonucleotides.

THERMAL DIFFERENCE SPECTRA OF CYTOSINE MODIFICATIONS

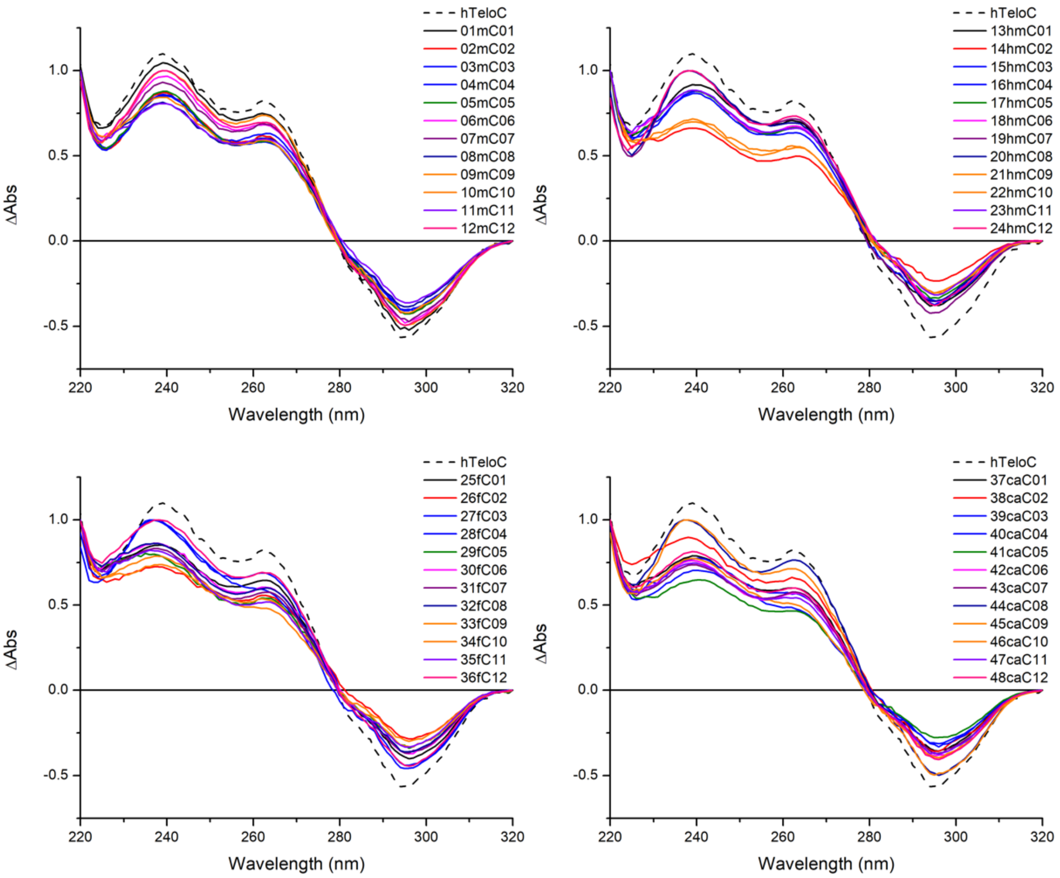

Figure S3. Thermal difference spectra of epigenetically modified oligonucleotides separated by the four different modifications.

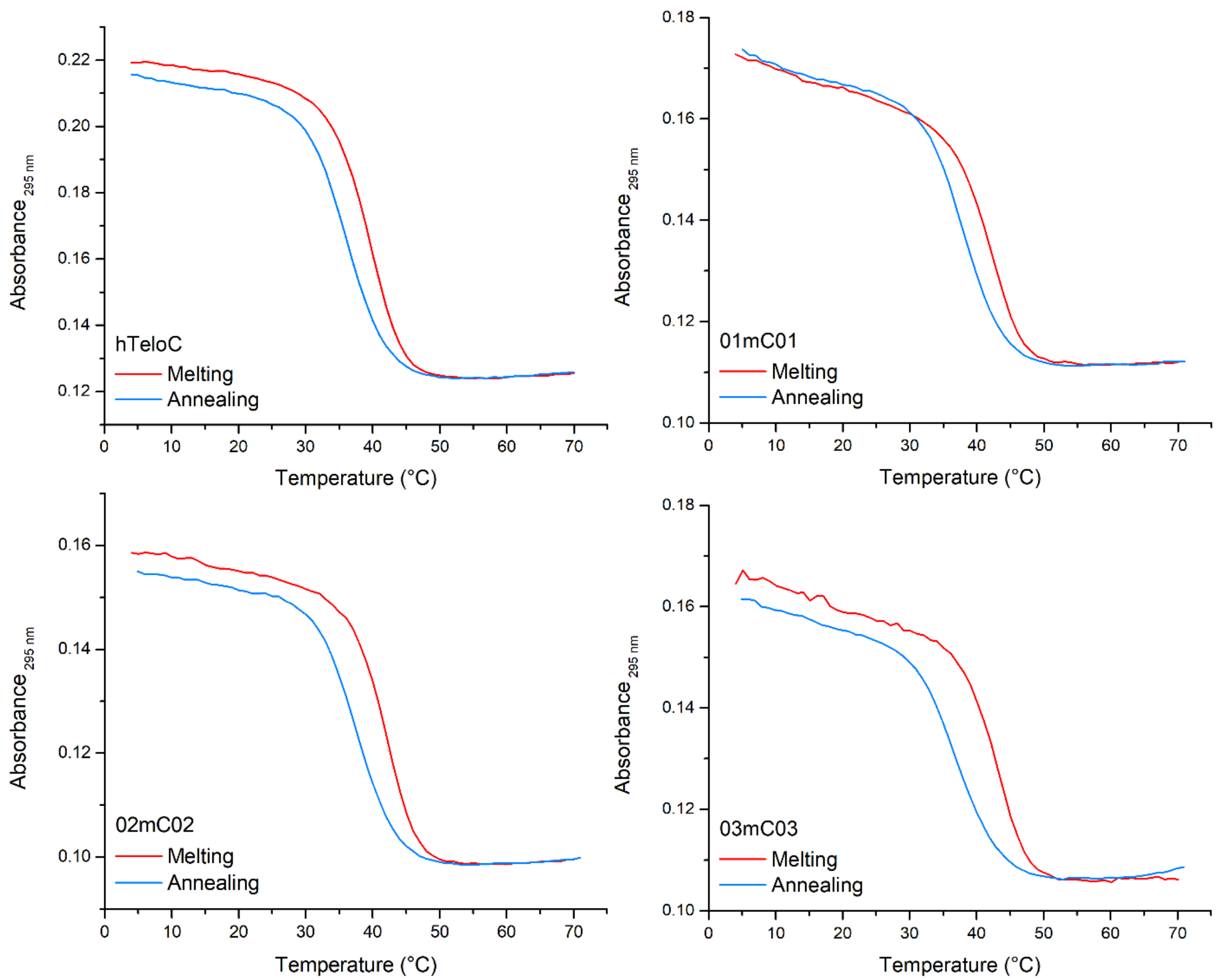

Figure S4A. UV melting and annealing curves for epigenetically modified hTeloC. Each panel is labelled as XX(m, hm, f or ca)CYY, where XX = Oligonucleotide number(01-48); m, hm, f or ca = specific modification, C = cytosine and YY indicates which cytosine is modified (01-12). All oligonucleotides were diluted to a final concentration of 2.5 μM in 10 mM sodium cacodylate with 100 mM sodium chloride at pH 5.5.

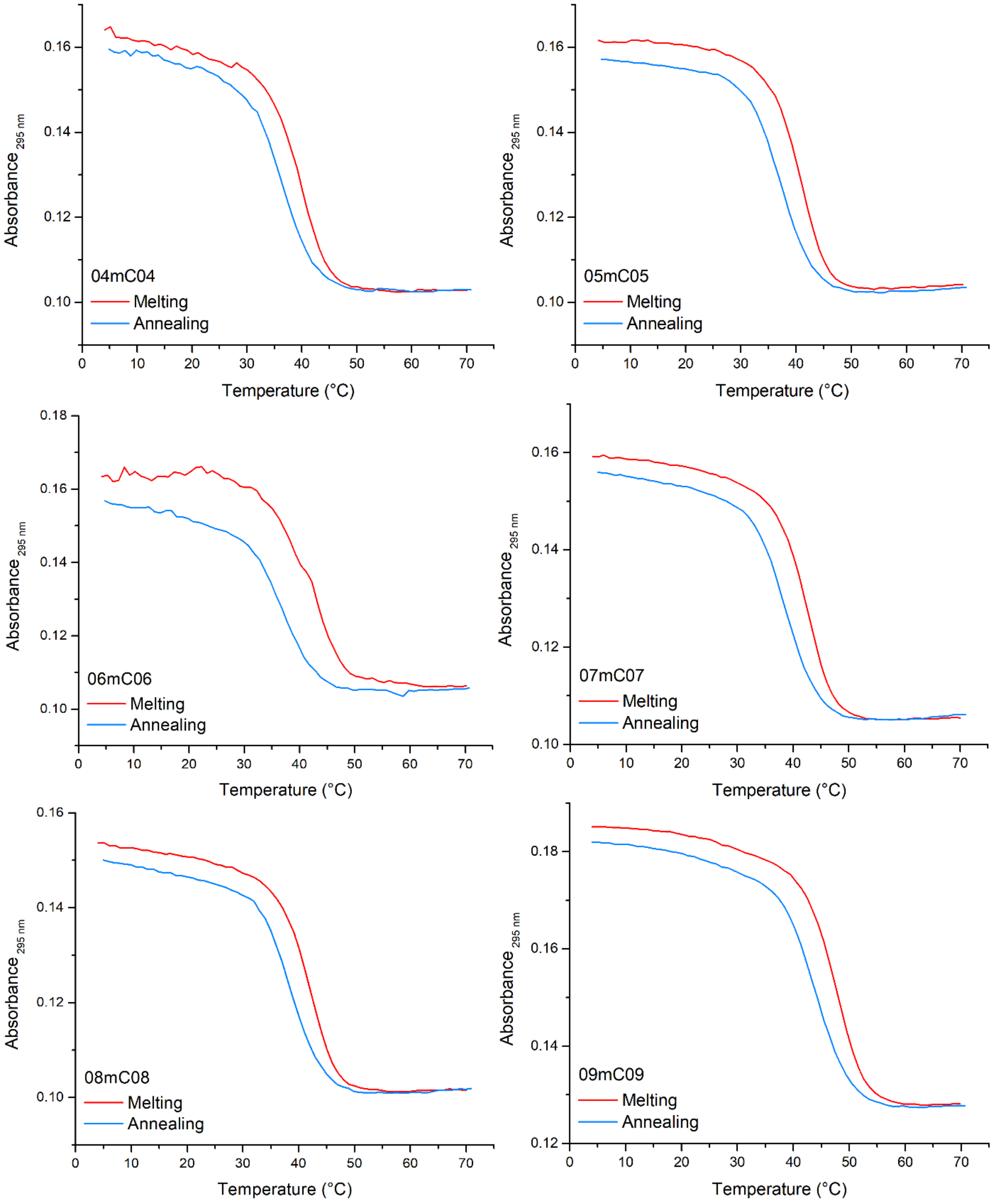

Figure S4B. UV melting and annealing curves for epigenetically modified hTeloC. Each panel is labelled as XX(m, hm, f or ca)CYY, where XX = Oligonucleotide number(01-48); m, hm, f or ca = specific modification, C = cytosine and YY indicates which cytosine is modified (01-12). All oligonucleotides were diluted to a final concentration of 2.5 µM in 10 mM sodium cacodylate with 100 mM sodium chloride at pH 5.5.

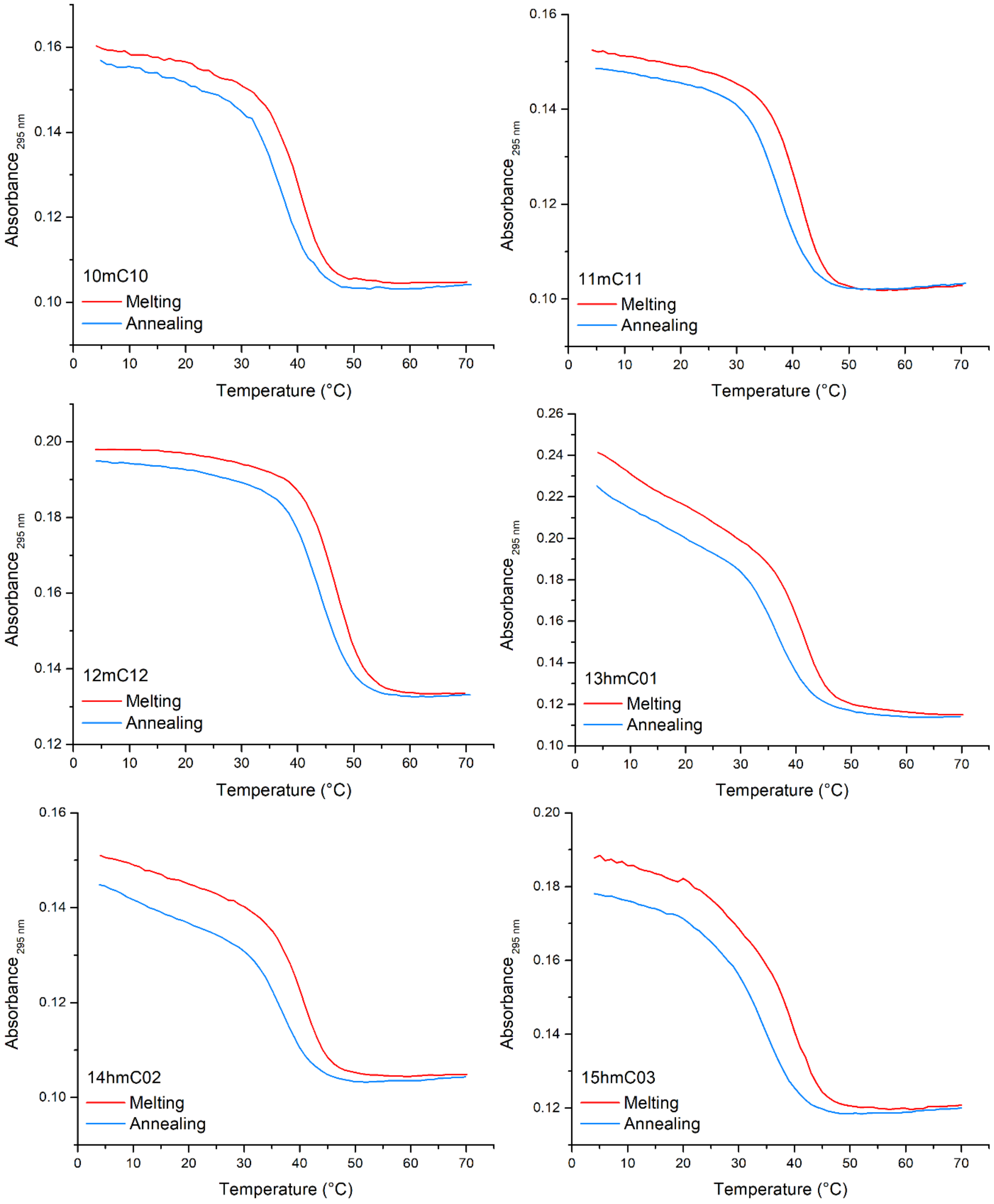

Figure S4C. UV melting and annealing curves for epigenetically modified hTeloC. Each panel is labelled as XX(m, hm, f or ca)CYY, where XX = Oligonucleotide number(01-48); m, hm, f or ca = specific modification, C = cytosine and YY indicates which cytosine is modified (01-12). All oligonucleotides were diluted to a final concentration of 2.5 µM in 10 mM sodium cacodylate with 100 mM sodium chloride at pH 5.5.

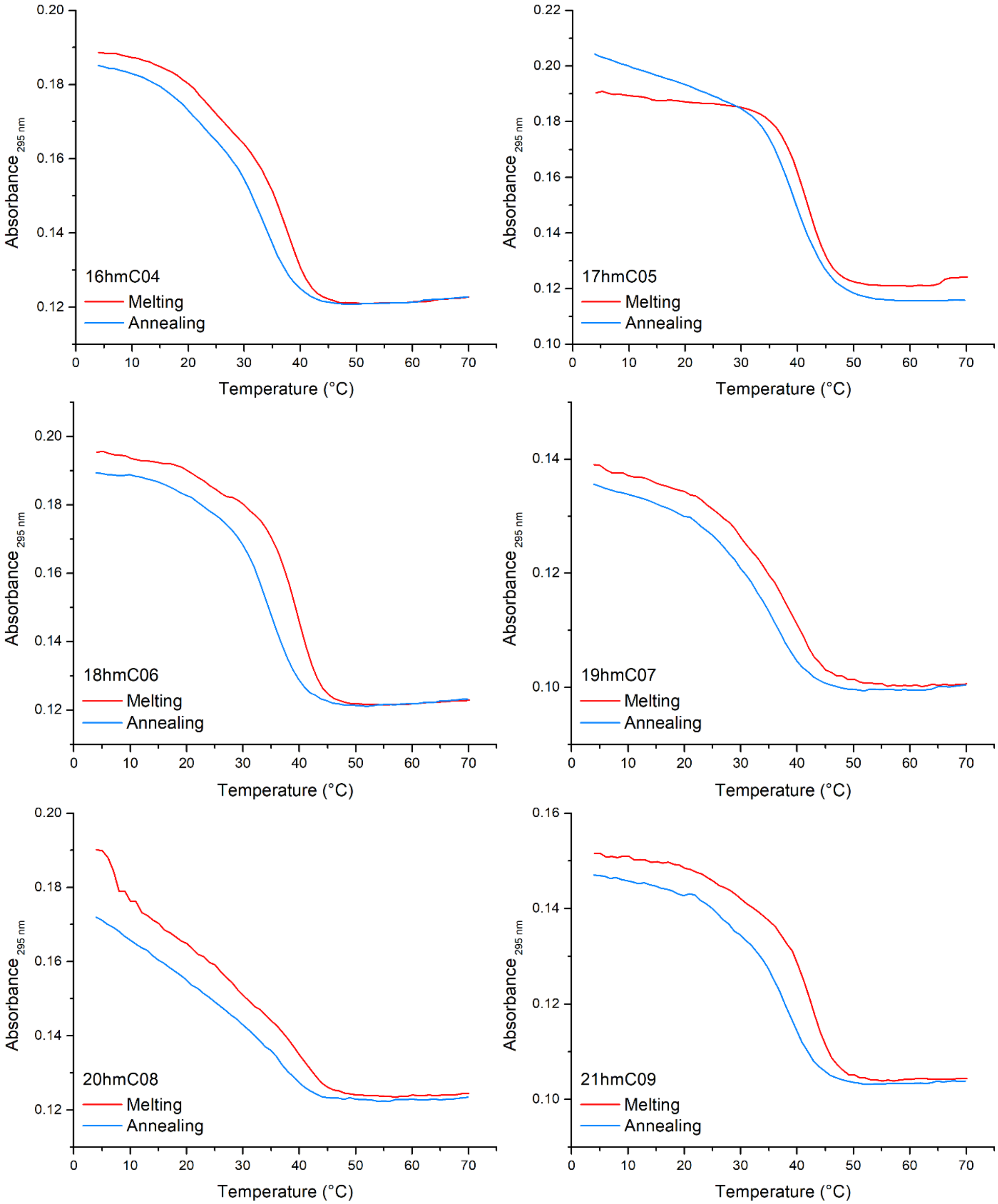

Figure S4D. UV melting and annealing curves for epigenetically modified hTeloC. Each panel is labelled as XX(m, hm, f or ca)CYY, where XX = Oligonucleotide number(01-48); m, hm, f or ca = specific modification, C = cytosine and YY indicates which cytosine is modified (01-12). All oligonucleotides were diluted to a final concentration of 2.5 µM in 10 mM sodium cacodylate with 100 mM sodium chloride at pH 5.5.

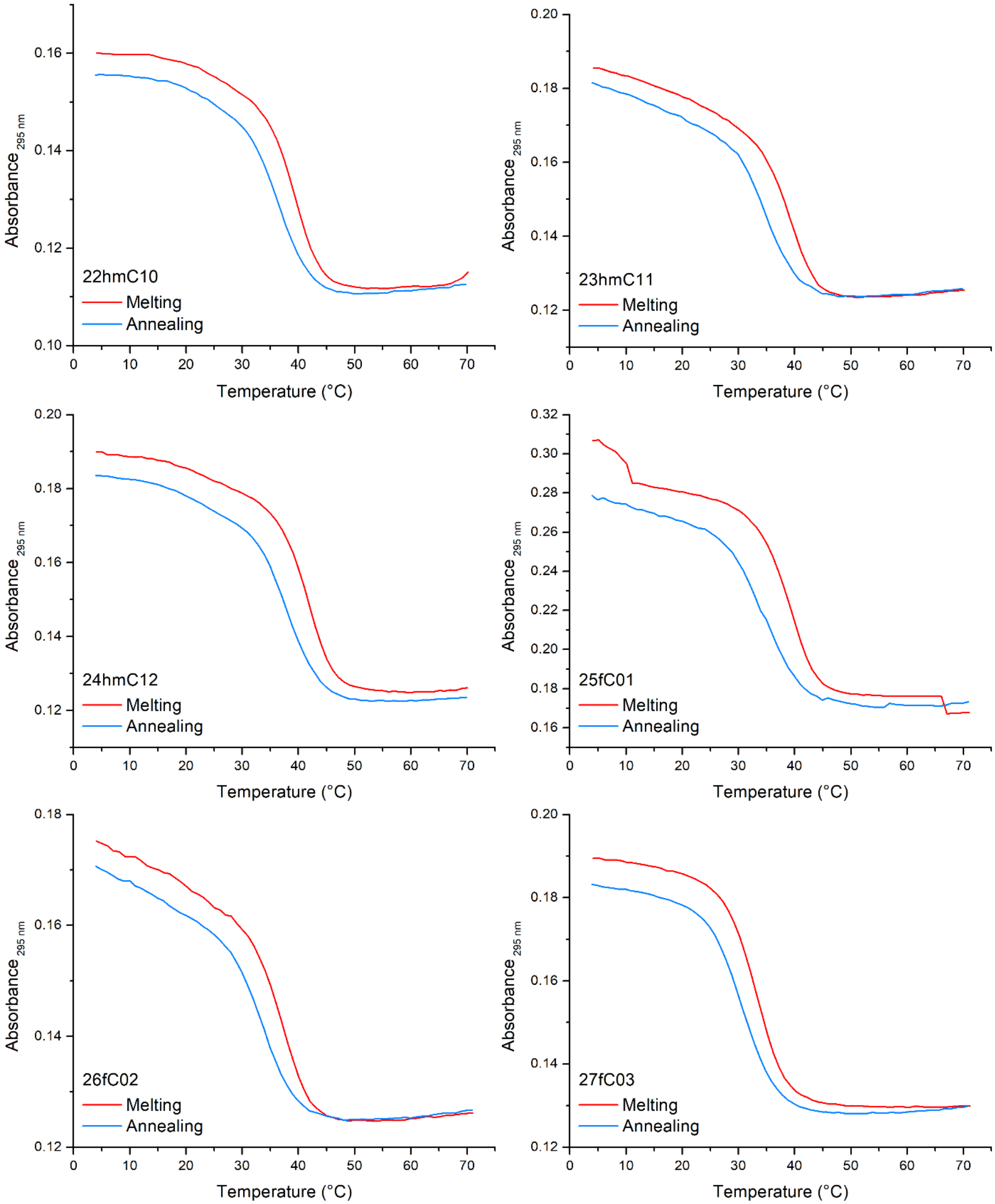

Figure S4E. UV melting and annealing curves for epigenetically modified hTeloC. Each panel is labelled as XX(m, hm, f or ca)CYY, where XX = Oligonucleotide number(01-48); m, hm, f or ca = specific modification, C = cytosine and YY indicates which cytosine is modified (01-12). All oligonucleotides were diluted to a final concentration of 2.5 μM in 10 mM sodium cacodylate with 100 mM sodium chloride at pH 5.5.

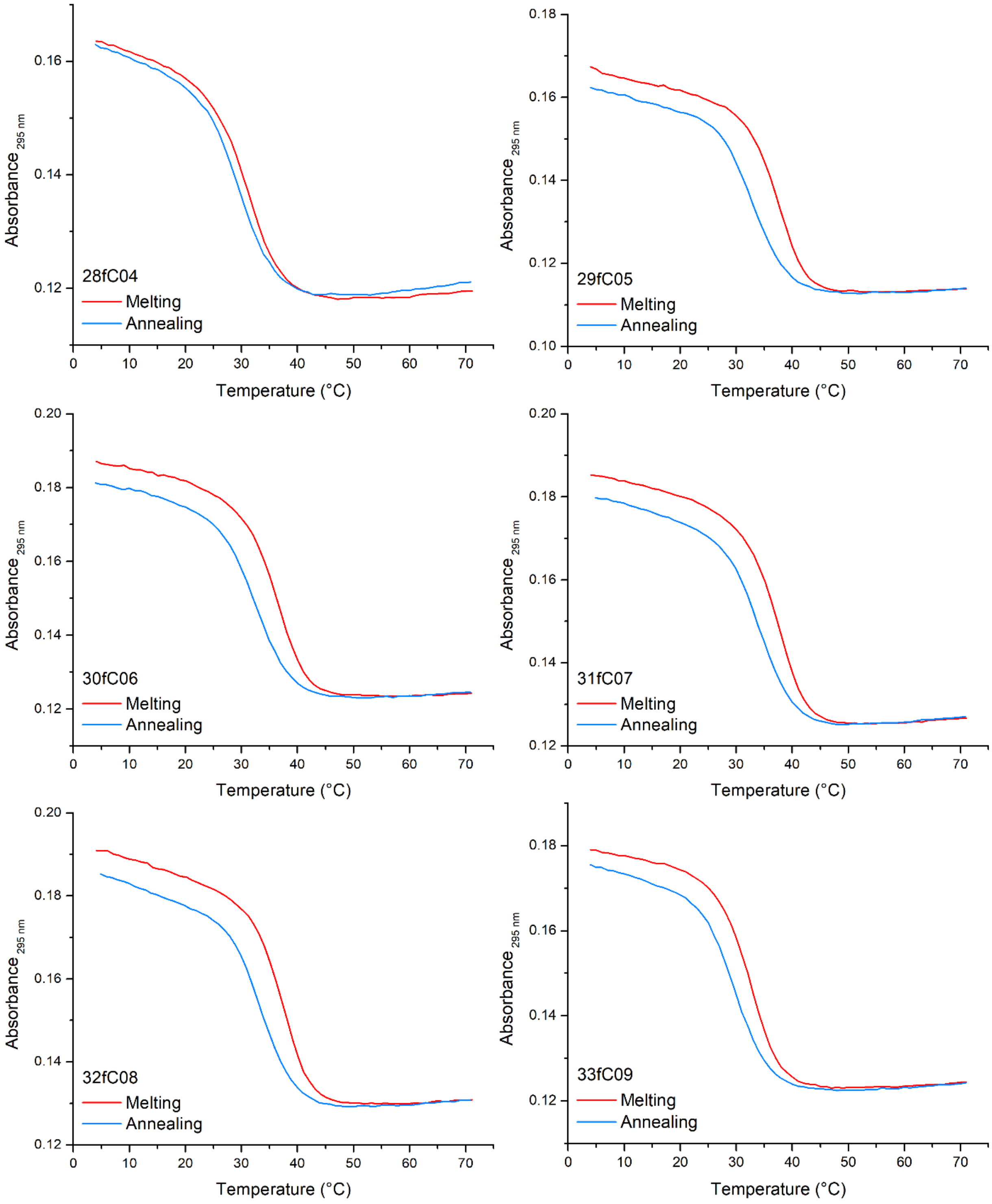

Figure S4F. UV melting and annealing curves for epigenetically modified hTeloC. Each panel is labelled as XX(m, hm, f or ca)CYY, where XX = Oligonucleotide number(01-48); m, hm, f or ca = specific modification, C = cytosine and YY indicates which cytosine is modified (01-12). All oligonucleotides were diluted to a final concentration of 2.5 µM in 10 mM sodium cacodylate with 100 mM sodium chloride at pH 5.5.

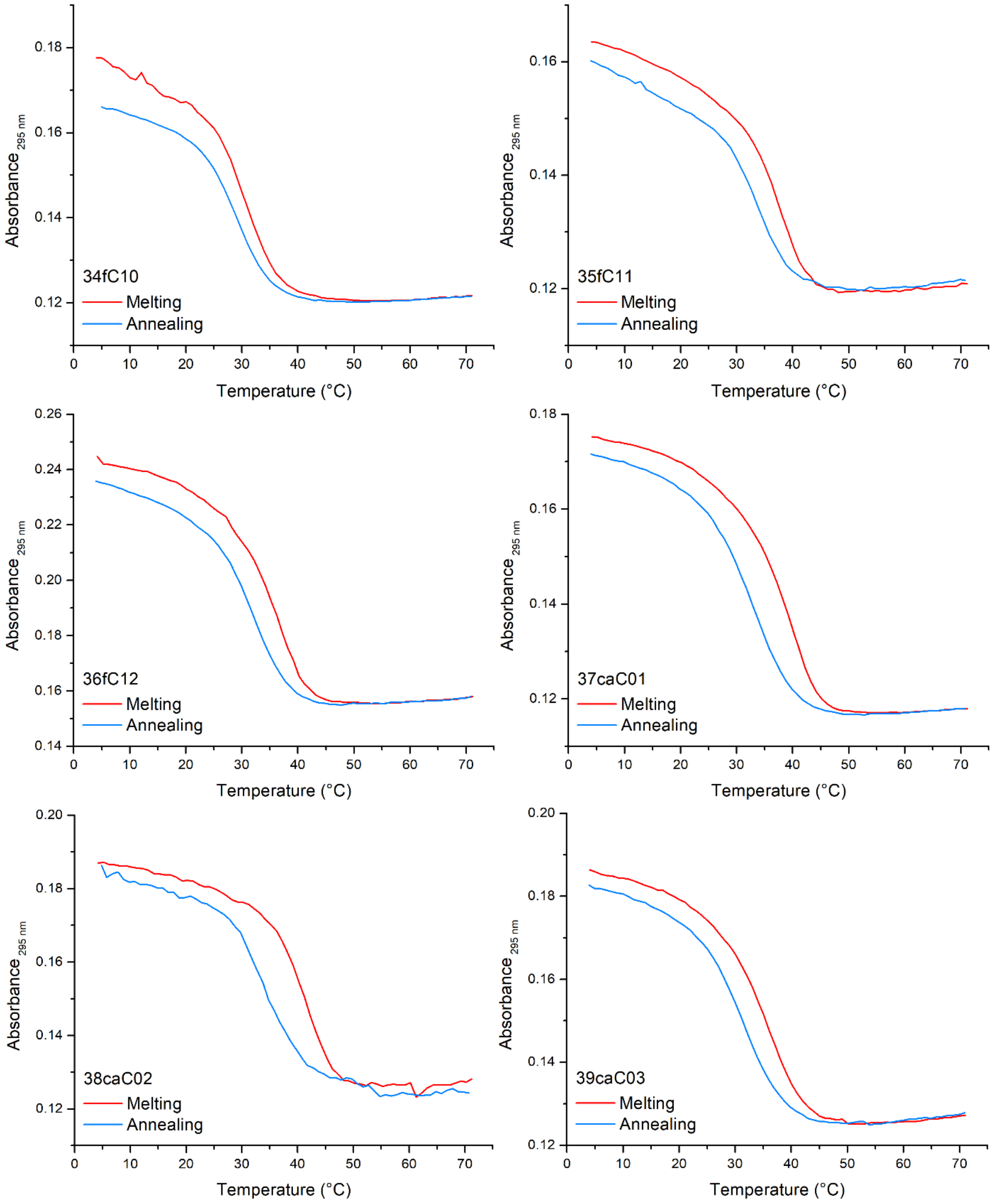

Figure S4G. UV melting and annealing curves for epigenetically modified hTeloC. Each panel is labelled as XX(m, hm, f or ca)CYY, where XX = Oligonucleotide number(01-48); m, hm, f or ca = specific modification, C = cytosine and YY indicates which cytosine is modified (01-12). All oligonucleotides were diluted to a final concentration of 2.5 μM in 10 mM sodium cacodylate with 100 mM sodium chloride at pH 5.5.

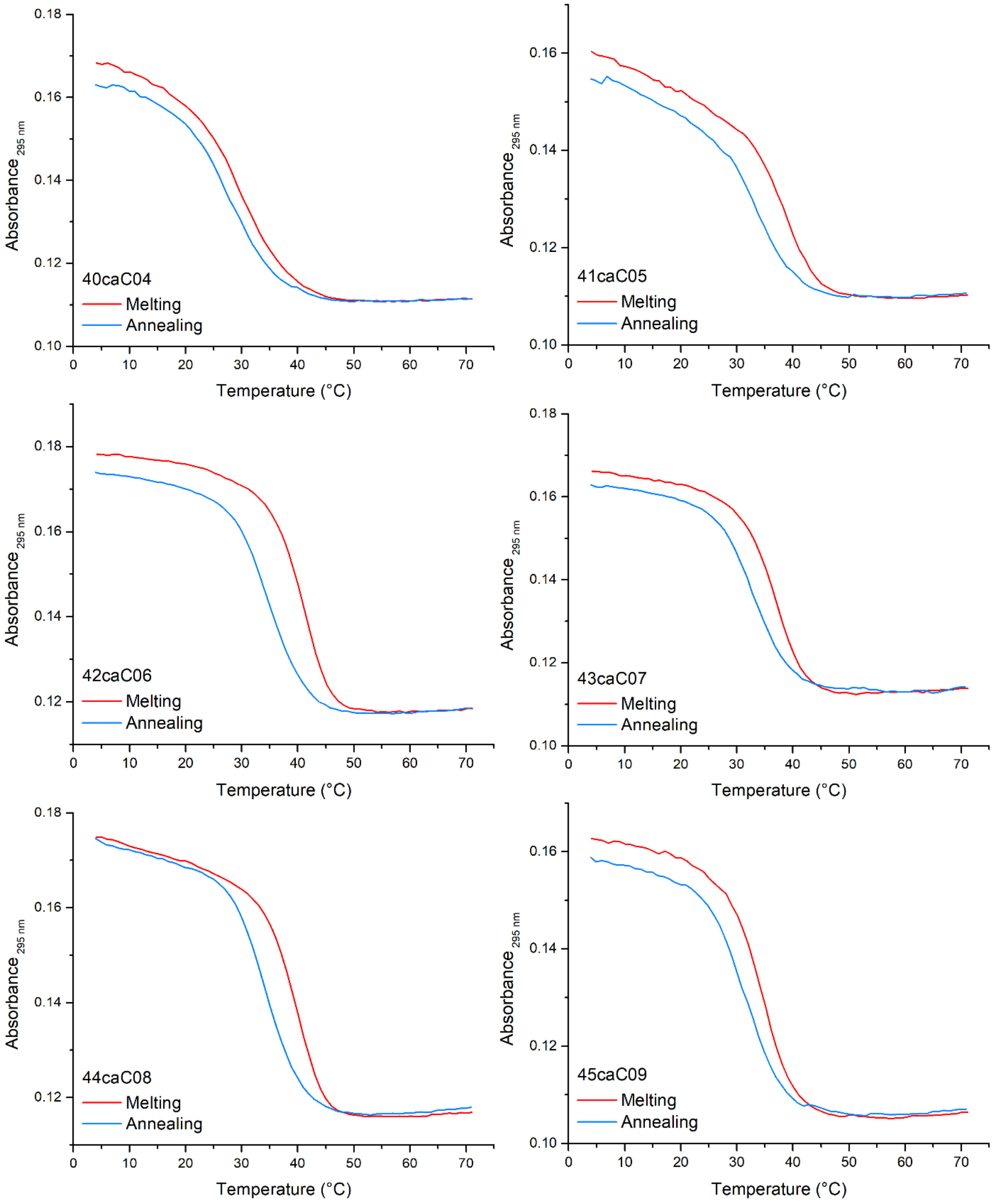

Figure S4H. UV melting and annealing curves for epigenetically modified hTeloC. Each panel is labelled as XX(m, hm, f or ca)CYY, where XX = Oligonucleotide number(01-48); m, hm, f or ca = specific modification, C = cytosine and YY indicates which cytosine is modified (01-12). All oligonucleotides were diluted to a final concentration of 2.5 µM in 10 mM sodium cacodylate with 100 mM sodium chloride at pH 5.5.

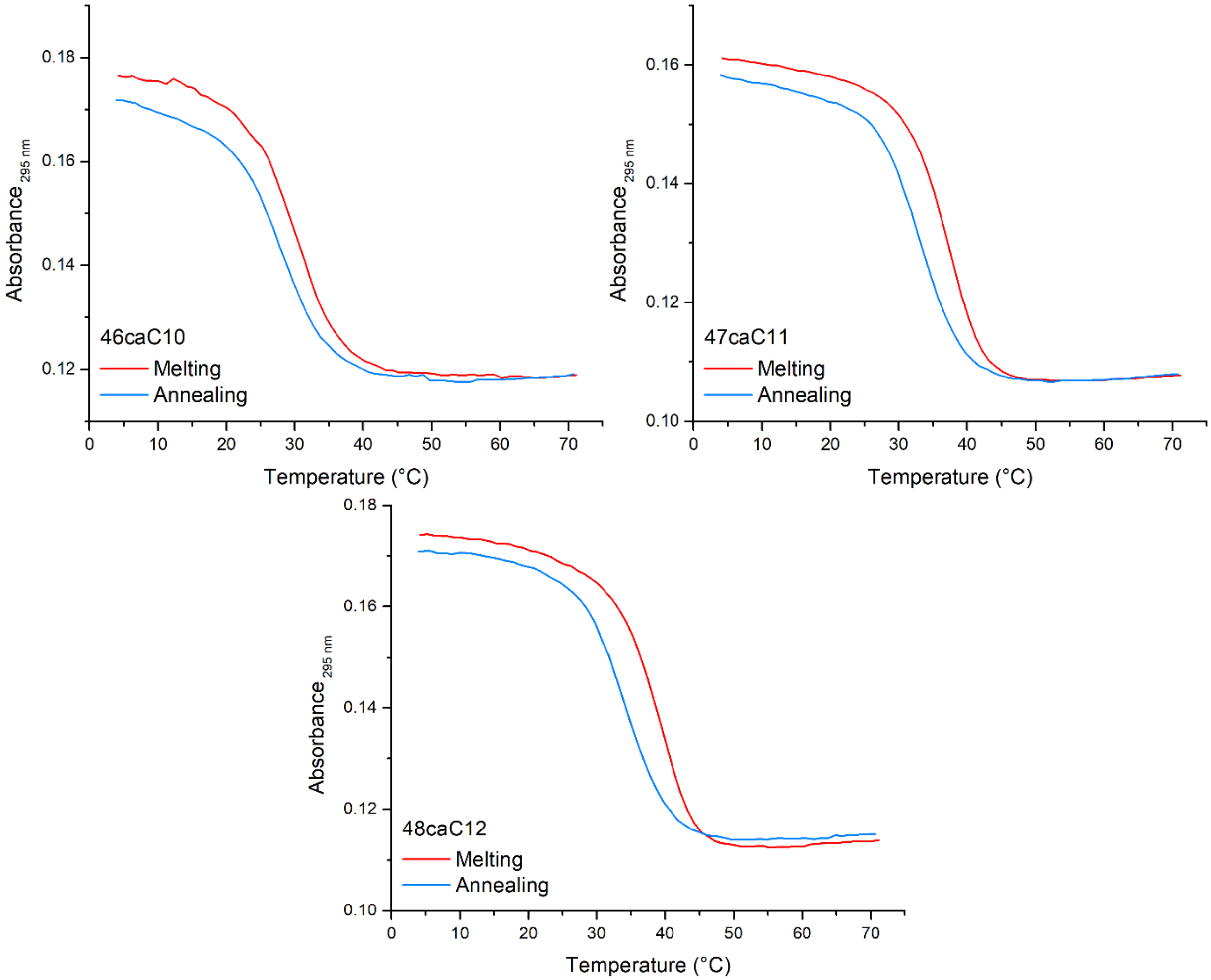

Figure S4I. UV melting and annealing curves for epigenetically modified hTeloC. Each panel is labelled as XX(m, hm, f or ca)CYY, where XX = Oligonucleotide number(01-48); m, hm, f or ca = specific modification, C = cytosine and YY indicates which cytosine is modified (01-12). All oligonucleotides were diluted to a final concentration of 2.5 μM in 10 mM sodium cacodylate with 100 mM sodium chloride at pH 5.5.

Table S4. Melting temperatures of the unmodified control sequence and each of the epigenetically modified hTeloC oligonucleotides. * = p-value ≤ 0.05

	T_m (°C)			
Unmodified Control	39.7 ± 0.52			
Cytosine	5mC	5hmC	5fC	5caC
1	42.0 ± 0.00*	40.9 ± 0.60	39.4 ± 0.57	39.9 ± 0.58
2	41.7 ± 0.58*	40.1 ± 0.00	36.7 ± 0.58*	41.2 ± 0.00*
3	42.8 ± 0.57*	39.7 ± 0.58	33.9 ± 0.57*	35.4 ± 0.58*
4	39.8 ± 0.58	37.4 ± 0.60*	31.4 ± 0.58*	30.1 ± 2.00*
5	40.5 ± 0.58	41.6 ± 0.59*	37.3 ± 0.58*	38.1 ± 0.01*
6	40.6 ± 1.53	39.2 ± 0.01	36.1 ± 0.01*	40.9 ± 0.57
7	42.7 ± 0.58*	40.1 ± 1.42	37.1 ± 0.01*	36.6 ± 0.58*
8	41.7 ± 0.57*	39.4 ± 0.57	37.5 ± 0.57*	40.1 ± 0.01
9	44.3 ± 2.56*	42.5 ± 0.58*	32 ± 0.01*	34.4 ± 1.15*
10	39.5 ± 0.57	38.9 ± 0.58	29.4 ± 1.15*	29.9 ± 1.15*
11	40.9 ± 0.58	38.9 ± 0.59	37.2 ± 0.01*	36.9 ± 0.58*
12	43.3 ± 2.13*	41.4 ± 0.58*	36.2 ± 0.01*	39.2 ± 0.01

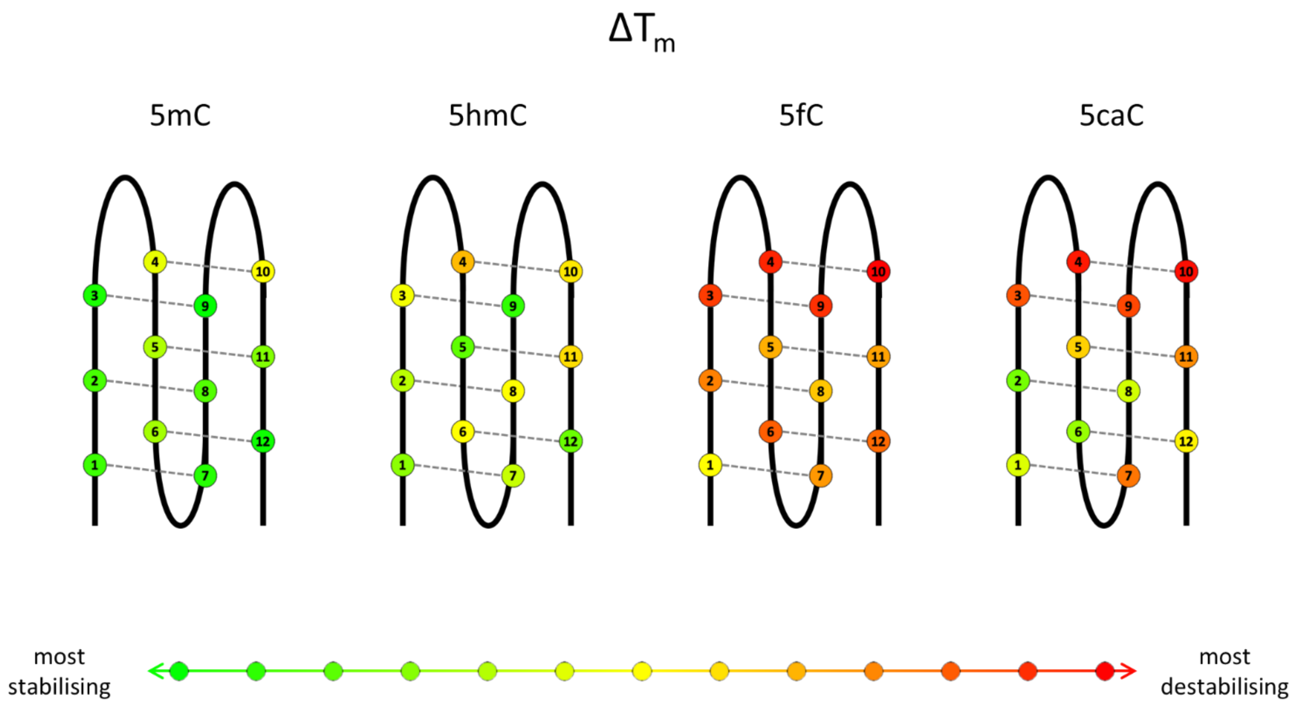

Figure S5. Summary figure showing the effect of epigenetic modification at each position on the melting temperature of the i-motifs formed by the modified hTeloC oligonucleotides.

Table S5. Difference in enthalpy and entropy change calculated for each epigenetically modified oligonucleotide using the UV-melt curves. * = p-value ≤ 0.05

	ΔΔH				ΔΔS			
Cytosine	5mC	5hC	5fC	5caC	5mC	5hC	5fC	5caC
1	-7.4 ± 3.8*	-1.1 ± 9.9	-4.8 ± 8.1	7.5 ± 4.4*	-21.8 ± 12.1*	-2.6 ± 31.2	-16.1 ± 26.5	23.1 ± 14.3*
2	-10.2 ± 2.3*	-5.4 ± 1.2*	-3.3 ± 3.2	-3.0 ± 5.2	-30.5 ± 7.4*	-16.9 ± 4.4*	-12.9 ± 10.7	-8.8 ± 16.8
3	-14.7 ± 5.9*	4.3 ± 8.3	7.0 ± 3.1*	23.1 ± 4.5*	-43.5 ± 18.9*	13.2 ± 26.6	16.9 ± 10.3*	71.1 ± 14.9*
4	-0.7 ± 3.4	11.9 ± 2.9*	26.0 ± 5.2*	43.5 ± 3.3*	-2.2 ± 11.2	34.8 ± 9.6*	78.0 ± 17.1*	135.2 ± 88.9*
5	-2.8 ± 3.8	-5.3 ± 6.0	-6.5 ± 6.4	11.6 ± 2.4*	-8.2 ± 12.2	-15.9 ± 18.9	-23.0 ± 20.6	36.6 ± 7.6*
6	-1.5 ± 4.5	-14.3 ± 5.6*	2.6 ± 6.9	-5.2 ± 4.0	-3.0 ± 14.3	-45.8 ± 18.4*	5.1 ± 22.7	-15.9 ± 13.1
7	-12.7 ± 1.9*	20.3 ± 3.5*	4.7 ± 3.2*	8.2 ± 3.5*	-37.9 ± 6.3*	63.5 ± 11.4*	13.0 ± 10.9	23.9 ± 11.8*
8	-8.9 ± 1.9*	-2.3 ± 1.9	-1.3 ± 6.5	-3.7 ± 2.5*	-26.4 ± 6.5*	-7.5 ± 6.4	-6.5 ± 21.0	-11.9 ± 8.3*
9	0.7 ± 4.2	-1.9 ± 3.8	3.0 ± 5.9	13.3 ± 2.4*	8.7 ± 13.6	-4.8 ± 12.2	3.2 ± 19.5	38.6 ± 7.9*
10	-1.6 ± 3.3	-2.2 ± 3.8	12.9 ± 5.4*	28.4 ± 2.6*	-4.8 ± 11.0	-8.1 ± 12.2	34.4 ± 18.3*	84.0 ± 8.5*
11	-3.8 ± 2.8*	-11.6 ± 6.4*	11.9 ± 7.1*	3.5 ± 2.0*	-11.3 ± 9.1*	-37.5 ± 20.9*	36.4 ± 23.4*	8.8 ± 7.0*
12	-2.1 ± 8.4	-12.7 ± 3.4*	-1.1 ± 4.5*	5.5 ± 1.5*	-0.2 ± 25.7	-38.6 ± 10.8*	-7.1 ± 14.7*	16.7 ± 5.4*

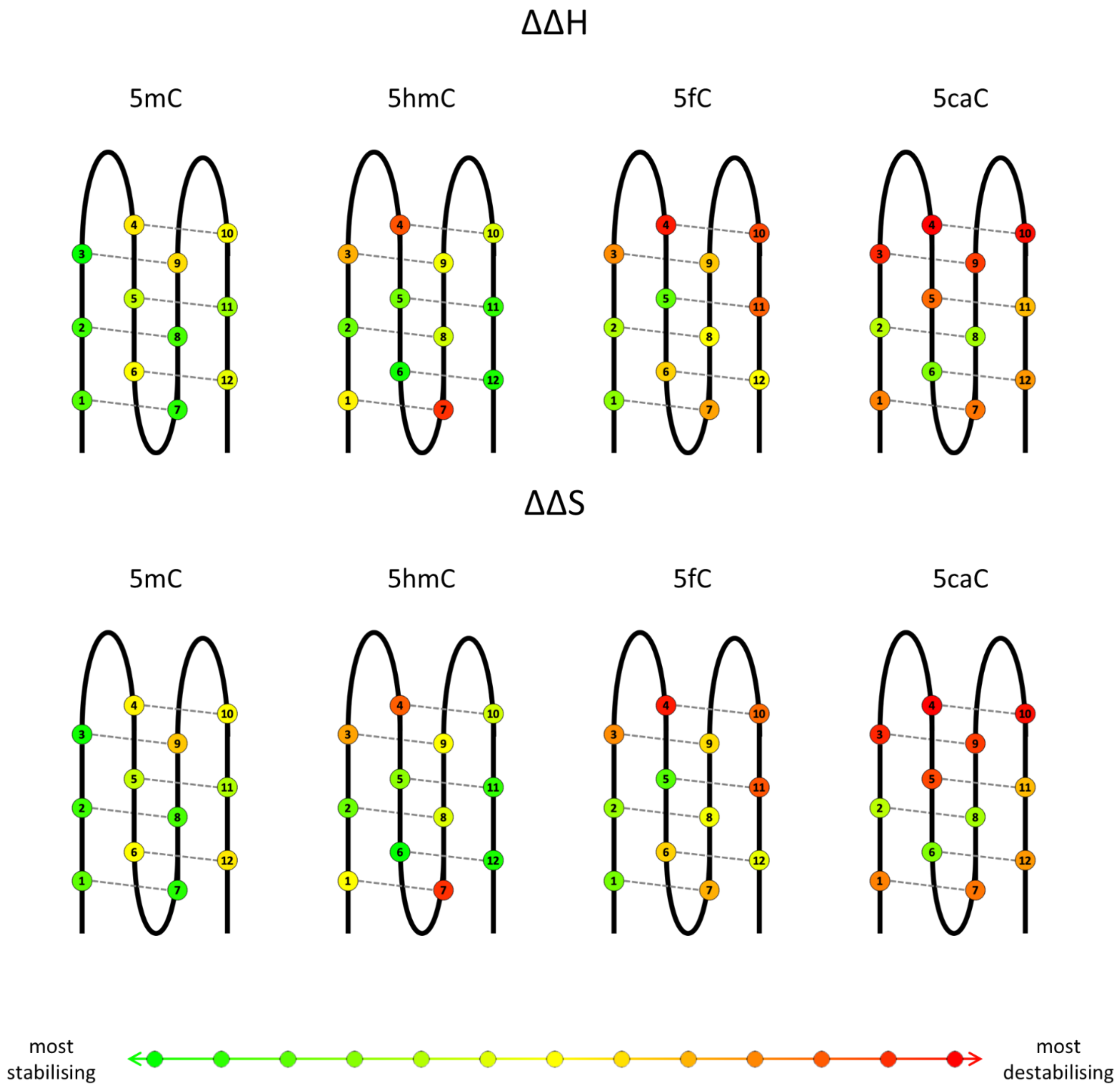

Figure S6. Summary figure showing the effect of epigenetic modification at each position on the thermodynamic characteristics of the i-motifs formed by the modified hTeloC oligonucleotides.

CIRCULAR DICHROISM FOR THE THYMINE SCREEN

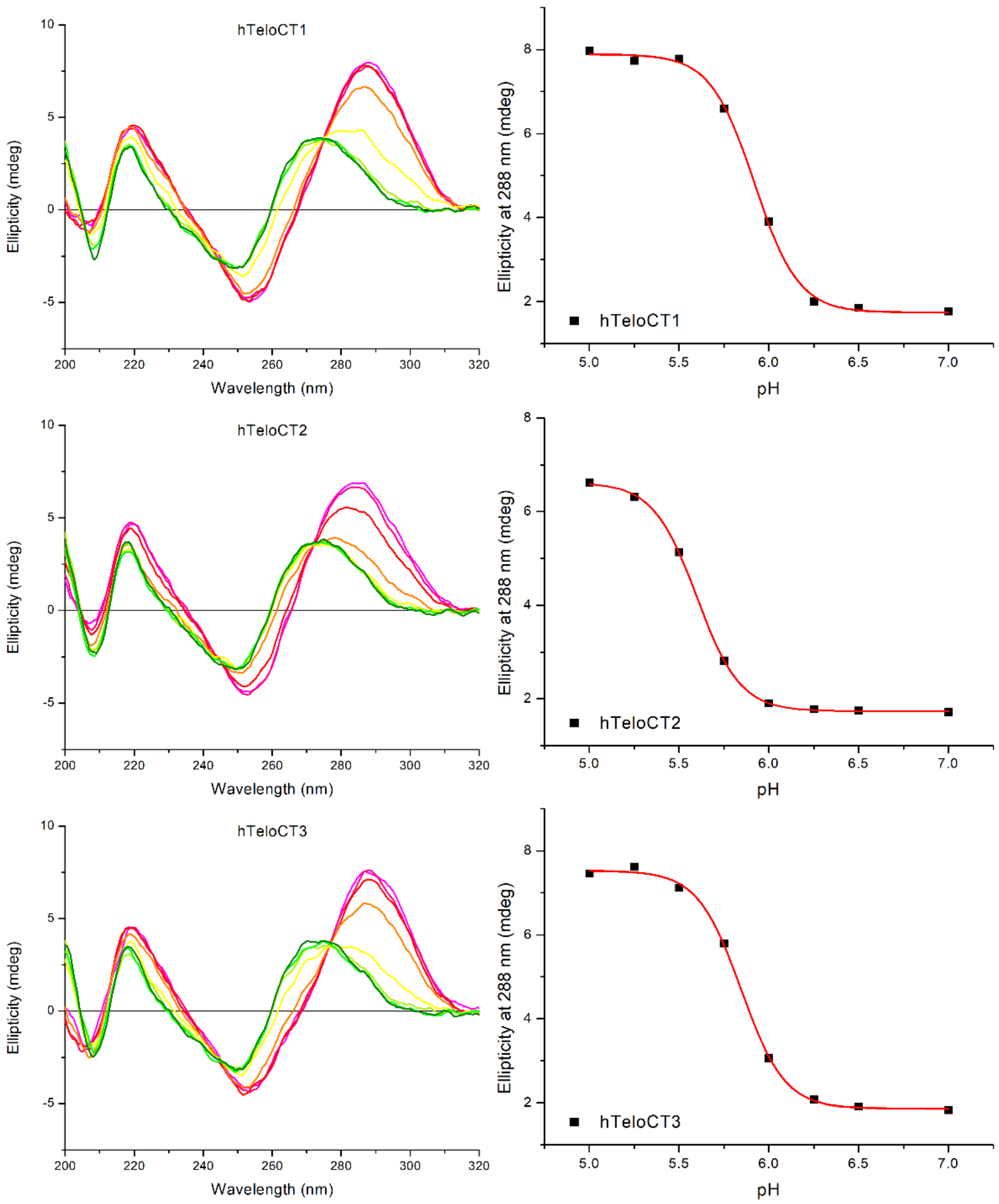

Figure S7A. CD spectra for each of the mutated hTeloCTx oligonucleotides. All oligonucleotides were diluted to a final concentration of 10 μM in 10 mM sodium cacodylate with 100 mM sodium chloride at the indicated pH ■pH 5.0; ■pH 5.25; ■pH 5.5; ■pH 5.75; ■pH 6.0; ■pH 6.25; ■pH 6.5; and ■pH 7.0.

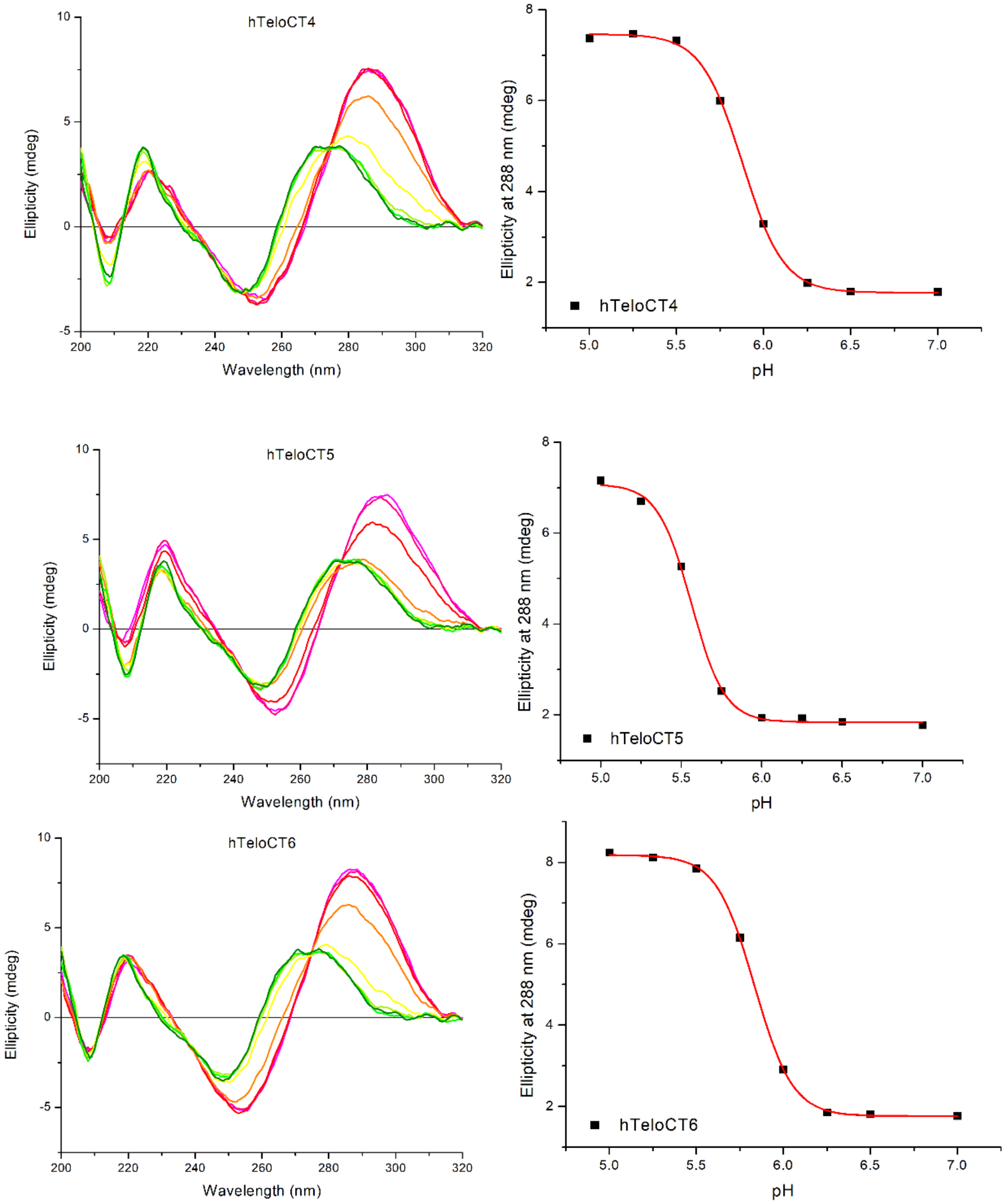

Figure S7B. CD spectra for each of the mutated hTeloCTx oligonucleotides. All oligonucleotides were diluted to a final concentration of 10 μM in 10 mM sodium cacodylate with 100 mM sodium chloride at the indicated pH ■pH 5.0; ■pH 5.25; ■pH 5.5; ■pH 5.75; ■pH 6.0; ■pH 6.25; ■pH 6.5; and ■pH 7.0.

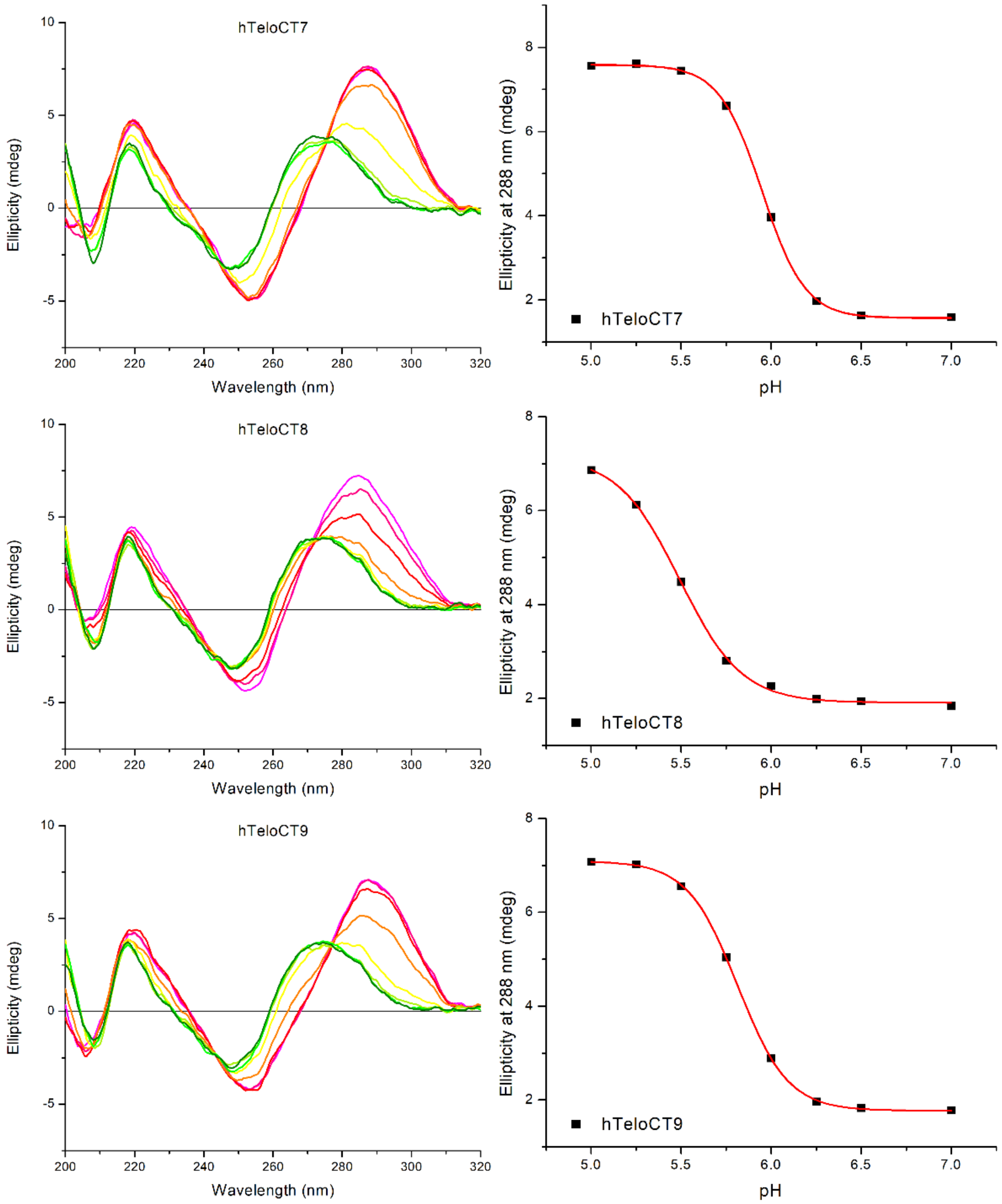

Figure S7C. CD spectra for each of the mutated hTeloCTx oligonucleotides. All oligonucleotides were diluted to a final concentration of 10 μM in 10 mM sodium cacodylate with 100 mM sodium chloride at the indicated pH ■pH 5.0; ■pH 5.25; ■pH 5.5; ■pH 5.75; ■pH 6.0; ■pH 6.25; ■pH 6.5; and ■pH 7.0.

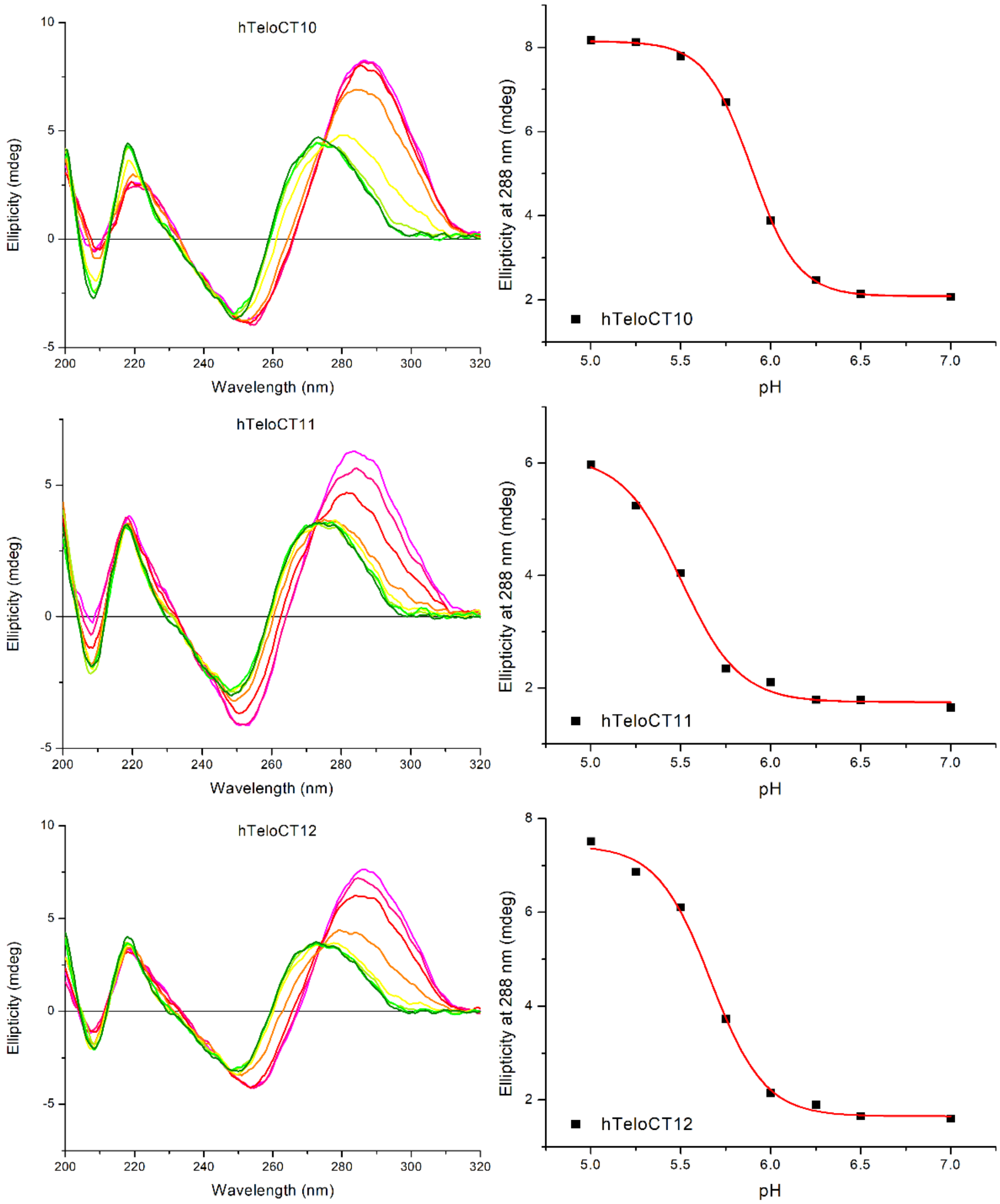

Figure S7D. CD spectra for each of the mutated hTeloCTx oligonucleotides. All oligonucleotides were diluted to a final concentration of 10 µM in 10 mM sodium cacodylate with 100 mM sodium chloride at the indicated pH ■pH 5.0; ■pH 5.25; ■pH 5.5; ■pH 5.75; ■pH 6.0; ■pH 6.25; ■pH 6.5; and ■pH 7.0.

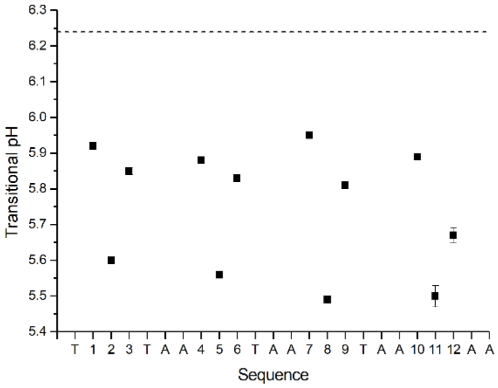

Figure S8. Transitional pH of each of the mutated hTeloCTx oligonucleotides compared to the unmodified control sequence (line).

Table S6. Transitional pH and melting temperature values for the unmodified control sequence and each of the mutated hTeloCTx oligonucleotides. * = p-value ≤ 0.05

	pH_T	T_m (°C)
Unmodified Control	6.31 ± 0.00	39.7 ± 0.52
Mutated Position		
1	5.92 ± 0.01	33.9 ± 0.04*
2	5.60 ± 0.00	22.0 ± 0.03*
3	5.85 ± 0.01	32.4 ± 0.01*
4	5.88 ± 0.01	32.6 ± 0.08*
5	5.56 ± 0.01	24.9 ± 0.45*
6	5.83 ± 0.00	31.6 ± 0.04*
7	5.95 ± 0.00	34.7 ± 0.08*
8	5.49 ± 0.01	23.6 ± 0.17*
9	5.81 ± 0.00	31.7 ± 0.01*
10	5.89 ± 0.01	34.0 ± 0.07*
11	5.50 ± 0.03	27.5 ± 0.18*
12	5.67 ± 0.02	27.6 ± 0.07*

UV SPECTROSCOPY FOR THE THYMINE SCREEN

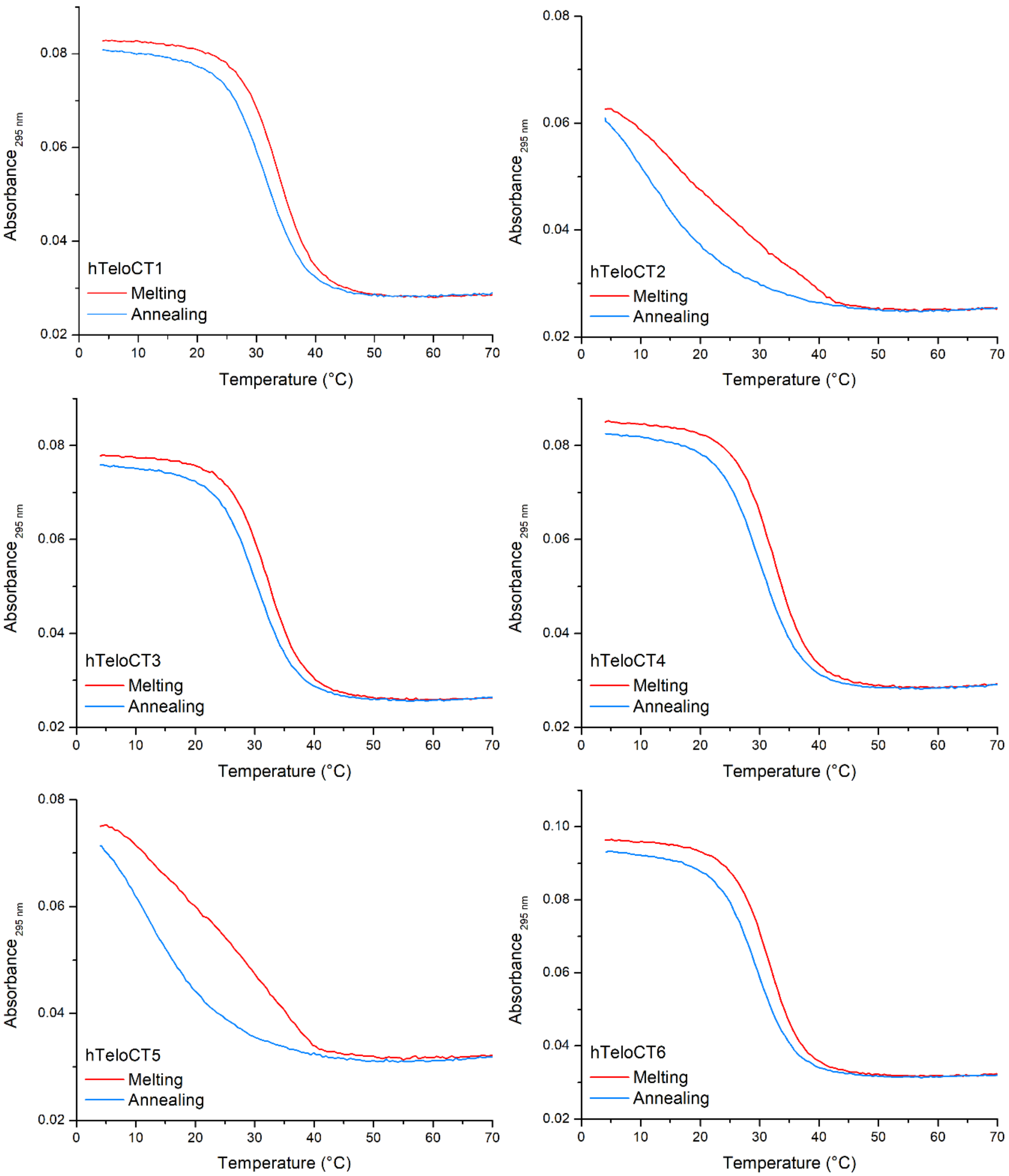

Figure S9A. UV melting and annealing curves for mutated hTeloCTx oligonucleotides. All oligonucleotides were diluted to a final concentration of 2.5 μM in 10 mM sodium cacodylate with 100 mM sodium chloride at pH 5.5.

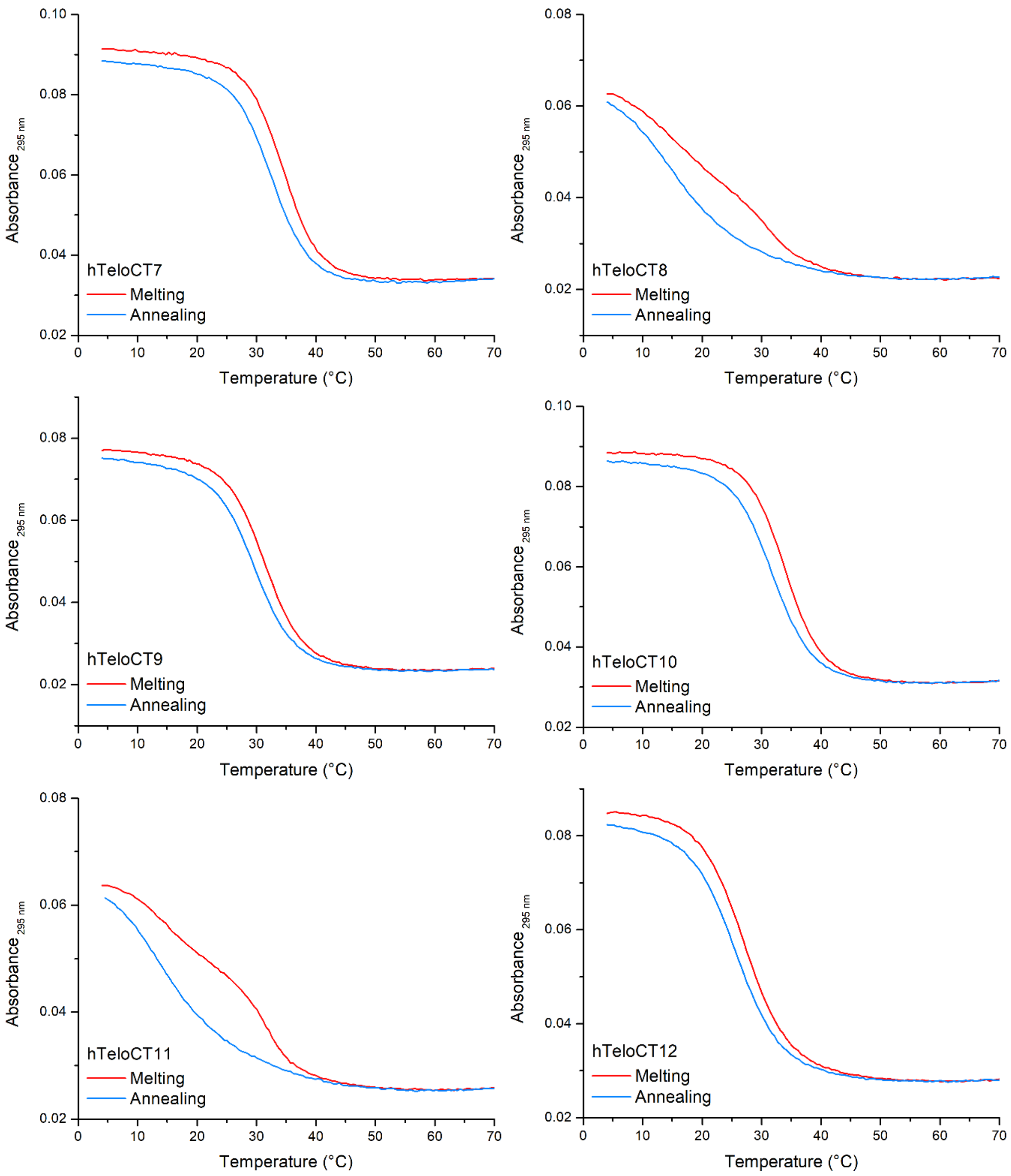

Figure S9B. UV melting and annealing curves for mutated hTeloCTx oligonucleotides. All oligonucleotides were diluted to a final concentration of 2.5 μM in 10 mM sodium cacodylate with 100 mM sodium chloride at pH 5.5.

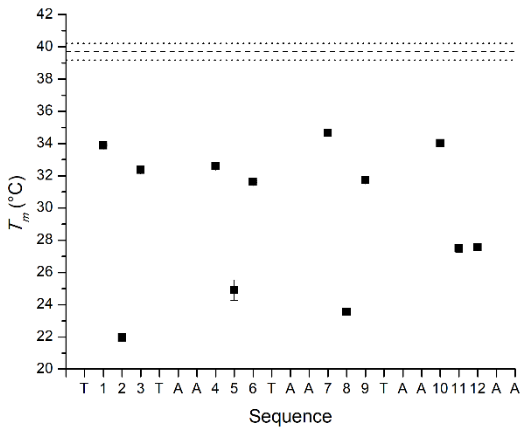

Figure S10. Melting temperature of each of the mutated hTeloCTx oligonucleotides compared to the unmodified control sequence (line).

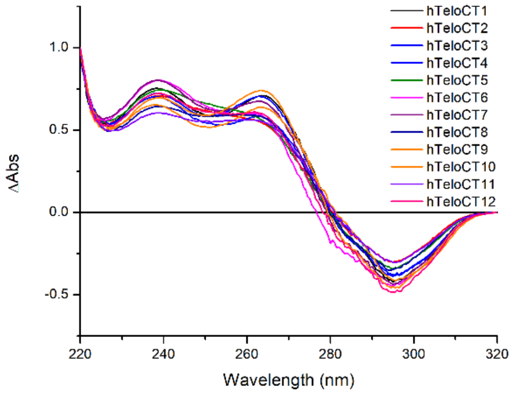

Figure S11. Thermal difference spectra of mutated hTeloCTx oligonucleotides.

Table S7. Difference in enthalpy and entropy change calculated for each hTeloCTx oligonucleotide using the UV-melt curves * = p-value ≤ 0.05

Mutated Position	ΔΔH	ΔΔS
1	22.5 ± 1.4*	67.6 ± 5.0*
2	63.3 ± 1.0*	197.8 ± 3.7*
3	21.4 ± 1.8*	63.1 ± 6.1*
4	21.9 ± 1.4*	64.7 ± 4.7*
5	56.8 ± 1.8*	176.6 ± 6.2*
6	16.8 ± 2.8*	47.6 ± 9.7*
7	18 ± 1.0*	53.4 ± 3.6*
8	55 ± 1.2*	170.1 ± 4.2*
9	24 ± 1.5*	71.2 ± 5.2*
10	21.3 ± 0.9*	63.8 ± 3.3*
11	41.5 ± 1.8*	126.6 ± 6.3*
12	33.9 ± 2.4*	101.4 ± 8.2*

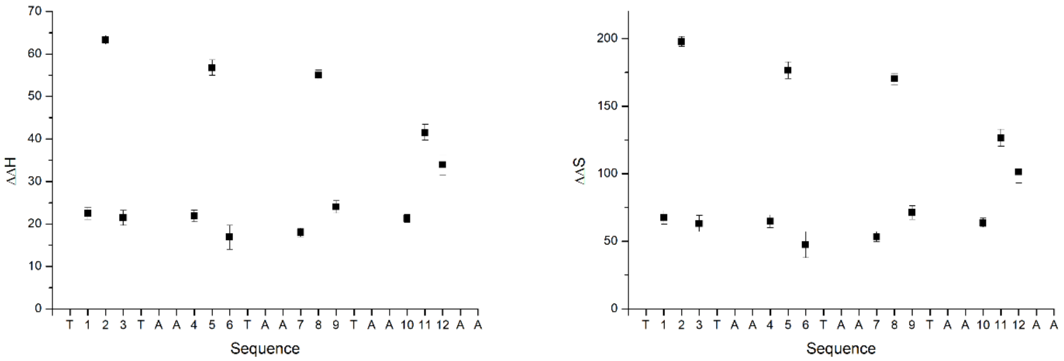

Figure S12. Difference in enthalpy and entropy change calculated for each of the mutated hTeloCTx oligonucleotides compared to the unmodified control sequence (line). Cytosines are numbered from 5′- to 3′-

Thymine Screen

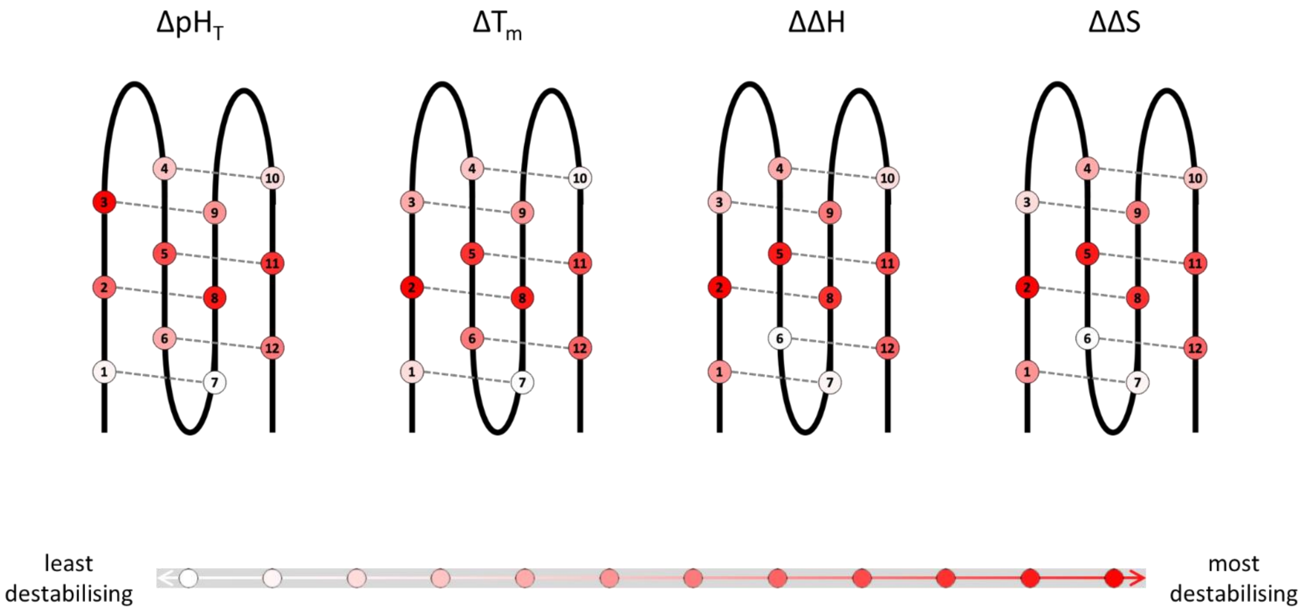

Figure S13. Summary figure showing the effect of substitution of each cytosine with thymine at each position on the transitional pH, melting temperature and thermodynamic characteristics of the i-motifs formed by the modified hTeloCTx oligonucleotides.

I-MOTIFS AND METHYLATION PROFILES

Table S8: i-Motif forming sequences, characterization and methylation profiles in MCF-7 and MCF-10A cell lines

Name	Sequence 5'-3'	Methylation profiles from bisulphite sequencing data		i-Motif Type
		MCF-7	MCF-10A	
AC017019.1	CCC-CCC-TCC-CCC-CCT-CCC-CCC-TCC-CCC-C	CCC-CCC-TCC-CCC-CCT-CCC-CCC-TCC-CCC-C	CCC-CCC-TCC-CCC-CCT-CCC-CCC-TCC-CCC-C	Neutral
AC018878.3	CCC-CCA-CCC-CCA-GCC-CCC-TTT-CCC-CC	CCC-CCA-CCC-CCA-GCC-CCC-TTT-CCC-C5mC	CCC-CCA-CCC-CCA-GCC-CCC-TTT-CCC-C5mC	Neutral
CAMK2G	CCC-CCA-GGC-CCC-GCC-AGT-CCC-CCC-CCC-CGC-CCG-GCC-CCC-GGC-CCG-CCC-CC	CCC-CCA-GGC-CCC-GCC-AGT-CCC-CCC-CCC-CGC-CCG-GCC-CCC-GGC-CCG-CCC-CC	CCC-CCA-GGC-CCC-GCC-AGT-CCC-CCC-CCC-CGC-CCG-GCC-CCC-GGC-CCG-CCC-CC	Neutral
DAP	CCC-CCG-CCC-CCG-CCC-CCG-CCC-CCG-CCC-CC	CCC-CCG-CCC-CCG-CCC-CCG-CCC-CCG-CCC-CC	CCC-CCG-CCC-CCG-CCC-CCG-CCC-CCG-CCC-CC	Neutral
DRP2	CCC-CCT-CTT-CCC-CTC-TCC-CCC-TCT-CCC-CCT-CTC-TCC-CTC-TTC-CCC-CTC-TCC-TTG-TCT-CCTTCT-CTC-CCC-C	CCC-CCT-CTT-CCC-CTC-TCC-CCC-TCT-CCC-CCT-CTC-TCC-CTC-TTC-CCC-CTC-TCC-TTG-TCT-CCTTCT-CTC-CCC-C	CCC-CCT-CTT-CCC-CTC-TCC-CCC-TCT-CCC-CCT-CTC-TCC-CTC-TTC-CCC-CTC-TCC-TTG-TCT-CCT-TCT-CTC-CCC-C	Acidic
DUX4L22	CCC-CCG-AAA-CGC-GCC-CCC-CTC-CCC-CCT-CCC-CCC-TCT-CCC-CC	CCC-CCG-AAA-CGC-GCC-CCC-CTC-CCC-CCT-CCC-CCC-TCT-CCC-CC	4 x 100% matches (in close range)<br>1) 135480248-135480288: CCC-CCG-AAA-CGC-GCC-CCC-CTC-C5mCC-CCT-CCC-CCC-TCT-CCC-CC<br>2) 135483558-135483598: CCC-CCG-AAA-CGC-GCC-CCC-CTC-CC5mCC-CCT-CCC-CCC-TCT-CCC-CC<br>3) 135486867-135486907: CCC-CCG-AAA-CGC-GCC-CCC-CTC-CCC-CCT-CCC-CCC-TCT-CCC-CC<br>4) 135493476-135493516: CCC-CCG-AAA-CGC-GCC-CCC-CTC-CCC-CCT-CCC-CCC-TCT-CCC-CC	Neutral

GH2	CCC-CCA-CCC-CCA-CCC-CCA-TCC-CCA-CGC-CCC-GCC-CCC-GCC-CCC	CCC-CCA-CCC-CCA-CCC-CCA-TCC-CCA-CGC-CCC-GCC-CCC-GCC-CCC	CCC-CCA-CCC-CCA-CCC-CCA-TCC-CCA-CGC-CCC-GCC-CCC-GCC-CCC	Neutral
HIC2	CCC-CCG-GGA-CAG-GGA-CCC-TGG-CCC-CCC-CCG-ACA-GGC-TGA-CGC-CCA-CCC-CCT-CAA-ACT-CTG-GTG-GAC-TTA-CCC-CC	CCC-CCG-GGA-CAG-GGA-CCC-TGG-CCC-CCC-CCG-ACA-GGC-TGA-CGC-CCA-CCC-CCT-CAA-ACT-CTG-GTG-GAC-TTA-CCC-CC	CCC-CCG-GGA-CAG-GGA-CCC-TGG-CCC-CCC-CCG-ACA-GGC-TGA-CGC-CCA-CCC-CCT-CAA-ACT-CTG-GTG-GAC-TTA-CCC-CC	Acidic
HOXD10	CCC-CCC-CCC-CCT-CCC-CCG-CGG-CCC-CC	CCC-CCC-CCC-CCT-CCC-CCG-CGG-CCC-CC	CCC-CCC-CCC-CCT-CCC-C5mCG-CGG-CCC-C5mC	Neutral
JAZF1	CCC-CCC-CCG-CCC-CCG-CCC-CCG-CCC-TCC-CCC-C	CCC-CCC-CCG-CCC-CCG-CCC-CCG-CCC-TCC-CCC-C	CCC-CCC-CCG-CCC-CCG-CCC-CCG-CCC-TCC-CCC-C	Neutral
MSMO1	CCC-CCG-CCC-CCG-CCC-CCG-CCC-CC	1) 820224- 820246 CCC-CCG-CCC-CCG-CCC-CCG-CCC-CC<br>2) 166248041-166248063 CCC-CCG-CCC-CCG-CCC-CCG-CCC-CC<br>3) 42198880-42198902 CCC-CCG-CCC-CCG-CCC-CCG-CCC-CC	1) 820224-820246 CCC-CCG-CCC-CCG-CCC-CCG-CCC-CC<br>2) 166248041-166248063 CCC-CCG-CCC-CCG-CCC-C5mCG-CCC-CC<br>3) 42198880-42198902 CCC-C5mCG-CCC-CCG-CCC-C5mCG-CCC-C5mC	Acidic
NFATC1	CCC-CCG-TTT-CCC-CCG-CCA-GCC-CCA-GCG-CCC-CCC-TGC-CCG-GCC-CCC	CCC-CCG-TTT-CCC-CCG-CCA-GCC-CCA-GCG-CCC-CCC-TGC-C5mCG-GCC-CCC	CCC-CCG-TTT-CCC-CCG-CCA-GCC-CCA-GCG-CCC-CCC-TGC-CCG-GCC-CCC	Neutral
PIM1	CCC-CCG-ACG-CGC-CCC-CCA-ACA-CAC-AAA-CCC-CCA-GAA-TCC-GCC-CCC	CCC-CCG-ACG-5mCGC-CCC-CCA-ACA-CAC-AAA-CCC-CCA-GAA-TCC-GCC-CCC (20% methylation)	CCC-CCG-ACG-CGC-CCC-CCA-ACA-CAC-AAA-CCC-CCA-GAA-TCC-GCC-CCC	Neutral
PLCB2	CCC-CCG-CCT-CTT-CTG-GAG-GCC-CCC-GCC-CCC-ACC-CCC	CCC-C5mCG-CCT-CTT-CTG-GAG-GCC-CC5mC-GCC-CCC-ACC-CCC	CCC-C5mCG-CCT-CTT-CTG-GAG-GCC-CC5mC-GCC-CCC-ACC-CCC	Neutral
QSOX1	CCC-CCG-CCC-CCG-AGC-CCC-CGC-CCC-C	CCC-CCG-CCC-CCG-AGC-CCC-CGC-CCC-C	CCC-CCG-CCC-CCG-AGC-CCC-CGC-CCC-C	Neutral
RAE1	CCC-CCC-GCC-CCC-CCC-GCC-CCC-CCG-CGC-CGC-CCC-CCC-CCG-CCC-CCC-GCC-CCC-GTC-CCC-CCG-CCC-CCC-CCG-CCC-CCC-CCG-CCC-CCC-	CCC-CCC-GCC-CCC-CCC-GCC-CCC-CCG-CGC-CGC-CCC-CCC-CCG-CCC-CCC-GCC-CCC-GTC-CCC-CCG-	CCC-CCC-GCC-CCC-CCC-GCC-CCC-CCG-CGC-CGC-CCC-CCC-CCG-CCC-CCC-GCC-CCC-GTC-CCC-CCG-CCC-CCC-CCG-CCC-CCC-CCG-CCC-	Neutral

	GTC-CCC-CCG-CCC-CCC-CGC-CCC-CCC-GTC-CCC-CC	CCC-CCC-CCG-CCC-CCC-CCG-CCC-CCC-GTC-CCC-CCG-CCC-CCC-CGC-CCC-CCC-GTC-CCC-CC	CCC-GTC-CCC-CCG-CCC-CCC-CGC-CCC-CCC-GTC-CCC-5mCC	
RUNX1-1	CCC-CCC-CCG-CAC-CCC-TTC-CCC-CGG-CCC-CCC-C	CCC-CCC-CCG-CAC-CCC-TTC-CCC-CGG-CCC-CCC-C	CCC-CCC-CCG-CAC-CCC-TTC-CCC-CGG-CCC-CCC-C<br><br>G-rich strand is methylated	Acidic
RUNX1-2	CCC-CCC-TCC-CCC-TGC-CTC-TCC-CTC-CCC-CCT-TTC-CCC	CCC-CCC-TCC-CCC-TGC-CTC-TCC-CTC-CCC-CCT-TTC-CCC	CCC-CCC-TCC-CCC-TGC-CTC-TCC-CTC-CCC-CCT-TTC-CCC	Acidic
RUNX1-3	CCC-CCC-TTT-CCC-CTG-CCC-CCC-CTG-CCT-CCC-CC	CCC-CCC-TTT-CCC-CTG-CCC-CCC-CTG-CCT-CCC-CC	CCC-CCC-TTT-CCC-CTG-CCC-CCC-CTG-CCT-CCC-CC	Acidic
SHANK1b	CCC-CCC-TCC-CCC-CAC-CCC-CCA-CCC-CCC-C	CCC-CCC-TCC-CCC-CAC-CCC-CCA-CCC-CCC-C	CCC-CCC-TCC-CCC-CAC-CCC-CCA-CCC-CCC-C	Neutral
SHANK3	CCC-CCG-CCT-CCG-GCG-CAG-CCC-CCT-CGC-CAC-CCC-CGC-TTC-CCT-CCC-GTC-TCA-GGC-CCC-CTC-CCC-CCG-CCG-CCC-CCG-CCC-CC	CCC-CCG-CCT-CCG-GCG-CAG-CCC-CCT-CGC-CAC-CCC-CGC-TTC-CCT-CCC-GTC-TCA-GGC-CCC-CTC-CCC-CCG-CCG-CCC-CCG-CCC-CC	CCC-CCG-CCT-CCG-GCG-CAG-CCC-CCT-CGC-CAC-CCC-CGC-TTC-CCT-CCC-GTC-TCA-GGC-CCC-CTC-CCC-CCG-CCG-CCC-CCG-CCC-CC	Acidic
SHANK3b	CCC-CCC-GCA-CCG-AGG-CCT-AGG-ACT-CCC-CCC-CCC-AAC-CCC-GTC-ACA-GCC-CCC-CAG-ACC-CCC-GCC-CCG-TGG-CTC-GGC-CCC-C	CCC-CCC-GCA-CCG-AGG-CCT-AGG-ACT-CCC-CCC-CCC-AAC-CCC-GTC-ACA-GCC-CCC-CAG-ACC-CCC-GCC-CCG-TGG-CTC-GGC-CCC-C	CCC-CCC-GCA-CCG-AGG-CCT-AGG-ACT-CCC-CCC-CCC-AAC-CCC-GTC-ACA-GCC-CCC-CAG-ACC-CC5mC-GCC-CCG-TGG-CTC-GGC-CCC-5mC	Acidic
SNORD112	CCC-CCC-CCC-GCC-CCC-CAC-CCC-CCC-ACC-CCC-CCC-CCC	CCC-CCC-CCC-GCC-CCC-CAC-CCC-CCC-ACC-CCC-CCC-CCC	CCC-CCC-CCC-GCC-CCC-CAC-CCC-CCC-ACC-CCC-CCC-CCC	Neutral
SOX1	CCC-CCT-GCA-GGC-CCC-CCT-GCG-CCT-CCC-CCC-CCC-CGC-CAC-TGG-CGC-CTG-GCT-TCC-CCC	CCC-CCT-GCA-GGC-CCC-CCT-G5mCG-CCT-CCC-CCC-CCC-5mCGC-CAC-TGG-5mCGC-CTG-GCT-TCC-CC5mC	CCC-CCT-GCA-GGC-CCC-CCT-G5mCG-CCT-CCC-CCC-CCC-CGC-CAC-TGG-CGC-CTG-GCT-TCC-CC5mC	Neutral
STX17	CCC-CCG-CCC-CCG-CCC-CCG-CCC-CGC-AGG-GCC-CCC	CCC-CCG-CCC-CCG-CCC-CCG-CCC-CGC-AGG-GCC-CCC	CCC-CCG-CCC-CCG-CCC-CCG-CCC-CGC-AGG-GCC-CCC	Neutral
Tandem Re-peat	CCC-CCC-GTG-TCG-CTG-TTC-CCC-CCG-TGT-CGC-TGT-TCC-CCC-CGT-GTC-GCT-GTT-CCC-CCC	CCC-CCC-GTG-TCG-CTG-TTC-CCC-CCG-TGT-CGC-TGT-TCC-CCC-CGT-GTC-GCT-GTT-CCC-CCC<br><br>G-rich strand is methylated	CCC-CCC-GTG-TCG-CTG-TTC-CCC-CCG-TGT-CGC-TGT-TCC-CCC-CGT-GTC-GCT-GTT-CCC-CCC	Acidic

(LA16c-OS12.2)			G-rich strand is methylated	
WNT7A	CCC-CCG-CCC-CTC-CCT-CCT-TTC-CCC-CGT-CCC-TCC-CCC-GCC-CCC-TCC-CCC	CCC-C5mCG-CCC-CTC-CCT-CCT-TTC-CCC-5mCGT-CCC-TCC-CCC-GCC-CCC-TCC-CC5mC	CCC-CCG-CCC-CTC-CCT-CCT-TTC-CCC-CGT-CCC-TCC-CCC-GCC-CCC-TCC-CCC	Neutral
ZBTB7B	CCC-CCC-ATC-CCT-CCC-CTC-CCT-CCC-CCC-GCC-CCT-GCC-ACC-CCC-CAA-ACT-CCC-CCC-CCC-C	CCC-CCC-ATC-CCT-CCC-CTC-CCT-CCC-CCC-GCC-CCT-GCC-ACC-CCC-CAA-ACT-CCC-CCC-CCC-C	CCC-CCC-ATC-CCT-CCC-CTC-CCT-CCC-CCC-GCC-CCT-GCC-ACC-CCC-CAA-ACT-CCC-CCC-CCC-C	Neutral
ZFP41	CCC-CCA-GCC-CCC-GCC-GAC-CCC-CAG-CTC-CCG-CCT-CCG-CCG-ACC-CCC-AGC-CCC-C	CCC-CCA-GCC-CCC-GCC-GAC-CCC-CAG-CTC-CCG-CCT-CCG-CCG-ACC-CCC-AGC-CCC-C<br><br>G-rich strand is methylated	CCC-CCA-GCC-CCC-GCC-GAC-CCC-CAG-CTC-CCG-CCT-CCG-CCG-ACC-CCC-AGC-CCC-C<br><br>G-rich strand is methylated	Neutral
ZNF480	CCC-CCG-CCC-CCG-CCC-CCG-CCC-CC	CCC-CCG-CCC-CCG-CCC-CCG-CCC-CC	CCC-CCG-CCC-CCG-CCC-CCG-CCC-CC	Acidic
hTelo	CCC-TAA-CCC-TAA-CCC-TAA-CCC-T	CCC-TAA-CCC-TAA-CCC-TAA-CCC-T	CCC-TAA-CCC-TAA-CCC-TAA-CCC-T	Acidic
c-MYC	CCC-CAC-CTT-CCC-CAC-CCT-CCC-CAC-CCT-CCC-C	CCC-CAC-CTT-CCC-CAC-CCT-CCC-CAC-CCT-CCC-C	CCC-CAC-CTT-CCC-CAC-CCT-CCC-CAC-CCT-CCC-C	Acidic
bcl-2	CAG-CCC-CGC-TCC-CGC-CCC-CTT-CCT-CCC-GCG-CCC-GCC-CCT	CAG-CCC-CGC-TCC-CGC-CCC-CTT-CCT-CCC-GCG-CCC-GCC-CCT	CAG-CCC-CGC-TCC-CGC-CCC-CTT-CCT-CCC-GCG-CCC-GCC-CCT	Acidic
RET	CCG-CCC-CCG-CCC-CGC-CCC-GCC-CCT-A	CCG-CCC-CCG-CCC-CGC-CCC-GCC-CCT-A	CCG-CCC-CCG-CCC-CGC-CCC-GCC-CCT-A	Acidic
VEGF-A	GAC-CCC-GCC-CCC-GGC-CCG-CCC-CGG	GAC-CCC-GCC-CCC-GGC-CCG-CCC-CGG	GAC-CCC-GCC-CCC-GGC-CCG-CCC-CGG	Acidic
c-ki-ras	GCT-CCC-TCC-CTC-CCT-CCT-TCC-CTC-CCT-CCC	GCT-CCC-TCC-CTC-CCT-CCT-TCC-CTC-CCT-CCC	GCT-CCC-TCC-CTC-CCT-CCT-TCC-CTC-CCT-CCC	Acidic
PDGF-A	CCG-CGC-CCC-TCC-CCC-GCC-CCC-GCC-CCC-GCC-CCC-CCC-CCC-CC	CCG-CGC-CCC-TCC-CCC-GCC-CCC-GCC-CCC-GCC-CCC-CCC-CCC-CC	CCG-CGC-CCC-TCC-CCC-GCC-CCC-GCC-CCC-GCC-CCC-CCC-CCC-CC	Acidic
c-myb	TCC-TCC-TCC-TCC-TTC-TCC-TCC-TCC-TCC-GTG-TCC-TCC-TCC-TCC	TCC-TCC-TCC-TCC-TTC-TCC-TCC-TCC-TCC-GTG-TCC-TCC-TCC-TCC	TCC-TCC-TCC-TCC-TTC-TCC-TCC-TCC-TCC-GTG-TCC-TCC-TCC-TCC	Acidic

hTERT	CCC-CGC-CCC-GTC-CCG-ACC-CCT-CCC-GGG-TCC-CCG-GCC-CAG-CCC-CCA-CCG-GGC-CCT-CCC-AGC-CCC-TCC-CC	CCC-CGC-CCC-GTC-CCG-ACC-CCT-CCC-GGG-TCC-CCG-GCC-CAG-CCC-CCA-CCG-GGC-CCT-CCC-AGC-CCC-TCC-CC<br><br>G-rich strand is methylated	CCC-CGC-CCC-GTC-CCG-ACC-CCT-CCC-GGG-TCC-CCG-GCC-CAG-CCC-CCA-CCG-GGC-CCT-CCC-AGC-CCC-TCC-CC	Acidic
HIF-1a	CGC-GCT-CCC-GCC-CCC-TCT-CCC-CTC-CCC-GCG-CGC-CCG-AGC-GCG-CCT-CCG-CCC-TTG-CCC-GCC-CCC-TG	CGC-GCT-CCC-GCC-CCC-TCT-CCC-CTC-CCC-GCG-CGC-CCG-AGC-GCG-CCT-CCG-CCC-TTG-CCC-GCC-CCC-TG	CGC-GCT-CCC-GCC-CCC-TCT-CCC-CTC-CCC-GCG-CGC-CCG-AGC-GCG-CCT-CCG-CCC-TTG-CCC-GCC-CCC-TG	Neutral
c-jun	TAA-CCC-CCT-CCC-CCT-CCC-CCC-TTT-AAT	TAA-CCC-CCT-CCC-CCT-CCC-CCC-TTT-AAT	TAA-CCC-CCT-CCC-CCT-CCC-CCC-TTT-AAT	Acidic
ILPR	TGT-CCC-CAC-ACC-CCT-GTC-CCC-ACA-CCC-CTG-T	TGT-CCC-CAC-ACC-CCT-GTC-CCC-ACA-CCC-CTG-T<br><br>G-rich strand is methylated	TGT-CCC-CAC-ACC-CCT-GTC-CCC-ACA-CCC-CTG-T	Acidic
n-MYC	ACC-CCC-TGC-ATC-TGC-ATG-CCC-CCT-CCC-ACC-CCC-T	ACC-CCC-TGC-ATC-TGC-ATG-CCC-CCT-CCC-ACC-CCC-T	ACC-CCC-TGC-ATC-TGC-ATG-CCC-CCT-CCC-ACC-CCC-T	Acidic
SMARCA4	TCC-CTT-GCT-ATC-CCT-GTC-CTG-CCT-CGC-CCT-TGG-TCA-TGA-ACC-CC	TCC-CTT-GCT-ATC-CCT-GTC-CTG-CCT-5mCGC-CCT-TGG-TCA-TGA-ACC-CC	TCC-5mCTT-GCT-ATC-CCT-GTC-CTG-CCT-5mCGC-CCT-TGG-TCA-TGA-ACC-CC	Acidic
C9ORF72	d(GGCCCC)$_n$	1) **chr9:132,331,491-132,331,514:** GGC-CCC-GGC-CCC-GGC-CCC-GGC-CCC<br><br>2) **chr9:82,478,642-82,478,665:** GGC-CC5mC-GGC-CC5mC-GGC-CC5mC-GGC-CC5mC	1) **chr9:132,331,491-132,331,514**: GGC-CCC-GGC-CCC-GGC-CCC-GGC-CCC<br><br>G-rich strand is methylated<br><br>2) **chr9:82,478,642-82,478,66**5: GGC-CC5mC-GGC-CC5mC-GGC-CC5mC-GGC-CC5mC	Neutral

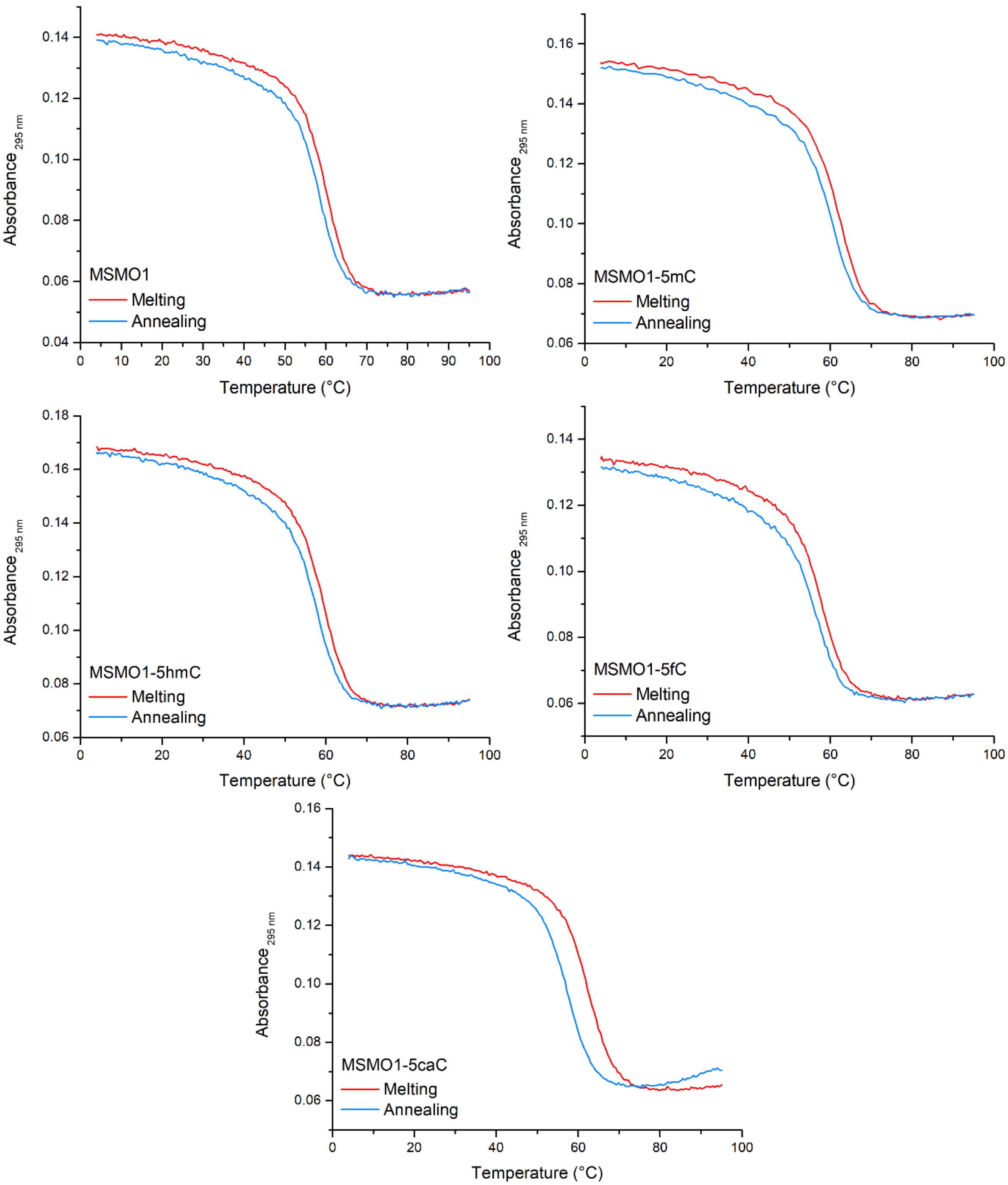

Figure S14. UV melting and annealing curves for unmodified and epigenetically modified MSMO1. All oligonucleotides were diluted to a final concentration of 2.5 μM in 10 mM sodium cacodylate with 100 mM sodium chloride at pH 5.5.

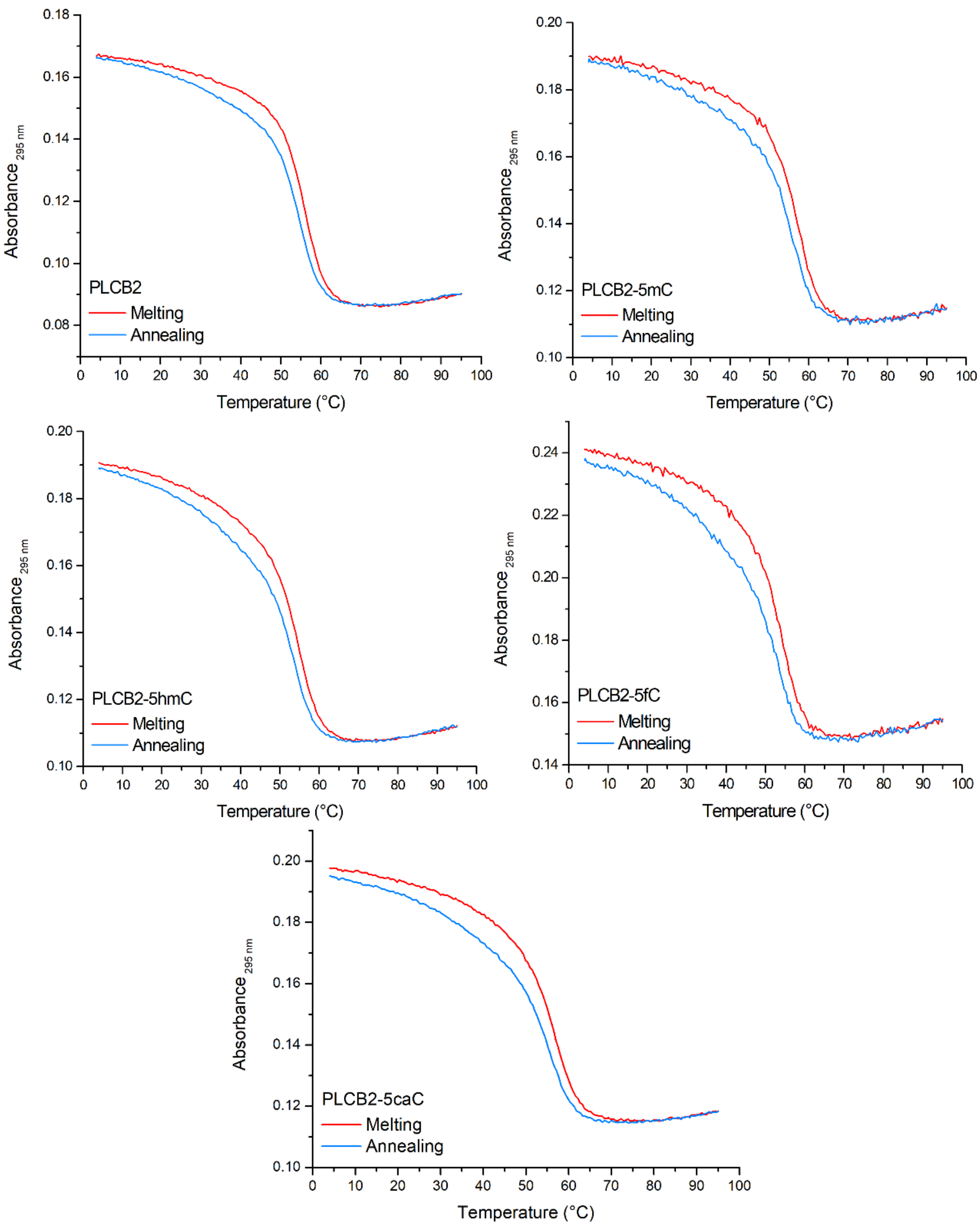

Figure S15. UV melting and annealing curves for unmodified and epigenetically modified PLCB2. All oligonucleotides were diluted to a final concentration of 2.5 μM in 10 mM sodium cacodylate with 100 mM sodium chloride at pH 5.5.

Table S9. Melting temperatures of the unmodified control sequence and each of the epigenetically modified MSMO1 and PLCB2 oligonucleotides. * = p-value ≤ 0.05

	T_m (°C)	
Sequence	MSMO1	PLCB2
Unmodified Control	60.8 ± 0.79	56.6 ± 1.39
5mC	62.3 ± 0.58	57.9 ± 0.61
5hmC	59.0 ± 1.28	54.7 ± 1.10
5fC	57.4 ± 0.64*	53.9 ± 1.65
5caC	62.1 ± 1.01	56.7 ± 0.58

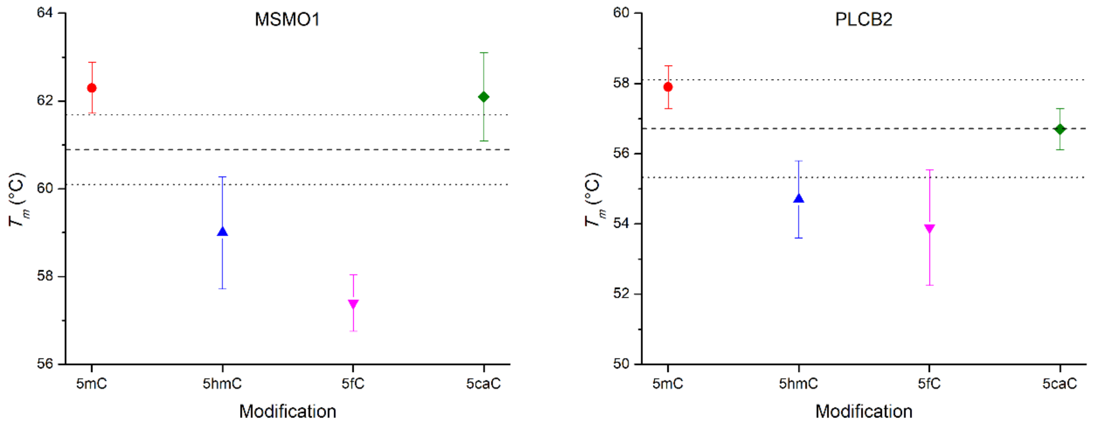

Figure S16. Melting temperature of each of the epigenetically modified (● 5-methylcytosine; ▲ 5-hydroxymethylcytosine; ▼ 5-formylcytosine; ◆ 5-carboxylmethylcytosine) MSMO1 and PLCB2 oligonucleotides compared to the unmodified control sequence (line).

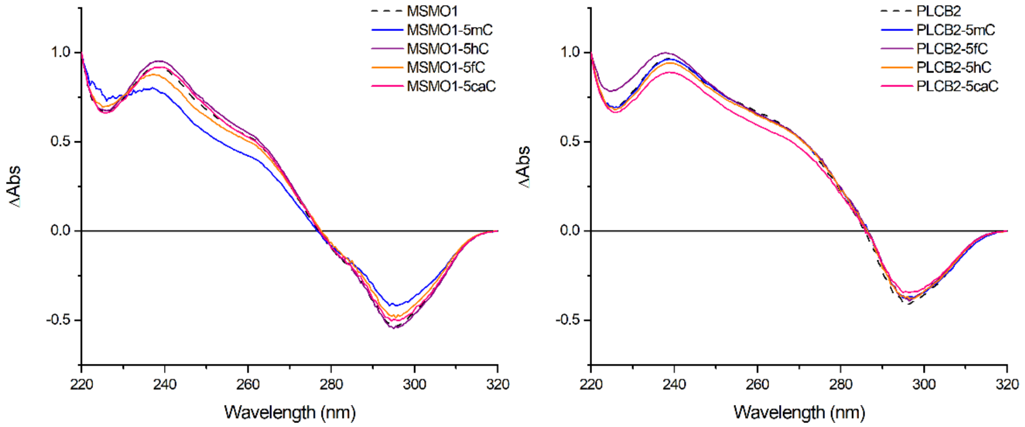

Figure S17. Thermal difference spectra of unmodified and epigenetically modified MSMO1 and PLCB2 oligonucleotides.

Table S10. Enthalpy and and entropy for each unmodifed and epigenetically modified MSMO1 and PLCB2 oligonucleotide calculated using the UV-melt curves.

	ΔH		ΔS	
Sequence	MSMO1	PLCB2	MSMO1	PLCB2
Unmodified Control	-89.0 ± 1.6	-86.2 ± 0.6	-249.3 ± 4.6	-262.0 ± 1.8
5mC	-82.7 ± 1.9	-81.9 ± 3.9	-247.0 ± 5.6	-248.2 ± 11.9
5hmC	-79.7 ± 1.47	-81.8 ± 2.0	-239.9 ± 4.5	-250.1 ± 6.1
5fC	-78.5 ± 4.1	-78.3 ± 4.0	-237.2 ± 12.1	-239.7 ± 11.9
5caC	-82.3 ± 15.9	-76.9 ± 0.9	-254.2 ± 47.8	-233.4 ± 2.8

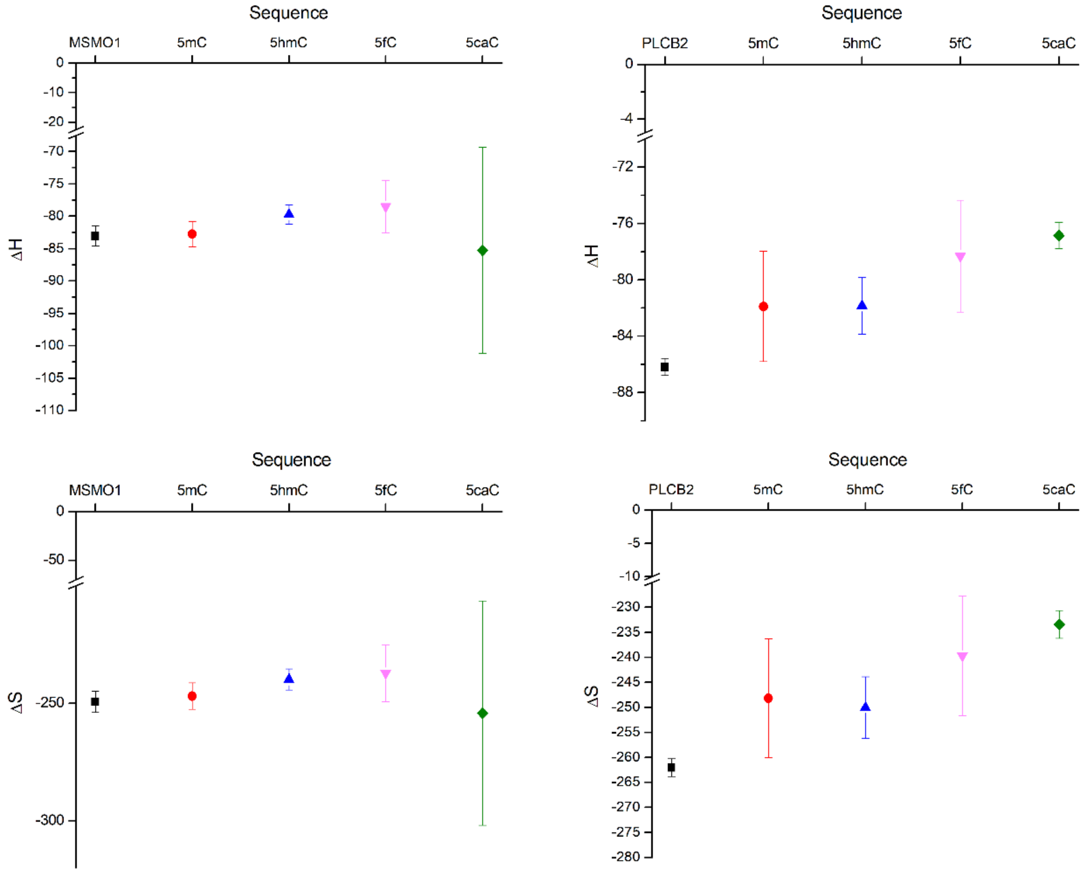

Figure S18. Enthalpy (upper panels) and entropy (lower panels) of each of the unmodified and epigenetically modified (■ unmodified; ● 5-methylcytosine; ▲ 5-hydroxymethylcytosine; ▼ 5-formylcytosine; ◆ 5-carboxylmethylcytosine) MSMO1 and PLCB2 oligonucleotides compared to the unmodified control sequence (line).

CIRCULAR DICHROISM OF MSMO1 AND PLCB2

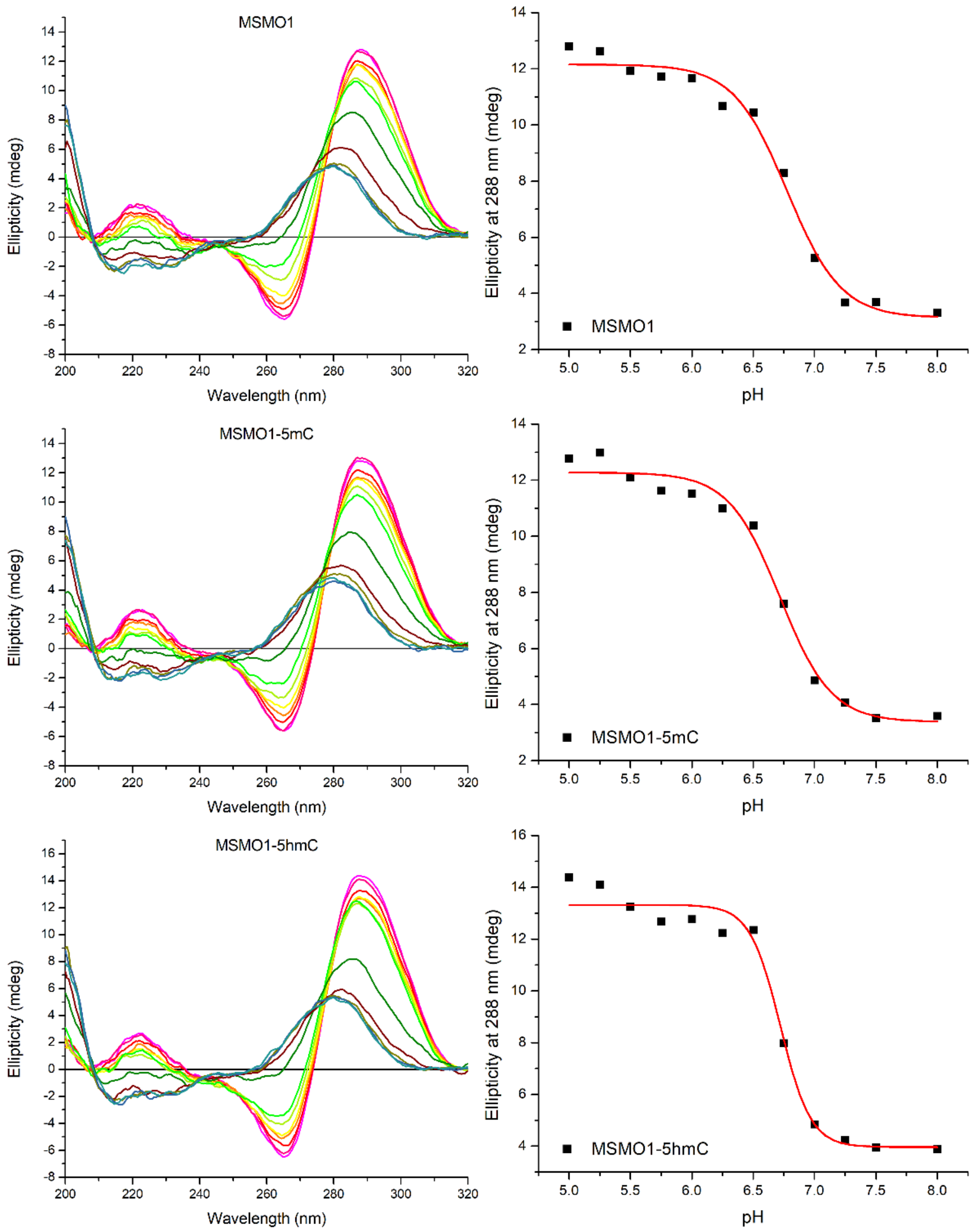

Figure S19A. CD spectra for epigenetically modified MSMO1. All oligonucleotides were diluted to a final concentration of 10 µM in 10 mM sodium cacodylate with 100 mM sodium chloride at the indicated pH ■pH 5.0; ■pH 5.25; ■pH 5.5; ■pH 5.75; ■pH 6.0; ■pH 6.25; ■pH 6.5; ■pH 6.75; ■pH 7.0; ■pH 7.25; ■pH 7.5; and ■pH 8.0.

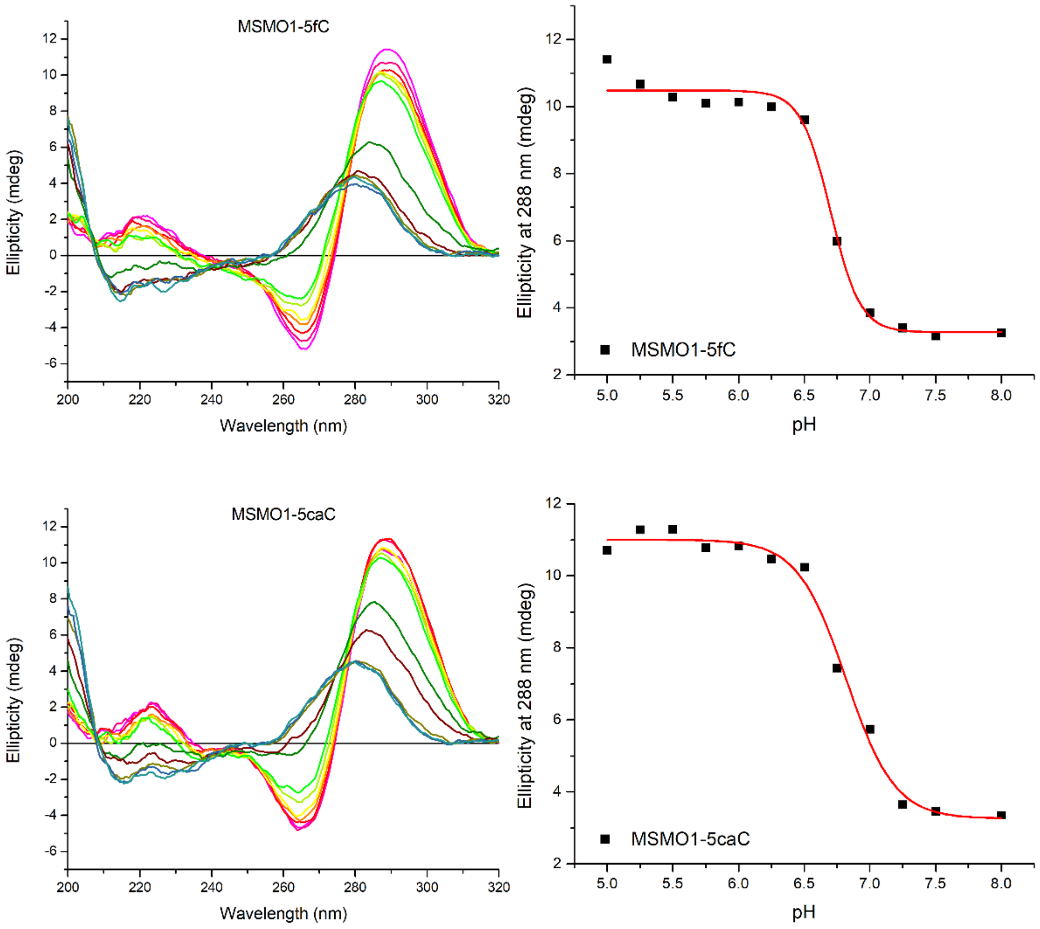

Figure S19B. CD spectra for epigenetically modified MSMO1. All oligonucleotides were diluted to a final concentration of 10 µM in 10 mM sodium cacodylate with 100 mM sodium chloride at the indicated pH pH 5.0; pH 5.25; pH 5.5; pH 5.75; pH 6.0; pH 6.25; pH 6.5; pH 6.75; pH 7.0; pH 7.25; pH 7.5; and pH 8.0.

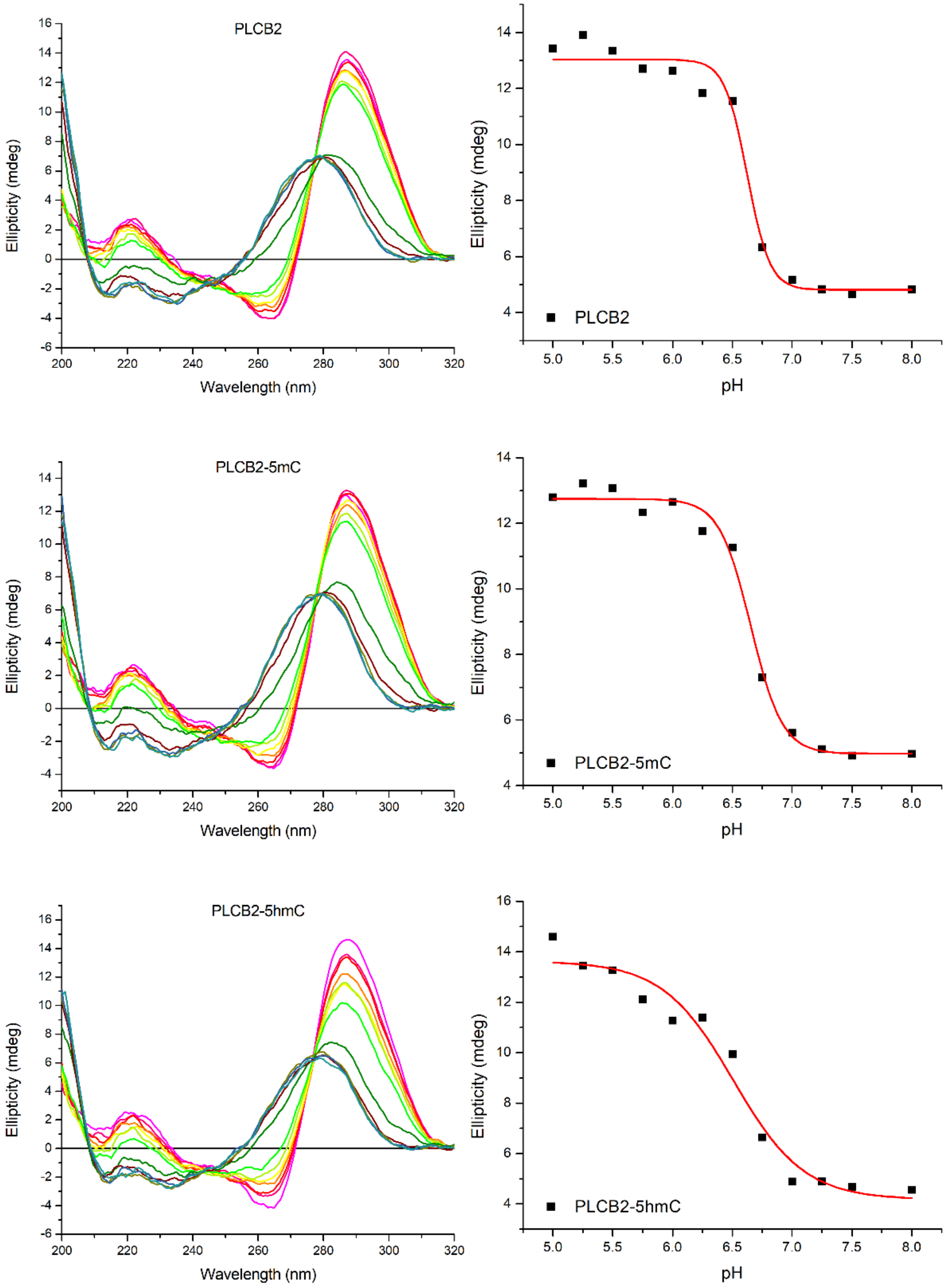

Figure S20A. CD spectra for epigenetically modified PLCB2. All oligonucleotides were diluted to a final concentration of 10 μM in 10 mM sodium cacodylate with 100 mM sodium chloride at the indicated pH ■pH 5.0; ■pH 5.25; ■pH 5.5; ■pH 5.75; ■pH 6.0; ■pH 6.25; ■pH 6.5; ■pH 6.75; ■pH 7.0; ■pH 7.25; ■pH 7.5; and ■pH 8.0.

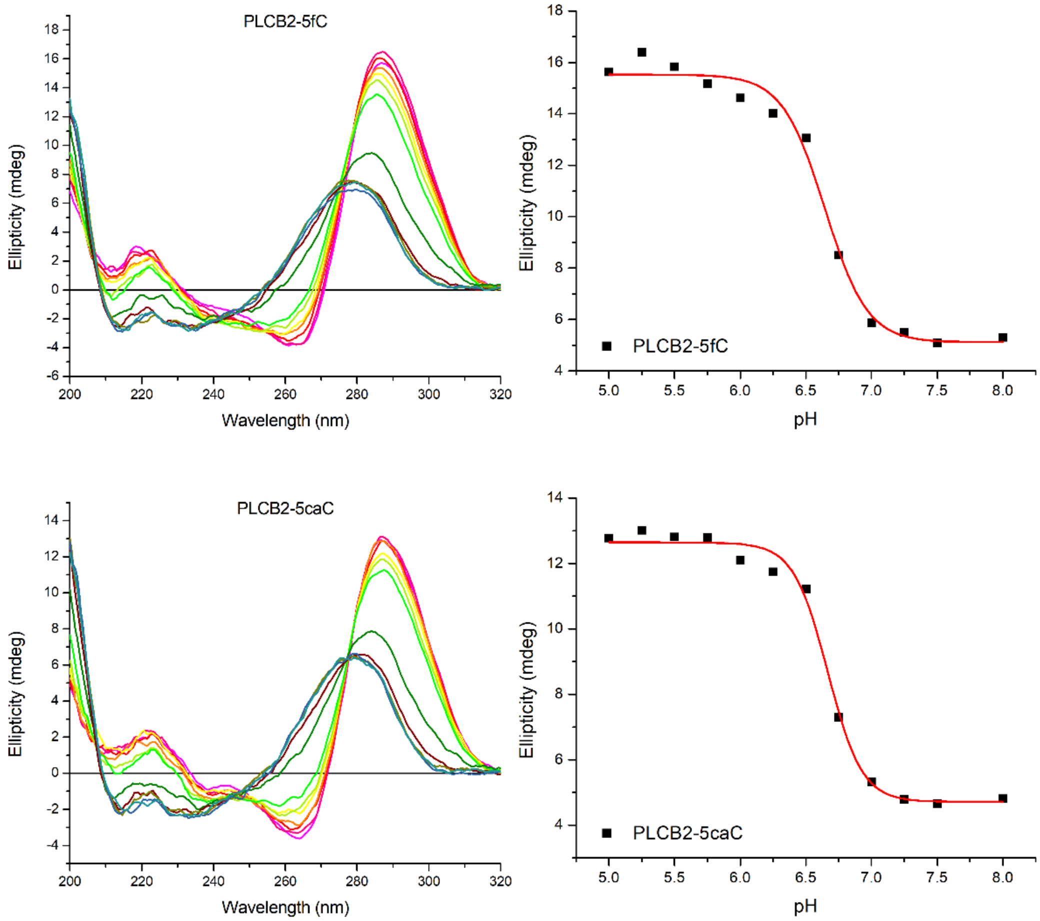

Figure S20B. CD spectra for epigenetically modified PLCB2. All oligonucleotides were diluted to a final concentration of 10 μM in 10 mM sodium cacodylate with 100 mM sodium chloride at the indicated pH ■pH 5.0; ■pH 5.25; ■pH 5.5; ■pH 5.75; ■pH 6.0; ■pH 6.25; ■pH 6.5; ■pH 6.75; ■pH 7.0; ■pH 7.25; ■pH 7.5; and ■pH 8.0.

Table S11. Transitional pH values for the unmodified control sequence and each of the epigenetically modified MSMO1 and PLCB2 oligonucleotides.

	pH_T	
Sequence	MSMO1	PLCB2
Unmodified Control	6.78 ± 0.04	6.62 ± 0.03
5mC	6.72 ± 0.04	6.65 ± 0.03
5hmC	6.72 ± 0.03	6.51 ± 0.08
5fC	6.70 ± 0.03	6.64 ± 0.03
5caC	6.82 ± 0.03	6.67 ± 0.02

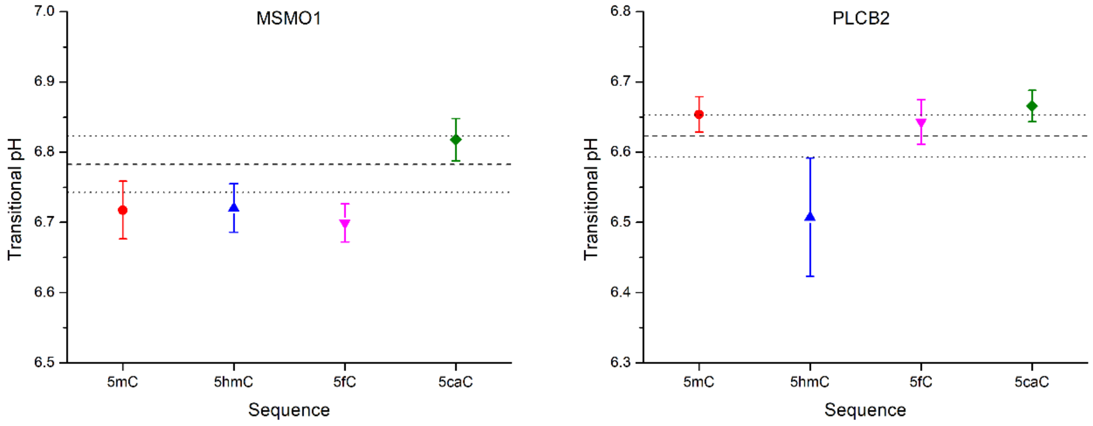

Figure S21. Transitional pH of each of the epigenetically modified (● 5-methylcytosine; ▲ 5-hydroxymethylcytosine; ▼ 5-formylcytosine; ◆ 5-carboxylmethylcytosine) MSMO1 and PLCB2 oligonucleotides compared to the unmodified control sequence (line).
